# Solar irradiance measurements

**DOI:** 10.1007/s41116-025-00040-5

**Published:** 2025-07-11

**Authors:** Greg Kopp

**Affiliations:** Laboratory for Atmospheric and Space Physics, University of Colorado, Boulder, 80303 USA

**Keywords:** Solar irradiance, Total solar irradiance, TSI, Solar constant, Spectral solar irradiance, SSI, Solar radiometry, Solar climate data record, Solar insolation, Top-of-atmosphere flux, Earth-energy balance

## Abstract

The Sun provides nearly all the energy powering the Earth’s climate system, far exceeding all other energy sources combined. The incident radiant energy, the “total solar irradiance,” has been measured by an uninterrupted series of temporally overlapping precision space-borne radiometric instruments since 1978, giving a record spanning more than four 11-year solar cycles. Short-term total-irradiance variations exceeding 0.1% can occur over a few days while variations of ~ 0.1% in-phase with the solar cycle are typical. Knowledge of solar variability on timescales longer than the current multi-decadal space-borne record relies on solar-activity proxies and models, which indicate similar-magnitude changes over centuries. Spectrally resolved space-borne irradiance measurements in the ultraviolet have been acquired continuously since 1979, while measurements contiguously spanning the near-ultraviolet to the near-infrared began in 2003. The combination of long-term total- and spectral-irradiance measurements helps determine both the solar causes of irradiance variability, which are primarily due to solar-surface magnetic-activity regions such as sunspots and faculae, and the mechanisms by which solar variability affects the Earth’s climate system, with global and regional temperatures responding to variability at solar-cycle and longer timescales. To better understand these solar influences, the most modern total-irradiance instruments are approaching the needed climate-driven measurement accuracy and stability requirements for detection of potential long-term solar-variability trends, while the latest spectral-irradiance instruments are beginning to be able to discern solar-cycle variability. Focusing on the space-borne era where such measurements are the most accurate and stable, this article describes solar-irradiance instrument designs, capabilities, and operational methodologies. It summarizes the many total- and spectral-irradiance measurements available and the measured solar variabilities on timescales from minutes to solar cycles and discusses extrapolations via models to longer timescales. Measurement composites and reference spectra are reviewed. Current capabilities and future directions are described along with the climate-driven solar-irradiance measurement requirements.

## Solar irradiance

### Total solar irradiance: where does the Earth get its energy?

The total solar irradiance (TSI) is the spatially and spectrally integrated radiant power per unit area from the Sun at one astronomical unit (AU), or 149,598,500 km, from Sun-center. This solar radiant power provides 99.978% of the total direct and indirect energy sources that power Earth’s climate system, with 99.963% of the total being direct solar radiation (Kren et al. 2017; see Table 1). The next most significant Earth-energy sources are radioactive decay and geothermal activity, although these combined with all other Earth-heating sources are a factor of 2700 lower than the TSI itself.

**Table 1:** Earth energy heating sources (Data from Kren et al. 2017)

Energy source	Heat flux* [W m^−2^]	Relative input
**Solar irradiance**	**340.2**	**1.000E+00**
**Secondary sources of solar origin (total)**	**0.0268**	**7.90E**−**05**
Infrared radiation from the full moon	0.01	2.90E−05
Combustion of coal, oil, and gas (in U.S.)	0.0052	1.50E−05
Dissipation of magnetic storm energy	0.00362	1.10E−05
Airglow emission	0.0036	1.10E−05
Sun's radiation reflected from full moon	0.0018	5.30E−06
Energy generated by solar tidal forces in the atmosphere	0.00168	4.90E−06
Energy dissipated in lightning discharges	4.95E−04	1.50E−06
Auroral emission	3.70E−04	1.10E−06
Zodiacal irradiance	5.67E−05	1.70E−07
Earthshine	1.93E−07	5.70E−10
**Secondary sources of non-solar origin (total)**	**0.0900**	**2.60E**−**04**
Heat flux from Earth's interior	0.09	2.60E−04
Energy generated by lunar tidal forces in the atmosphere	1.96E−05	5.80E−08
Galactic cosmic rays	8.50E−06	2.50E−08
Total radiation from stars	6.78E−06	2.00E−08
Cosmic microwave radiation background	3.13E−06	9.20E−09
Dissipation of mechanical energy from micrometeorites	1.10E−06	3.20E−09
**Total of all secondary energy sources**	**0.1169**	**3.39E**−**04**

*Global average

Bold type indicates the net of other energy sources. The bolded “Secondary sources” are the sums of the subsequent, non-bold energy sources. The total of those two is given by the bold-faced “Total of all secondary energy sources” in the bottom row. The bold-faced “Solar irradiance” in the top row gives the net incoming energy to which other sources can be compared

With the Earth’s energy coming almost completely from the solar irradiance, temperature estimates of the Earth’s climate are straightforward. Treating the Earth as a sphere of radius *R* having gray-body emissivity* e*, it absorbs incident total solar irradiance *S* over its circular cross-sectional area, π*R*^2^. That absorbed energy heats the planet to temperature *T*, causing gray-body emission *eσT*^4^ (where *σ* is the Stefan–Boltzmann constant) that is radiated to space over the entire spherical surface area, 4π*R*^2^. In equilibrium, the absorbed and radiated energies balance such that

1 $$ S \times e\pi R^{{2}} = {4}\pi R^{{2}} \times e\sigma T^{{4}} \Rightarrow T = (S / {4}\sigma )^{{{1}/{4}}} . $$

At an average orbital distance of 1 AU, the 1361 W m^−2^ of TSI incident at the top of the Earth’s atmosphere (see Kopp and Lean 2011; Prša et al. 2016) would give a globally averaged gray-body equilibrium surface temperature of 278 K, or 5 °C. Outward-going emission characteristic of this temperature spectrally peaks in the mid-infrared, whereas the incident solar radiation characteristic of the Sun’s 5772 K blackbody temperature peaks in the visible.

**Fig. 1:**
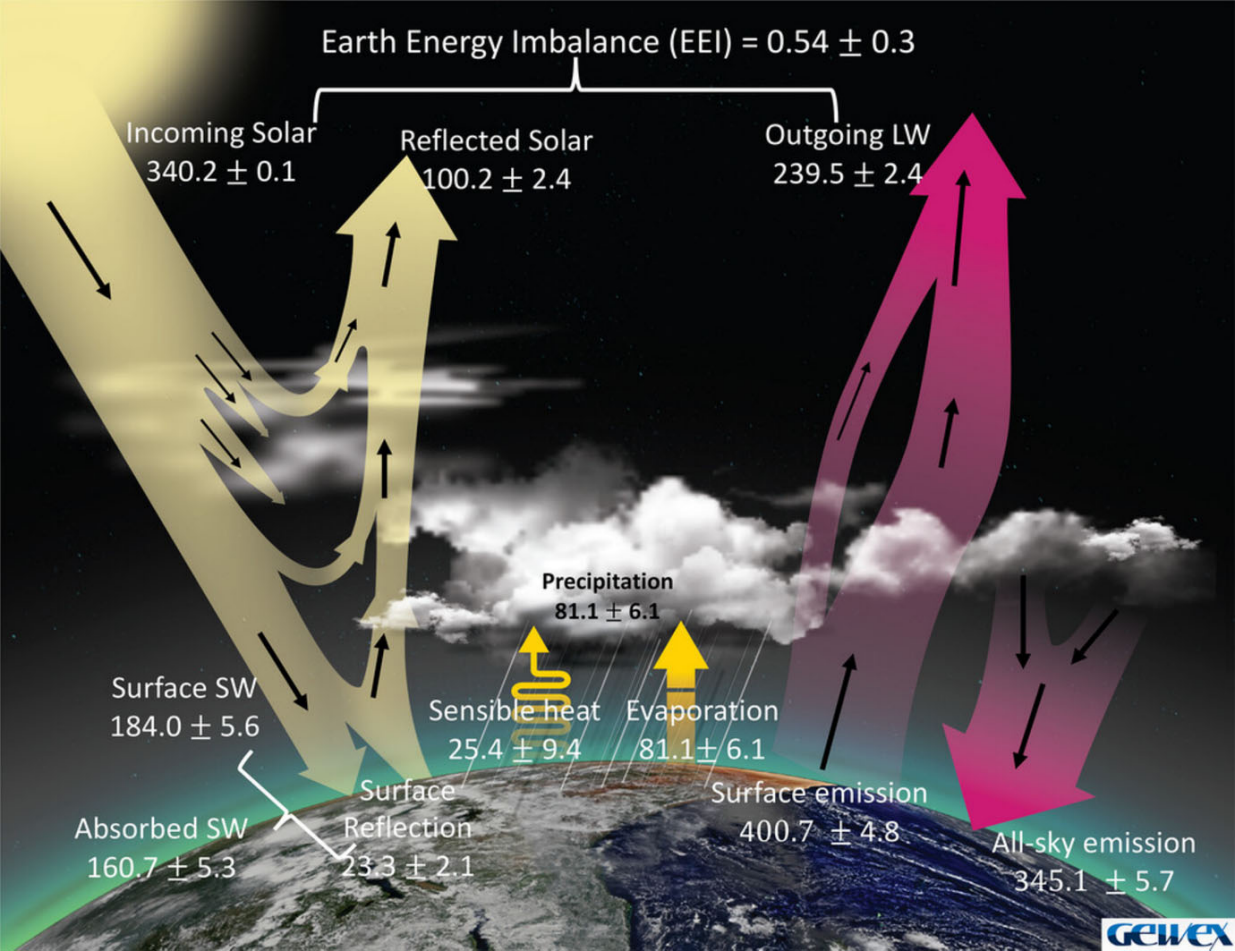
Earth energy imbalance. The Earth-climate system is radiatively in near-equilibrium, with incident short-wave solar radiation approximately balanced by outgoing reflected-solar and long-wave radiation. Units are W m^−2^ averaged over the Earth’s surface. Image reproduced with permission from Stephens et al. (2022), copyright by AMS

The spectral dependence of the Earth’s atmospheric transmission prevents the planet from behaving as a true gray-body, however. Several atmospheric spectral absorption bands, largely due to greenhouse gases such as water vapor, carbon dioxide, and methane, are highly absorptive in the mid-infrared and thus absorb much of the outward-going thermal emission from the Earth’s surface at the mid-infrared wavelengths where that emission is peaked. Those absorbing atmospheric layers then radiate at their own characteristic temperatures, which are cooler than the Earth’s surface and thus radiate less energy. Viewed from space, the outgoing Earth’s radiation is a combination of the solar irradiance directly reflected from the Earth’s surface and atmosphere, thermal emission from the surface at wavelengths where the atmosphere is transparent, and thermal emission from the cooler overlying layers where the atmosphere is absorptive. Figure 1 (from Stephens et al. 2022) summarizes this well with a schematic of the radiative balance that is nearly achieved by the climate system. Incident “short-wave” (SW) solar energy characteristic of a 5772 K blackbody—and thus peaked in the visible—is nearly balanced by reflected solar radiation and the emitted long-wave (LW) radiation characteristic of high-altitude atmospheric gases plus a small contribution from the surface. The combination is non-gray.

Achieving radiative balance despite lower emissions from the cooler high-altitude atmospheric gases requires greater emissions from the Earth’s surface than the gray-body temperature of 278 K calculated above from Eq. (1). This drives surface temperatures to be roughly 10° to 15°C warmer than a gray-body equilibrium temperature would indicate. Energy balance is achieved with a globally averaged surface temperature that is roughly 290 K. Atmospheric greenhouse gases thus affect surface temperatures by altering the spectral-transmission properties of the atmosphere. They do not themselves provide any actual energy to the Earth-climate system, and they are therefore not a direct contributor to the sources in Table 1.

Note from Fig. 1 that roughly one-half of the incident sunlight is absorbed by the Earth’s surface while almost one-third is scattered from the surface or atmosphere back into space due to the Earth’s albedo of 0.29. Any long-term imbalance in radiative equilibrium causes warming or cooling of the planet. Such effects can be caused by variations in surface- and atmospheric-scattering properties, gases in the atmosphere affecting radiative emission and absorption, and, of course, the solar irradiance itself. This article focuses on measurements of the solar irradiance and its variability. For more details on Earth-climate effects from solar-irradiance variability, including the spectrally dependent atmospheric effects, see the thorough reviews by Haigh (2007) and Gray et al. (2010).

### What does the total solar irradiance tell us about the Sun?

The measured TSI is one of the most accurate radiometric values acquired by any space-borne instrument (see Sect. 2.4 for uncertainties). Reported at 1 AU from the Sun, this value is 1361 W m^−2^. Integrating over a spherical shell at 1 AU gives a total of 3.828 × 10^26^ W radiatively propagating outward from the Sun. With no intervening energy-loss sources between the Sun and 1 AU, this is the total power emitted by the photosphere over its nearly spherical surface, which has radius 695,700 km (Mamajek, Prša, Torres, et al. 2015) for a surface flux of 6.293 × 10^7^ W m^−2^. That flux, if emanating from a blackbody source, would correspond to a photospheric bolometric temperature of 5772.0 ± 0.8 K (Prša et al. 2016), classifying the Sun as a G2 yellow-dwarf star.

Now evolving on the main sequence, the Sun formed approximately 4.567 billion years ago and currently has a mass of 1.9885 × 10^30^ kg (Mamajek et al. 2015) with a photospheric mass fraction of 74.91% hydrogen and 23.77% helium (Lodders 2003). Gravity due to the Sun’s mass holds the star together, with underlying layers deeper in the solar interior supporting the mass of all overlying layers via increased pressures at greater depths. As the pressures increase toward the Sun’s core, the densities and temperatures increase such that near the core the density reaches 160,000 kg m^−3^ and the temperature is roughly 15.7 million degrees K. At these high pressures and temperatures, hydrogen is fused into helium. To create the Sun’s emitted 3.828 × 10^26^ W, the core must burn 5.889 × 10^11^ kg of hydrogen per second. This energy propagates outward via radiation and convection. With the extremely high internal solar densities and thus short mean-free photon paths, it takes 10^5^ to 10^7^ years for that energy to reach the Sun’s surface (Vardavas and Taylor 2007; Eddy 2009), from which it can then radiate outward into 4π steradians relatively unimpeded by other interactions in the interplanetary medium. Photons emitted toward the Earth at a distance of ~ 1 AU will reach it in another 8 min after the million-year journey of multiple radiative scatterings, absorptions, and reemissions since the time of the nuclear reactions that provided the energy the TSI carries outward.

### Spectral solar irradiance: causes and effects of irradiance variability vary spectrally

**Fig. 2:**
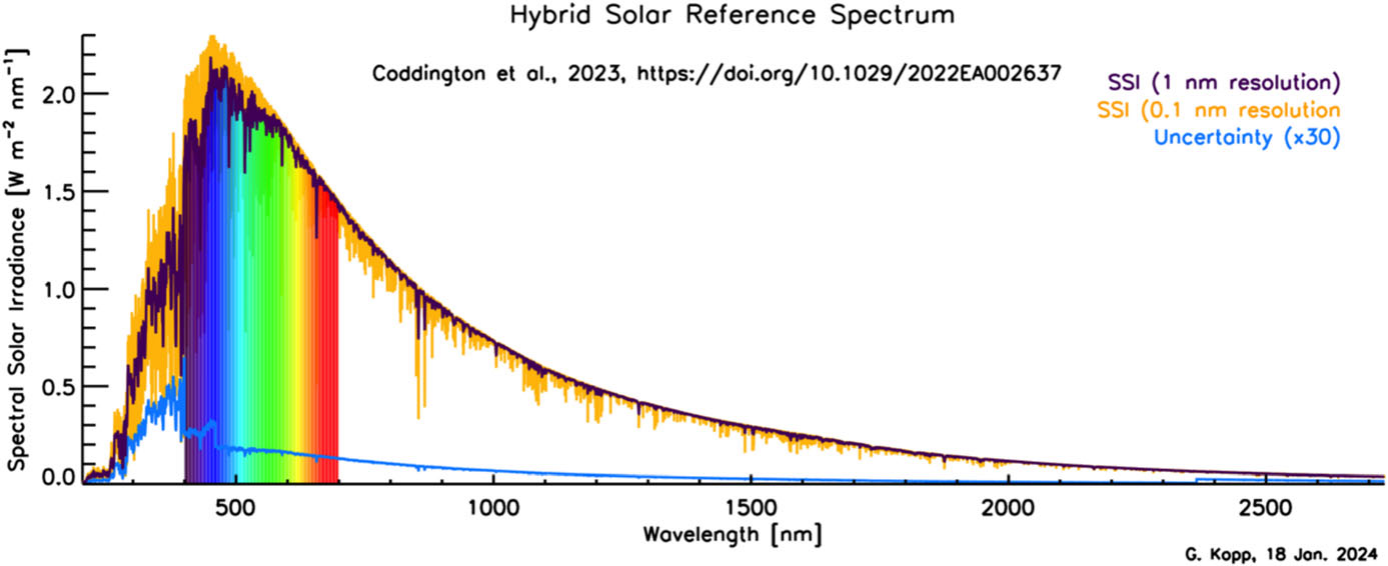
Hybrid solar reference spectrum. This high-resolution spectrum representative of solar minimum combines high-spectral-resolution models giving fine spectral detail with lower-resolution SSI measurements normalizing to the correct scale

Although the Sun has a photospheric bolometric temperature of 5772 K, the thermal profile of the solar atmosphere varies, causing different wavelengths, originating from different solar altitudes, to represent the gas temperatures from where they were emitted. Absorption and emission lines from constituents in the solar atmosphere cause spectral spikes in the Sun’s emission that are often not in thermal equilibrium with the surrounding gas, resulting in a spectral irradiance that is not a blackbody. A high-resolution solar reference spectrum, described in Sect. 2.5.1.7, is shown in Fig. 2.

As with the TSI, the spectral solar irradiance (SSI) varies with time and does so non-uniformly across the spectrum. The cause of irradiance variability for both the TSI and SSI on timescales from days to the solar cycle is predominantly solar-surface magnetic activity, particularly that due to sunspots and faculae, while on timescales of minutes to hours, non-magnetic effects, such as convection, granulation, and solar oscillations, cause variability. Solar-irradiance variability on these and longer timescales is discussed in Sect. 4. Here we merely mention that different solar-activity types cause solar-irradiance variability, and each has unique spectral emissions characteristic of the solar-atmospheric temperatures associated with it. (See, for example, Fontenla and Harder 2005, who use early spectrally continuous UV-to-NIR spectral-irradiance measurements to determine the spectral signatures of seven solar-activity types as a function of extent and position on the solar disk.)

Additionally, different incoming wavelengths are absorbed in the Earth’s atmosphere by various molecular constituents and layers (Gray et al*.* 2010; Lean and Woods 2010; Matthes 2011; and Ermoli et al*.* 2013). With both the solar-irradiance variability and the Earth’s atmospheric and surface-temperature responses being wavelength dependent, measuring the SSI as well as the TSI is necessary to understand the influences of solar variability on climate.

Given this climate relevance, it is important to understand the sources of solar radiant power and the causes of its variability. Doing so requires accurate, long-term, high-precision, space-borne, total- and spectral-solar-irradiance measurements. The spectral solar irradiances described in this review cover the primary energy output spectrum of the Sun spanning from roughly 200 to 2400 nm, acquiring approximately 96% of the TSI (i.e., spectrally integrating to 1309 W m^−2^ vs. the TSI’s value of 1361 W m^−2^, with most of the TSI’s additional ~ 4% being in the infrared).

## Solar-irradiance measurements

### Pre-spacecraft measurements

The Sun's total irradiance was historically referred to as the “solar constant.” Many Earth-climate authors continue to use this misnomer because the Sun, fortunately, is relatively stable with time compared to other present-day climate drivers. Because the Sun is so stable, extremely precise measurements are needed to detect changes in the solar irradiance. To achieve the precision needed, present-day measurements are obtained from space, as temporal changes in the absorption and scattering in the Earth’s atmosphere are much greater than the intrinsic solar variability and prohibit sufficiently accurate and precise measurements from the ground. Prior to space-borne measurements, historical solar-irradiance measurements were attempted from ground-based sites; however, atmospheric variability and corrections largely precluded conclusive measurements of solar variability, and the term “solar constant” could not be refuted.

**Fig. 3:**
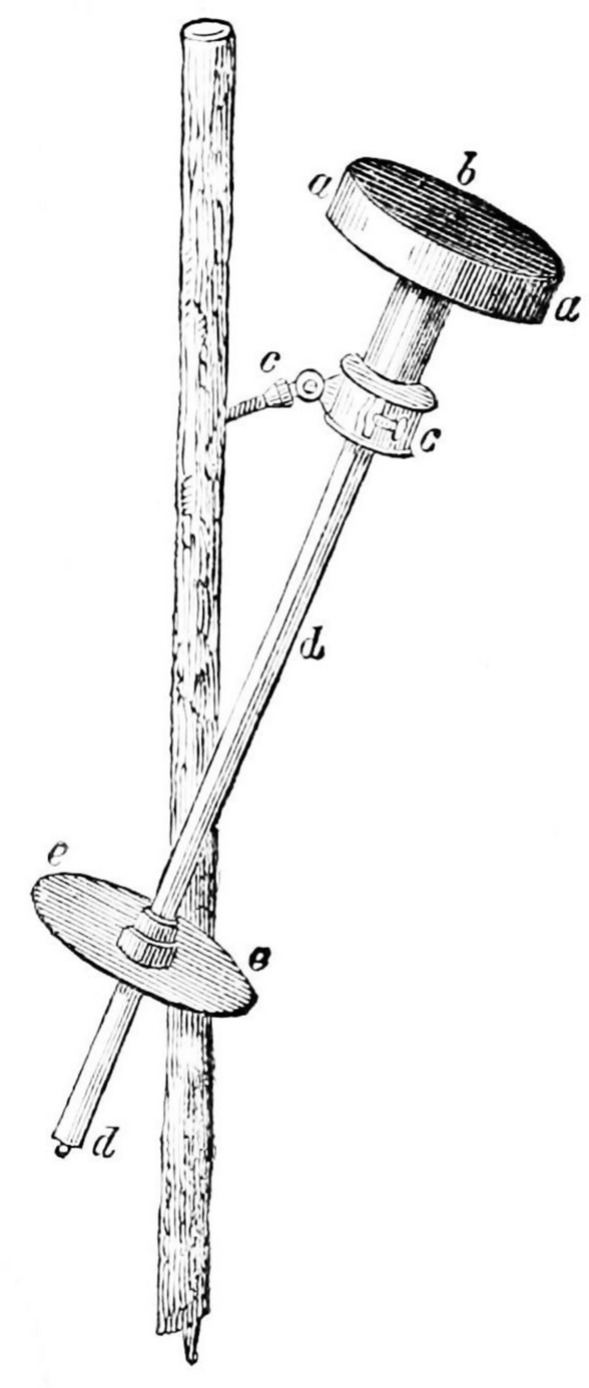
Early solar irradiance instrument. Pouillet’s instrument for measuring the TSI by heating a known mass of water in a cylindrical container (**a**) by sunlight incident on the absorptive surface (**b**). From the measured rate of temperature change measured by the shielded thermometer (**d**) and the known heat capacity of water, the incident solar power can be computed, and the surface (**b**) gives the area over which it is collected. A similarly sized plate (**e**) enables alignment normal to the incident sunlight. (from Young 1880)

French physicist Claude Pouillet (1790–1868) and British astronomer John Herschel (1792–1871) were two of the first scientists to attempt to quantify the Sun’s radiant power. Their independent measurements relied on similar principles of heating a known mass of water contained in a dark-surfaced flask using incident sunlight. The rate of temperature change gives the incident power based on the known heat capacity of water and the absorptivity of the flask’s Sun-facing surface. The ratio of that power to the flask’s collecting area gives a measure of the solar irradiance. A drawing of Pouillet’s instrument is shown in Fig. 3. His and Herschel’s reported values for the TSI were lower than the currently accepted value of 1361 W m^−2^, with Pouillet estimating a value of 1227 W m^−2^ after corrections for atmospheric losses (although he expressed this value as 17.6 kcal m^−2^ min^−1^; Young 1880).

The difficulties in obtaining these earliest values are not surprising, as, even under clear-sky conditions, spectrally dependent absorption and scattering by water vapor, dust, aerosols, and other atmospheric particles in the Earth's atmosphere limit the energy incident at the top of the atmosphere from reaching the Earth’s surface, where Pouillet and Herschel acquired their measurements. Successive measurements over the next half-century acquired at different altitudes by various solar-observing expeditions helped quantify the fraction of the incident sunlight absorbed by the Earth's atmosphere. These corrections emphasized the need for measurements from the highest feasible altitudes combined with appropriate atmospheric-transmission corrections.

In 1881, American scientist Samuel Langley (1834–1906) acquired the most meticulous measurements of the TSI from that era. He performed multispectral measurements from different altitudes, determining the variation of atmospheric absorption as a function of wavelength and enabling atmospheric-transmission corrections. Using data from Mt. Whitney (elevation 4420 m) in the southern California Sierra Nevada mountains and a more modern bolometer-based instrument that relied on the sensitive variation of electrical resistivity in metals due to temperature changes, Langley calculated a TSI value of 2903 W m^−2^. This value is more than a factor of two larger than the currently accepted value. Despite the meticulousness and thoroughness of his measurements, Langley’s erroneously high TSI value was evidently caused by data-analysis errors.

Langley’s assistant, Charles Abbot (1872–1973), continued his work, acquiring ground-based solar-irradiance measurements from many worldwide locations from 1902 to 1962 that were maintained by the Astrophysical Observatory of the Smithsonian Institution. Abbot was meticulous in both the measurements he and his team continued to acquire as well as the wavelength-dependent atmospheric corrections he applied for effects of air mass, water vapor, temperature, and circumsolar scatter. [See, as evidence, his 78 pages of results spanning 1923 to 1939 using measurements from ground stations at Montezuma in Chile, Mount St. Katherine in Egypt, and Table Mountain in California (Abbot et al. 1942).] Abbot reported “1.946 [calories cm^−2^ min^−1^] as the best result of Smithsonian solar-constant determinations” (Abbot 1965), which corresponds to 1357 W m^−2^, putting it in surprisingly good agreement with today’s values given the sizeable corrections needed for the ground-based measurements. Abbot also recognized that the “solar constant” varied, reporting the following results of the Sun’s radiant power: decreases due to the passage of large sunspots across the solar disk over a few days of ~ 0.1%; cyclic changes during the full 22-year Hale-cycle of ~ 0.1%; and a general increase in brightness associated with higher numbers of sunspots (Abbot 1958). He further linked these solar variations to regional weather and magnetic storms at the Earth (Abbot 1958, 1966). Despite publishing results showing what he was certain was actual solar variability and not an erroneous artifact of the observations and the many corrections applied, Abbot continued to refer to the Sun’s brightness as the “solar constant.” Although his solar-variability results were disputed by other researchers at the time, as detailed in a lengthy review of the Smithsonian solar constant program by Hoyt (1979), they are consistent with present-day measurements.

Abbot’s ground-based measurements were superseded in the latter half of the twentieth century by balloon-, rocket-, and space-flight instruments. Labs and Neckel (1971) summarized the values reported from 13 different experiments over the 3-year period from 1967 to 1970. These values ranged from 1338 to 1458 W m^−2^, from which they gave a “most probable” value of 1360 W m^−2^ (1.95 cal cm^−2^ min^−1^). They did admit that variations of the “solar constant” could be comparable to their stated uncertainty of ± 1%.

The first TSI measurements by absolute radiometers not dominated by atmospheric attenuation were those by Willson’s new active cavity radiometers (ACRs) flown on high-altitude balloons in 1968 and 1969, the latter of which yielded a value of 1369 W m^−2^ with a 0.6% (8 W m^−2^) quoted uncertainty (Willson 1979). A > 100-km sounding-rocket flight in 1976 with an improved design, the ACR IV, gave a similar value of 1368 W m^−2^ ± 0.5%.

The World Radiometric Reference (WRR) was established in 1977 as a “conventional” (as opposed to “SI”) primary standard for the TSI, replacing the prior International Pyrheliometric Scale of 1956 (IPS56), which had a 2.2% (30 W m^−2^) error in absolute scale. Maintained at the co-located Physikalisch-Meteorologisches Observatorium Davos (PMOD) and World Radiation Center (WRC), the present-day WRR consists of six cavity radiometers that measure solar irradiance from the ground (Finsterle et al. 2008). The ensemble average of the measurements from this group gives the WRR value of the TSI. Since these radiometers observe through the Earth’s atmosphere, they are designed to have nearly identical viewing geometries, so they each include a similarly small portion of the sky surrounding the Sun, allowing this circumsolar scatter to be included equally in the simultaneously observing, co-located instruments. On-site group intercomparisons of the WRR to other solar-observing instruments are performed every 5 years via International Pyrheliometer Comparisons to disseminate the WRR value worldwide. These intercomparisons continue to the present era, although the benefit at this point is more for inter-calibrating ground-based solar instruments than fully calibrating spaceflight TSI-instruments, as a more modern and flight-like facility based on a reference electrical-substitution radiometer now exists and provides a more direct connection to the International System of Units (SI) (Kopp et al. 2007). Fehlmann et al. (2012) provide a comparison between the WRR and the SI-traceable radiometric scale, finding that the WRR reads 0.34 ± 0.18% (4.6 W m^−2^) high compared to the (absolute) SI-scale. (See the effects of this in Sect. 2.4.12.)

The pre-spaceflight attempts to measure the TSI were limited by the instrumentation and short-duration high-altitude platforms available. It was debatable whether the reported variations between measurements were indicative of variability in the Sun’s radiative output or due to the much larger effects of Earth’s highly variable atmospheric transmission. The TSI, as shown by subsequent long-duration space-borne measurements, generally varies by less than the uncertainties of these early ground-, balloon-, and rocket-based measurements.

What, then, are the needed measurement requirements for discerning climate sensitivity to solar variability?

### Measurement requirements

Determining required solar-irradiance-measurement stabilities and accuracies relies on estimates of the magnitude of long-term solar variability as well as the Earth-climate sensitivity to solar variations. For instance, extremely high climate sensitivity to solar-irradiance changes would require very precise monitoring of small changes in the Sun’s output, as even those could have significant effects; conversely, if solar variations over decadal scales were large and climate sensitivity to solar changes were relatively small compared to other influences, then solar-measurement requirements could be less stringent.

Earth’s climate can be influenced by solar changes over years to centuries and thus motivates the need for a stable measurement record of similar duration. There are two fundamental approaches to detecting long-term variability in the Sun’s radiant output:

1. **Absolute Accuracy**. If measurements can be acquired with high absolute accuracy (i.e., low standard uncertainty), long-term solar variability can be detected via two non-overlapping instruments if their combined measurement uncertainties are less than the irradiance differences of their two temporally separated measurements.
2. **Measurement Continuity and Stability**. Solar-irradiance trends can be detected by a continuous series of measurements of overlapping instruments if the long-term measurement precision (i.e., stability) is less than the rate of variation in the irradiance itself.

Detecting irradiance variations on solar-cycle to century timescales are the most relevant for setting measurement requirements, as these have direct climate impacts. These variations are ~ 0.1% over a solar cycle and likely  < 0.1% over century timescales for the TSI, as described in Sect. 4. The more stringent value, measuring variations of  < 0.1% over century timescales, drives TSI measurement trend-detection requirements of  < 0.001% year^−1^ (see assessment by Kopp 2014 with supporting rationale given here in Sect. 4.5), necessitating instrument stabilities of similar or better magnitudes as well as continuous measurements. Maintaining measurement continuity over long time-durations, however, is at ever-increasing risk of loss due to data gaps between instruments, and those risks increase with measurement-record duration. Trend detection on long timescales thus favors a measurement approach fundamentally based on absolute accuracy.

Absolute accuracy has increasing importance compared to measurement continuity and stability as the measurement record lengthens. Determining the duration at which a transition from continuity and stability to accuracy is appropriate depends on the measurement stabilities and accuracies achievable. The linear solar trend of 0.001% year^−1^ mentioned above illustrates this point. Such a trend would be marginally detectable at the 1-*σ* level via instruments having comparable 0.001% year^−1^ stability uncertainty regardless of the measurement duration. However, after 4 decades (which is less than the duration of the current space-borne TSI record), this trend would cause a change in the Sun’s radiative output of 0.04%. This change should be detectable at nearly the 3-*σ* level via one measurement at the beginning of the record and one at the end, assuming each achieves 0.01% absolute accuracy.

The current TSI-measurement approach relies on both absolute accuracy and measurement continuity from stable instruments. The TSI record’s measurement requirements, as given by Ohring (2007) and Kopp (2014), are summarized in Table 2. Interestingly, two additional and independent climate-sensitivity means of deriving TSI measurement requirements reach very similar conclusions, as described by Stephens et al*.* (2013).

**Table 2:** Solar-irradiance measurement requirements for climate studies

Measurement parameter	Requirement
Absolute Accuracy	< 0.01%
Stability	< 0.001% year^−1^

The SSI variability on these timescales is less well known, since the measurements span a shorter duration and currently lack the stability needed to definitively ascertain changes over solar-cycle and longer timescales. Similarly, spectral dependencies of Earth-climate sensitivity continue to be determined. Thus, the needed SSI-measurement accuracy and stability requirements are less rigorously defined than for the TSI. In their stead, since the SSI and TSI are correlated (at least on shorter timescales; see Sect. 4.2) over much of the visible and NIR spectral range, which contributes most of the energy in the TSI powering the Earth-climate system, the TSI measurement requirements in Table 2 may be expected to provide a reasonable estimate of the SSI measurement requirements for long-term climate studies as well.

**Fig. 4:**
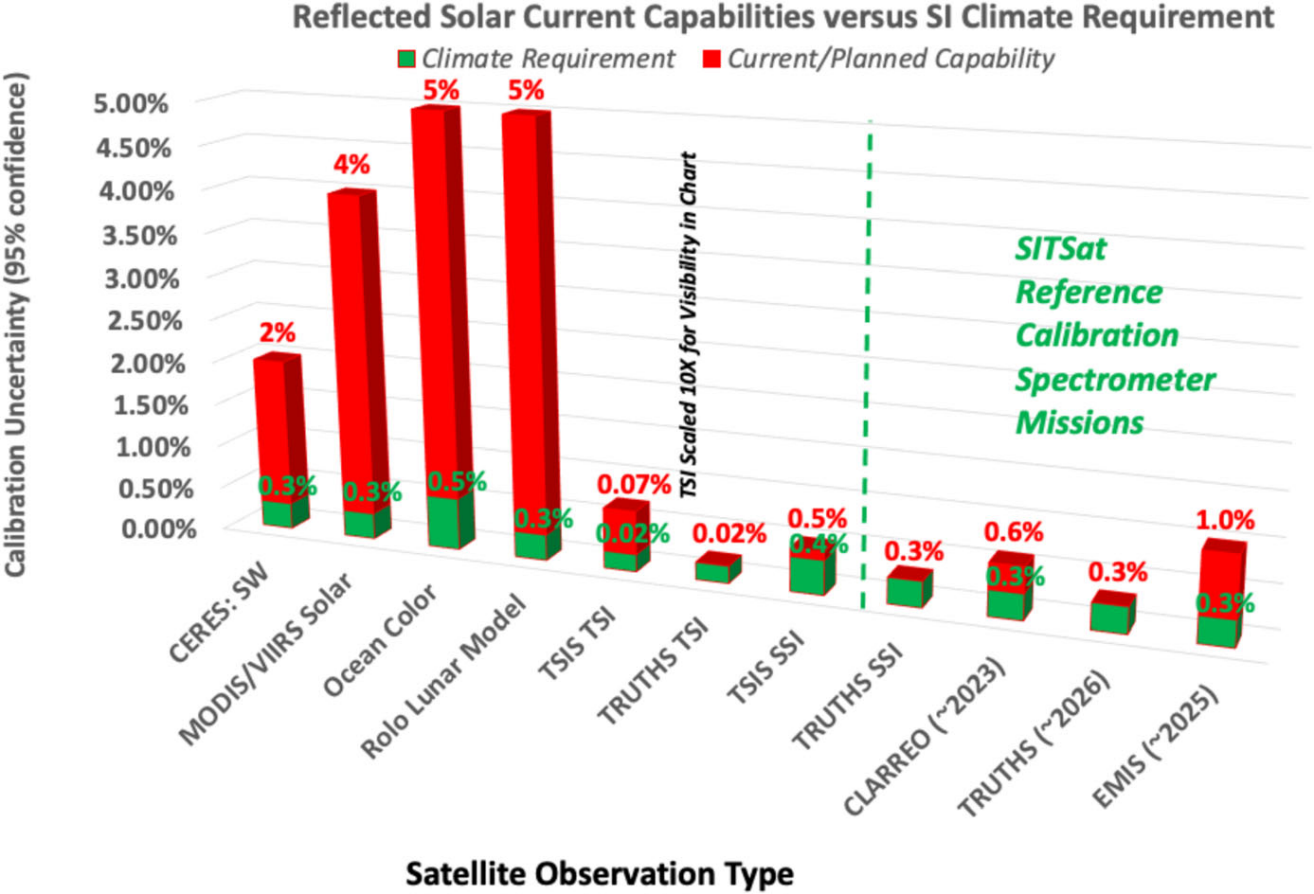
Earth-climate measurement requirements in the reflected-solar spectral region: Climate-required measurement accuracies and current capabilities of Earth-incoming and -outgoing radiation across the solar spectrum are summarized in this plot from Fox et al. (2022). (Note that the requirements shown state lower uncertainties than imposed on many spacecraft programs, as these are the climate-driven requirements as opposed to the requirements levied on instruments in order to programmatically claim mission success.)

The climate-required measurement accuracies of incoming and solar-reflected outgoing radiation from the Earth are compared to current capabilities in Fig. 4. TSI measurements are nearly achieving these climate-driven levels of accuracy, and newer SSI instruments are approaching the stability levels to discern solar-cycle variabilities. The instrument designs enabling these capabilities are described in Sect. 2.3–2.5

### Spaceflight instrument designs

Detecting solar variabilities of ~ 0.1% on 11-year solar-cycle timescales, such as Abbot (1958) claimed, relies not only on being above the Earth’s atmosphere to acquire sufficiently accurate and stable measurements but also on spacecraft instruments having stability uncertainties much lower than the solar variability to be detected. Since instruments degrade over time when exposed to unfiltered top-of-atmosphere solar radiation, internal means of tracking changes in instrument sensitivity are needed as well as radiometric measurements having good absolute accuracy.

Total irradiance is a measure of net radiant power per area, requiring measurements of each with high accuracy and stability. Spectral irradiance additionally requires knowledge of the spectral bandpass over which that radiant power is measured. All spaceflight TSI instruments have relied on the stability and accuracy of bolometric radiometers as their core detectors of radiant solar power. SSI instruments generally use functionally similar radiometers designed for much lower power measurements and/or complementary high-sensitivity solid-state detectors. Both TSI and SSI instruments rely on apertures to define the area over which the sunlight is collected. The latter also require some means of spectral selection. Redundant channels using different solar-exposure duty cycles help determine exposure-dependent instrument degradation and allow for its correction in data processing. These instrument components and methodologies are discussed below.

#### Electrical-substitution radiometers

While solid-state detectors such as photodiodes and charge-coupled devices have extremely high precision and sensitivity, they lack the long-term stabilities, the broad spectral ranges, and the uniform spectral sensitivity needed for TSI measurements, which must span wavelengths from X-rays to the far-infrared with a single detector. The standard for both space- and laboratory-based radiometry is the bolometer, and all spaceflight TSI instruments are bolometer-based. Their stability makes them the basis for most SSI measurements as well, although the best of those instruments often incorporate a bolometer and a solid-state detector in a hybrid system to benefit from the long-term stability of the bolometer and the measurement speed and low noise of the solid-state devices.

Space-borne solar-irradiance bolometer systems measure sunlight with a radiometer having a light-absorbing dark surface or cavity, which turns the absorbed incident radiant power into thermal power. Much as Pouillet’s instrument of 1832 operated (see Fig. 3), the accurate measurement of this thermal power is a direct indicator of the sunlight power absorbed. Corrections are applied for light-absorption efficiencies; loss or gain of light due to scatter or diffraction; instrument thermal fluctuations and sensitivities; internal parasitic radiant, thermal, and electrical losses; and many other subtle effects. While laboratory radiometers often operate at cryogenic temperatures to reduce their internal thermal background, all space-based solar-irradiance instruments to date have operated at near-ambient temperatures, as the mass, power, volume, lifetime, and complexities of cryogenic-cooling systems add expense that is largely unnecessary.

The most accurate spaceflight solar-irradiance instruments rely on electrical-substitution radiometers (ESRs), in which the instrument’s radiometer is thermally controlled to a stable temperature slightly above ambient via the application of electrically applied resistive-heater power. An active solar-monitoring ESR is heated electrically to match a (desirably stable) reference instrument temperature, which is often an identical but non-solar-viewing ESR in a similar thermal environment, so that common-mode thermal fluctuations reduce external influences. As a shutter binarily modulates sunlight incident on the active (solar-viewing) ESR, the electrical heater power applied to that ESR must be modulated to maintain constant temperature despite the application and blockage of the incident radiant sunlight power. The electrical-heater power needed to maintain the active ESR at constant temperature will decrease when sunlight illuminates the ESR’s cavity interior and increase when that radiant power is blocked. The modulations of the applied heater-power in conjunction with calibrated light-absorption efficiencies accurately establish the entering radiant solar power. The electrically applied power can be measured with uncertainties of ~ 10^–6^, helping achieve high radiant-power measurement accuracies.

A heat sink having much higher thermal capacity than the ESR helps maintain the radiometer’s thermal stability via a weak thermal link. This heat sink can be either passive or actively thermally controlled. The sensitivity of the ESR increases inversely with its mass, thermal specific heat, and thermal conductivity to this heat sink, while the thermal-response time varies directly with mass and thermal specific heat and inversely with thermal conductivity. The ESR design is therefore tuned to the expected radiant-power levels, with the lower-power radiometers needed for SSI instruments fundamentally being miniature versions of those used in TSI instruments. Most TSI-instrument ESRs measure power levels in the 30- to 70-mW range, depending on the size of the aperture defining the area over which sunlight is collected. Typical SSI-instrument ESRs are designed for power levels of  < 2 mW depending on their aperture size and spectral resolution. The higher-power ESRs for TSI measurements generally utilize a conical-cavity geometry having the conical opening pointed toward the Sun such that entering light scattered inside the black interior of the cone preferentially scatters deeper toward the conical apex to be absorbed on subsequent incidences, giving high absorption efficiency. This was facilitated via specular black paint on many early instruments.

#### Apertures

A precision-machined aperture of accurately known dimensions determines the area over which the incident radiant sunlight is collected. (This is analogous to the area of the black-painted disk in Pouillet’s instrument in Fig. 3.) The ratio of the radiant power measured by the detector to the area over which it is collected gives the TSI and is typically expressed in units of W m^−2^. The SSI reports this value per unit wavelength appropriate for the spectral bandpass used. Traditionally, these precision apertures have been machined from metals with sharp knife-edges and then calibrated for geometric area. The National Institute of Standards and Technology (NIST) has demonstrated uncertainties in such measurements of  ~ 2.5 × 10^–5^ for some of the larger apertures used in TSI instruments. More recently, photolithographic techniques have provided precision apertures from silicon that have similar uncertainties from smaller-area apertures used in SSI or compact TSI instruments. Corrections must be applied for the aperture’s thermal expansion, ground-to-space pressure expansion, diffraction, and scatter (see Kopp et al. 2005a, b). As an example, aluminum apertures, having a coefficient of thermal expansion (CTE) of ~ 2 × 10^–5^, machine to good knife edges but have thermal areal corrections that are about 0.0004% C^−1^. Pressure corrections between ground-based calibrations and use in vacuum are smaller, at  < 10^–6^. Diffraction from ideal knife-edge apertures causes loss of some incident light that depends on aperture size, distance between the aperture and the detector, and wavelength. These values can be measured (Harber et al. 2006) and calculated, the latter giving uncertainties in a typical ~ 5 × 10^–4^ TSI-instrument diffraction correction of  < 1.8% (Shirley et al., 2002; also see numerical updates by Shirley 2016; Rubin et al. 2018). Scatter from aperture edges is highly dependent on machining and must be empirically measured. TSI instruments use circular apertures, since their detectors similarly have circular entrances, while SSI instruments generally have rectangular-slit apertures to feed light into their spectrometers.

#### Spectral-selection methods

In addition to the radiant-power measurement and collection-area knowledge needed for TSI instruments, SSI instruments require a means of accurate spectral selection. Long-term on-orbit spectral-irradiance measurements utilize at least one of three standard methods, each having specific advantages and limitations, as described below.

##### Filters

Spectral filters provide fixed-bandpass throughput. They are simple and relatively inexpensive compared to other spectral-selection methods and are often paired with photodiode detectors for compact, spectrally static, low-mass irradiance-monitoring systems. They are particularly used in the UV and soft X-ray, where other methods have low efficiencies and can require complex grazing-incidence optical-dispersion systems. At these shorter wavelengths, the solar variability is high, so the filter and detector stability on long timescales is less of a concern than in the visible and NIR.

Filters are susceptible to pinhole and out-of-band leakage and must be well characterized via pre-flight calibrations. Contamination can degrade filter response in both overall efficiency and spectral bandpass with time, particularly when exposed to high-energy photons from the Sun, and there can be spectral throughput changes due to gradual blending between layers in multi-layer thin-film coating designs. In the UV and soft X-ray, any long-wavelength out-of-band leakage can easily dwarf the low signals at the intended short wavelengths.

Spectrally tunable filters have not yet been used for space-borne solar-irradiance measurements. While spectral variability would offer useful capabilities, methods of providing that variability generally reduce the throughput knowledge and stability, increasing the resulting spectral-irradiance measurement uncertainties.

##### Prisms

Prisms spectrally disperse light in transmission via the wavelength dependence of the prism material’s index of refraction. That index generally increases at shorter wavelengths, giving higher dispersion toward the UV, where the Sun is relatively more variable both spectrally and temporally. With continual spectral variation in index of refraction, angular dispersion is unique for each wavelength, and broad-spectral-range instruments can be designed with appropriate prism design and material selection. These systems require a long (or folded) optical path to allow good spectral separation and either need multiple detectors, such as a 1-D array, or a means of moving either a single-element detector or the prism itself to scan a desired spectral range. Throughput efficiency must be well characterized in pre-flight calibrations and favors non-folded (but longer) optical paths. Changes due to contamination or degradation of either the prism surface or internal transmission efficiency must be accounted for to achieve good stabilities. These effects can change both efficiency and instrument line shape (aka spectral resolution).

##### Gratings

Gratings provide spectral dispersion via diffraction, whereby different wavelengths constructively or destructively interfere at well-defined exit angles from the grating surface. These elements can be ruled or holographic with efficiencies customized via effective blaze angles. Dispersion is well defined by the incidence and exit angles and the grating’s line spacing. Spectral range is broad but overlap of wavelengths can occur due to high-order diffraction from shorter wavelengths exiting at the same angles as low-order diffraction from longer wavelengths. Occurring at integer ratios of wavelengths, such overlap is well known but requires spectral sorting of the different orders. Contamination of the grating surface may change efficiency and cause scatter but will not change spectral dispersion, which is controlled by the grating’s line spacing. Often, double-pass or double-monochromator designs are used to reduce scatter in large-dynamic-range grating-based instruments. As with prisms, spectrometers using gratings require long optical paths and either a 1-D spectral-dimension detector array or a means of scanning either a single-element detector or the grating. Grating systems also require complex optics for collimation onto the grating and focusing onto the detector. These are generally the most complex and expensive of spectral-selection methods for irradiance measurements.

#### Instrument degradation tracking methodologies

Being spaceflight detectors exposed to sunlight almost continually during operations, solar-irradiance instruments suffer on-orbit degradation due the incident ultraviolet and X-ray solar radiation. To provide long-term spaceflight-measurement stability, this degradation must be minimized and/or tracked and corrected.

The most common degradation-tracking method utilizes redundant optical channels and/or interchangeable components in the optical path to correct for solar-exposure-dependent sensitivity variations that are mainly due to brightening of the absorptive surfaces or, in the case of SSI instruments, optical-efficiency changes. During spaceflight operations, a primary radiometer (and optical train in the case of SSI instruments) is used for most solar measurements. Variations with time in the differences between the irradiance values measured infrequently but simultaneously by this primary and a much-lesser-used (and therefore presumed more stable) alternate channel allow for tracking of the degradation in the primary due to solar exposure. SSI instruments have the additional complication that they must measure and account for a wavelength dependence to their degradation corrections. The magnitude of on-orbit degradations and how well they can be tracked determine instrument measurement-stability uncertainties.

While corrections based on the tracked instrument degradation are applied in released solar-irradiance data, the corrections contribute additional uncertainties, with larger applied degradation corrections likely corresponding to larger uncertainties in long-term instrument stability. Future instrument designs described in Sect. 5.2 are intended to help meet both the solar-irradiance measurement-accuracy and -stability requirements that should enable more definitive detections of solar variability on long-term timescales.

### Space-borne TSI measurements

**Fig. 5:**
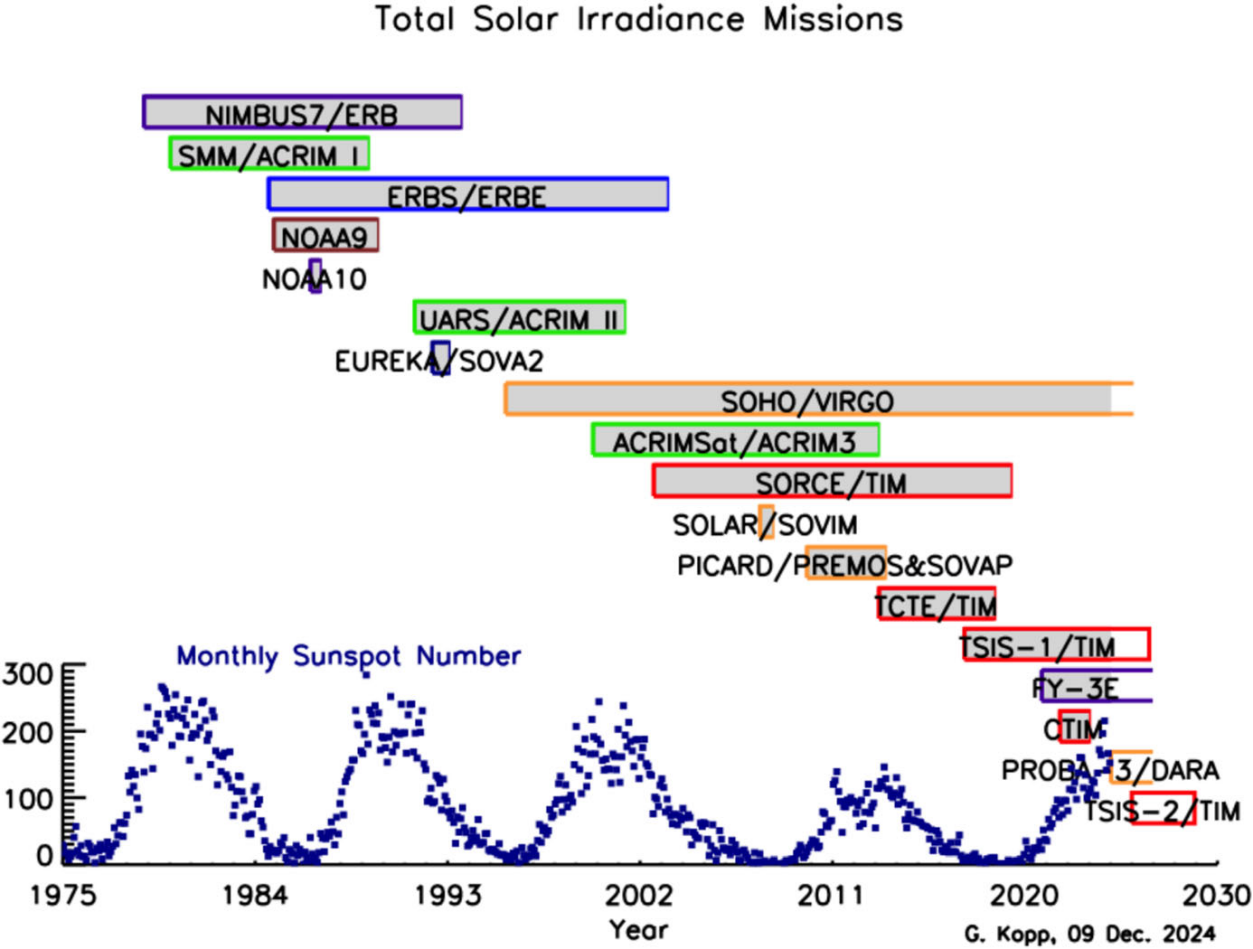
TSI missions. Nearly 20 TSI missions with publicly available data have contributed to the uninterrupted 47-year data record. Gray shading indicates times before the present

**Fig. 6:**
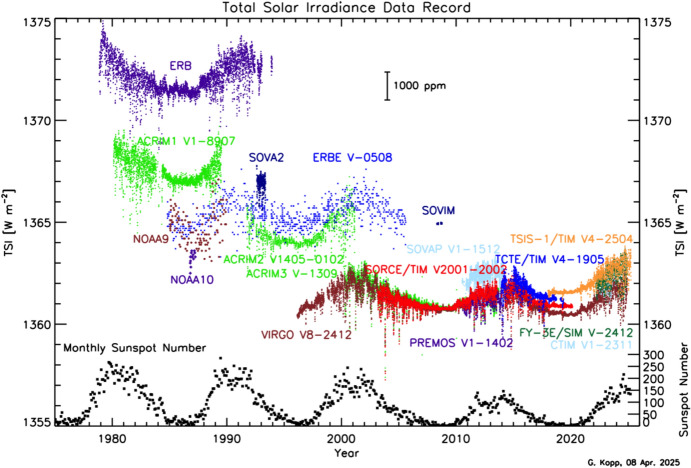
Space-borne TSI Record. The TSI has been measured from space via an uninterrupted series of overlapping instruments since 1978. Scale differences between instruments are due to calibration differences. Note that TSI fluctuations of ~ 0.1% are in phase with solar-surface magnetic activity over the 11-year solar cycle, as indicated by sunspot numbers (black). (updated regularly at http://spot.colorado.edu/~koppg/TSI)

Space-based TSI measurements began with the National Oceanic and Atmospheric Administration (NOAA) and National Aeronautics and Space Administration (NASA) Nimbus-7 in 1978 and have been uninterrupted since thanks to a series of overlapping flight instruments from the NASA, NOAA, European Space Agency (ESA), Centre National d'Etudes Spatiales (CNES), and, more recently, the China National Space Administration (CNSA). Table 3 lists the operational time ranges of each of the several instruments that have contributed to the current solar-irradiance data-record, with a corresponding graphic in Fig. 5. Figure 6 shows the data from each. Scale differences (i.e., vertical offsets) between these instruments are due to instrument absolute-scale calibration errors. In an assessment of spaceflight TSI instruments, Kopp (2014) shows that the earlier instruments failed to demonstrate the climate-driven 0.001% year^−1^ measurement stabilities for long-term detection of TSI variability. Improvements in ground-based calibrations and intrinsic instrument stability, such as achieved by newer instruments using robust metal or even more stable carbon-nanotube absorptive radiometer surfaces, now help meet these TSI-measurement accuracy and stability requirements.

**Table 3:**
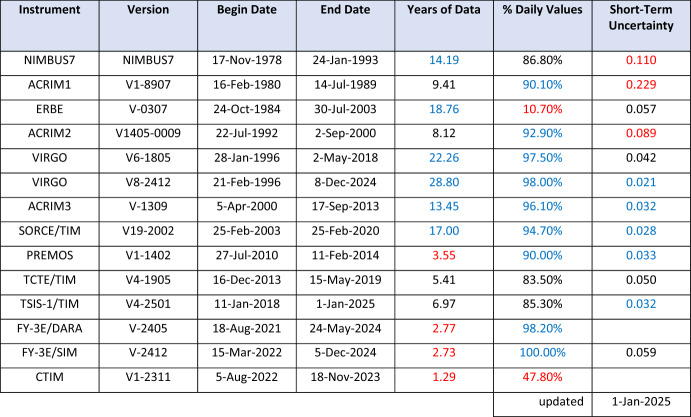
Space-borne TSI instruments and data ranges

All NASA TSI instruments and missions were funded by the organization’s Earth Science Division, since the primary purpose of these instruments is Earth-climate science as opposed to solar studies. The measurement cadences, accuracies, stabilities, and durations of these and other missions are thus driven by long-term observations of incoming Earth radiation for climate studies as opposed to focusing on shorter-term solar phenomena, such as solar flares, oscillations, convection, and granulation. Almost all are based in low-Earth orbit (LEO) and have times where the Sun is occulted by the Earth for portions of their ~ 95-min orbital periods and/or seasonally due to orbital beta-angle variations.

Below is a chronological summary of the primary long-duration TSI missions and instruments providing publicly available data.

#### Nimbus-7/ERB

**Fig. 7:**
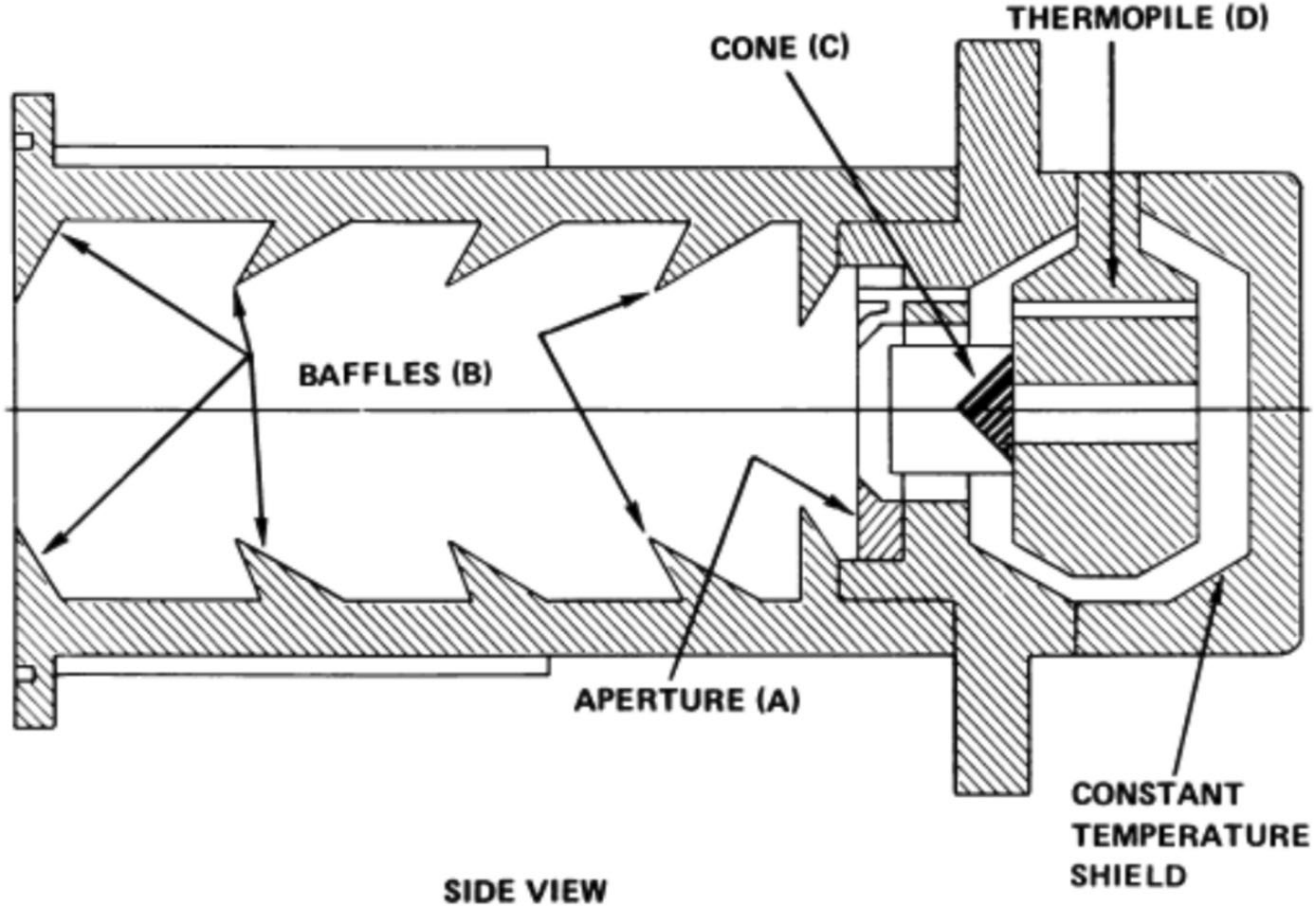
ERB cross-section. The single-channel ERB used an inverted-cone geometry inside a cylindrical cavity to absorb incident sunlight, which, in this figure, enters from the left. The precision 0.5-cm^2^ aperture defining the area over which light is collected is immediately in front of the cavity, as on most early instruments, while upstream baffles define the field of view. Image reproduced with permission from Hickey et al. (1988), copyright by Springer

**Fig. 8:**
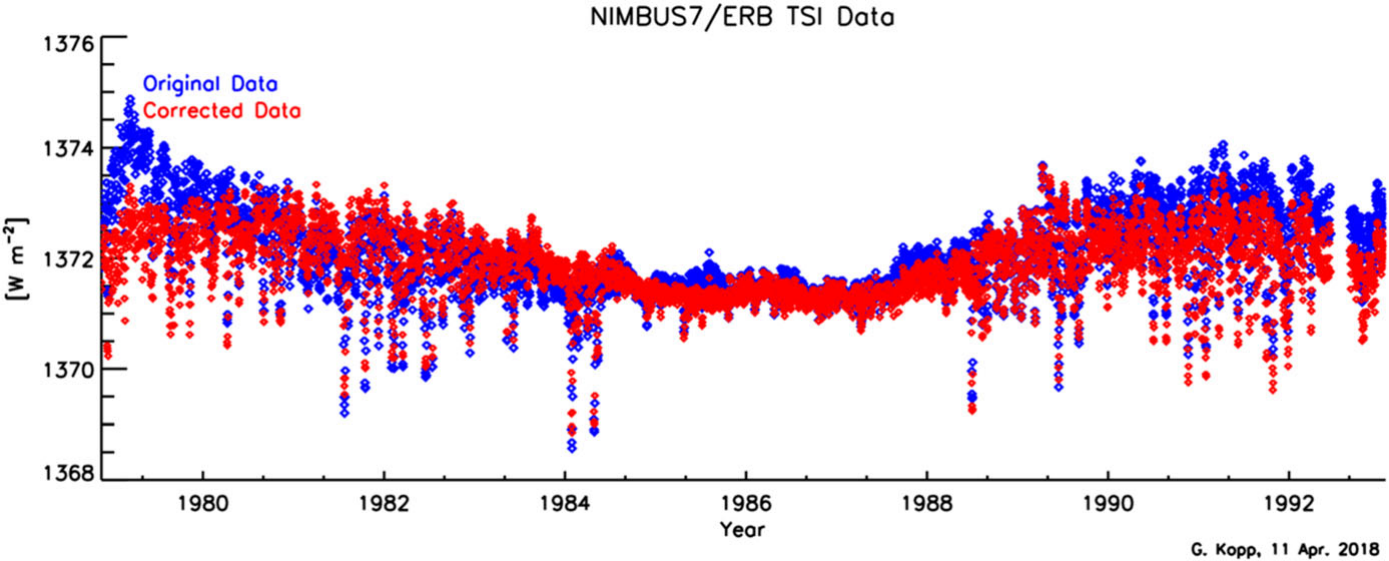
ERB TSI Data. The daily TSI values are plotted from the ERB, which initiated the space-borne TSI record. Both the uncorrected data, as provided by the original instrument team, and C. Fröhlich’s estimated corrected data are shown for this instrument that, being a single-channel radiometer, was unable to internally monitor its on-orbit degradation

The Earth Radiation Budget (ERB; Hoyt 1979), launched on the NASA and NOAA meteorological NIMBUS-7 mission in 1978, was the first long-duration spaceflight TSI instrument. It was an improved version of the wire-wound thermopiles used in the Eppley-JPL radiometers. Also referred to as the “Hickey–Frieden” or “HF” instrument, this was a single-channel pyrheliometer consisting of an “inverted” (such that the cone tip instead of the cone mouth faces the Sun) 60°-apex cone inside a cylindrical cavity (Hickey et al. 1988), as shown in Fig. 7. Specular black paint on these surfaces absorbs incident sunlight when it first strikes the inverted cone or reflects it onto the surrounding cylindrical walls, where it is absorbed. Being a single radiometer, the ERB had no internal means of tracking—and thus correcting for—degradation. Via comparisons to proxies and correlations with spacecraft pointing changes, others (Lee et al. 1995; Chapman et al. 1996; Fröhlich and Lean 1997; Fröhlich 2006) have applied corrections to these data, although some are refuted by the original instrument team after their reanalysis of the raw data (Hoyt et al. 1992; Hoyt 2008). Fröhlich argues that, since the ERB used the same cavity geometry and the same specular black paint as the later VIRGO/PMO6 (see Sect. 2.4.4), the VIRGO/PMO6 degradation corrections should be applied to the ERB. Both the uncorrected and corrected data are plotted in Fig. 8.

Initially, the ERB was operated in a 3-days-on/1-day-off mode, and the first of the “on” days was generally discarded because thermal equilibrium had not been achieved. The instrument was continually powered after the first few years of operations, making for more stable and usable measurements.

The ERB was not actively pointed at the Sun. Measurements were acquired when the Sun passed through the instrument’s field of view while the polar-orbiting spacecraft transited the Earth’s southern terminator, giving 1-s-cadence measurements having durations of about three minutes on each 104-min orbit. The measurements were corrected for a cosine pointing response of the radiometer. A shutter was not needed, as the instrument acquired dark measurements while pointed to dark space during much of the remainder of each orbit. Daily averages of measurements on orbits when they were acquired were reported, giving an average of 1376.0 ± 0.73 W m^−2^ from Nov. 1978 to May 1979 (Hickey et al. 1980). Some ERB time series include interpolations for days when no measurements were made. The team relied on the WRR for calibrations. Estimated uncertainties were 0.5%.

The ERB measured short-term decreases in the TSI due to the formation and passage across the solar disk of sunspots, darker and cooler regions on the solar surface, and increases due to faculae. This was much as expected. However, after an initial early increase in signal, the ERB also showed a long-term downward trend in the TSI. This was surprising at the time, as the numbers of sunspots were decreasing nearing the end of that solar cycle, and so the TSI was expected to increase or at least remain at some “solar constant” level. We now know that the TSI varies in-phase with the solar cycle, and thus the observed ERB downward trend was merely following the end of the solar cycle. At the time of these initial measurements, however, the solar-cycle trends were not known (see, for example, Foukal 1981), and most studies focused on short-term, and often individual, active regions rather than the solar cycle. Oster et al. (1982) report “almost complete balance” of sunspot deficits and facular brightenings in the TSI, attributed to active regions angularly redistributing the radiant energy, stating that “11 year [sic] cyclic changes of the total solar irradiance due to activity alone may be close to negligible.” Similarly, Lawrence et al., as late as 1985, suggest TSI decreases due to sunspots are offset by increases due to faculae for net energy balance. A major point made by Schatten et al. (1982) was, “We do not find a variation of our computed irradiance level correlated with the phase of the solar cycle above about ± 0.03%.”

The explanation of the downward trend in the ERB results thus was initially presumed to be a gradual decrease in instrument sensitivity due to solar degradation of the instrument’s single ESR. The follow-on ACRIM-1 helped explain that this trend was, in fact, solar.

#### ACRIM-1, -2, and -3

**Fig. 9:**
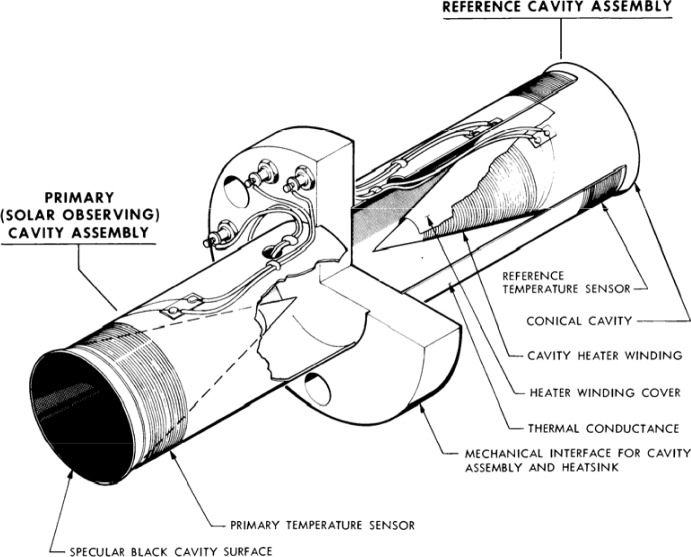
ACRIM ESR. The back-to-back ACRIM ESR-cavity design provided thermal stability and low sensitivity to common-mode thermal changes. Three such back-to-back conical-cavity pairs per instrument provided redundancy and enabled degradation tracking. (courtesy R. Willson 2012, personal communication)

The NASA/JPL’s Active Cavity Radiometer Irradiance Monitors (ACRIMs), a series of three spaceflight instruments, began with the launch of the ACRIM-1 on the SMM and was followed by the ACRIM-2 on the Upper Atmosphere Research Satellite (UARS) and the ACRIM-3 on the free-flyer ACRIMSat. These were essentially 3-channel versions of the ACR IV used in the sounding rocket described in Sect. 2.1 such that each ACRIM instrument utilized three nearly identical pairs of conical-cavity ESRs mounted back-to-back around a central heat sink, providing common-mode stability to each pair (see Fig. 9; Willson 1979). A precision 5-mm aperture is immediately in front of each ESR. Unlike the ERB, in the ACRIMs the open mouth of the cone faces the Sun and collects incoming light. A specular black paint used as the absorptive surface inside the cavity allows six specular reflections in the 30°-apex cone before reflecting light back out the mouth, giving high absorptivity. The three redundant pairs could be duty cycled to determine (and thus correct in ground processing) solar-exposure-caused degradation in the primary, improving measurement stability over that of the predecessor ERB. Typical degradation amounts for the three spaceflight ACRIM instruments were < 0.1% over their lifetimes. The degradation in all three ACRIMs is mainly due to brightening of or surface changes in the black paint, as hydrocarbon-based paints are susceptible to bond-breaking from ultraviolet light. All subsequent TSI instruments except the ERBS/ERBE used a similar multiple-cavity degradation-tracking technique. The ACRIMs’ shutter period of 128 s, much longer than the servo-system settling time, enabled individual TSI measurements at that period. The 1.024-s individual-sample duration gives even higher cadences when the shutter is open, albeit with more noise. Nearly 10,000 such samples were acquired daily during the two periods of good solar pointing on the ACRIM-1 (Willson and Hudson 1991; see mention below of the SMM’s inadvertent spin mode).

**Fig. 10:**
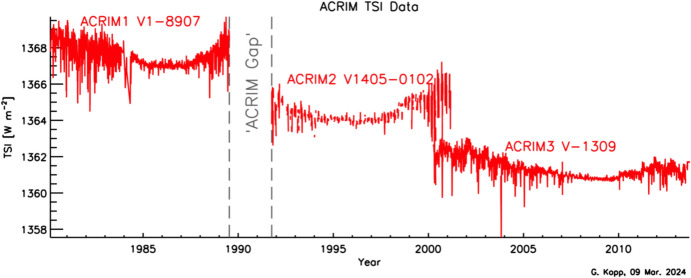
ACRIM TSI data. The daily ACRIM-1, -2, and -3 TSI values are plotted. Other than the “ACRIM Gap,” the measurement gap between the ACRIM-1 and ACRIM-2, these instruments provided the primary NASA TSI measurements for 3.5 decades. Scale differences between them are indicative of the uncertainties in these three instruments. Only the ACRIM-3 data have been corrected for internal-instrument scatter, decreasing those values to nearly match the lower TSI value established by the SORCE/TIM; the older ACRIMs’ data were never corrected by the ACRIM team

The ACRIMs were built by TRW for JPL. The ACRIM-1 and ACRIM-3 used a pointed conical tip, while the ACRIM-2 attempted to eliminate the reflection caused by a paint meniscus in that tip with a narrow, hollow, bent tubular cylinder at the cone’s apex. The latter, however, led to higher uncertainties due to non-equivalence effects. Although they were intended to be accurate to ~ 0.1% (and the ACRIM-2 to ~ 0.2%), ground calibrations gave differences of 0.5% in the values of the measured TSI even between the three ACRIMs, casting these intended accuracies in doubt. Without sufficiently low absolute uncertainties, the ACRIM-era TSI record relied on measurement continuity via overlap from stable on-orbit instruments. The three ACRIMs were intended to provide this continuity for NASA. While the ACRIM-2 and ACRIM-3 overlapped temporally, the ACRIM-1 and ACRIM-2 did not due lack of launch opportunities after the Space Shuttle Challenger launch disaster. This caused a 3-year gap in the ACRIM measurement record (see Fig. 10). Although this gap was spanned by both the Nimbus-7/ERB and the ERBS/ERBE (see Fig. 6), which of those two instruments is chosen to fill that gap leads to differing trends in the solar irradiance (Fröhlich and Lean 1998; Fröhlich 2006; Willson and Mordvinov 2003; see Sect. 3).

The ACRIM-1 measurements were affected by the SMM’s “spin mode,” when it lost accurate pointing control in Nov. 1980 and was put into a slow spin to maintain stability for the solar arrays. The ACRIM-1 spin-mode measurements were noisier and affected degradation, as the instrument had nearly 100 × lower solar-exposure rate during that time. Only ~ 100 samples were acquired per day during this spin mode. The SMM’s pointing was corrected by the STS-41C Space Shuttle servicing mission in the spring of 1984, after which normal operations resumed.

As with the ERB (Sect. 2.4.1), the concurrent ACRIM-1 recorded a downward trend in its initial measurements that was similarly attributed to a decrease in instrument sensitivity with time. Later, when the next solar cycle began and the TSI increased after 1987 (and having trust in the degradation-correction methodology employed by the ACRIM-1), Willson and Hudson (1988, 1991) showed that the TSI, while decreasing on short timescales due to sunspots, varied in-phase with the solar cycle on long timescales. This was a seminal finding enabled by the stability of the ACRIM-1, which the team (perhaps optimistically) estimated to be as low as 0.005% decade^−1^ (Willson and Hudson 1991). The ACRIM-1 also validated the downward trend of the earlier ERB, with Hickey et al. (1988) suggesting that it was only the combination of both instruments showing the same trend that made it trustworthy.

The ACRIM-2 was assessed to have similar levels of stability to the ACRIM-1 after degradation corrections, while the ACRIM-3 was claimed to have an even more optimistic 0.001% decade.^−1^ stability uncertainty (Willson 2001). The ACRIM-3 data are available from NASA at https://catalog.data.gov/dataset/acrim-iii-level-2-daily-mean-data-v001

#### ERBS/ERBE

The NASA’s Earth Radiation Budget Experiment (ERBE) on the Earth Radiation Budget Satellite (ERBS) provided one of the longest-duration measurements of TSI with the mission’s 19-year lifetime. Although it lacked redundant channels for degradation tracking, it was claimed to have good inherent on-orbit stability due to extremely low solar exposure throughout the mission. This instrument was a single-channel ACRIM-design ESR measuring outgoing broadband radiation from the Earth. For reference calibrations, the ERBE also acquired solar-irradiance measurements by allowing the ERBE to scan across the Sun as the spacecraft orbited the Earth, similarly to the observing approach of the NIMBUS-7/ERB. For the ERBE, this gave a 3-min TSI measurement once every 2 weeks. While not ideal for detecting short-term solar-irradiance variability, this observing duration and cadence gave very little solar exposure over the duration of the mission, presumably leading to good long-term inherent measurement stability, and no degradation corrections were applied to this instrument by the team. Battery problems on the ERBS required the ERBE to be powered down for 4 months in 1993, causing a data gap from 17 July to 22 Nov. of that year.

**Fig. 11:**
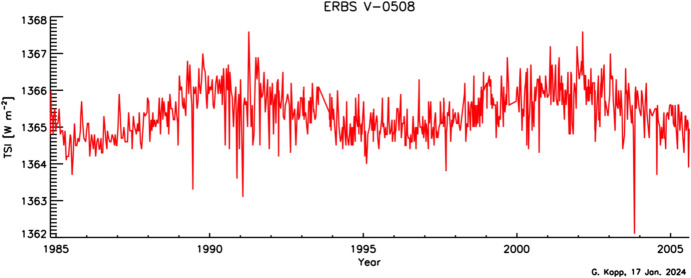
ERBE TSI data. This single-channel instrument spanned nearly two solar cycles with very little presumed degradation. It acquired only sparse (bi-weekly) measurements and suffered from high instrument noise

While indications are that this instrument acquired little solar-exposure-caused degradation and therefore was very stable, the measurements from the ERBE show high noise, as indicated by the large amount of measurement scatter that does not decrease during solar minimum (see Fig. 11), when the Sun is less variable. This is likely due to pointing sensitivities in the passively scanned solar measurements. This instrument’s data are available from the NASA-hosted site https://catalog.data.gov/dataset/earth-radiation-budget-experiment-erbe-total-solar-irradiance-tsi-from-the-earth-radiation/resource/4a3cdb46-b7d2-46c8-8433-3ba0524b8e77

#### SoHO/VIRGO

**Fig. 12:**
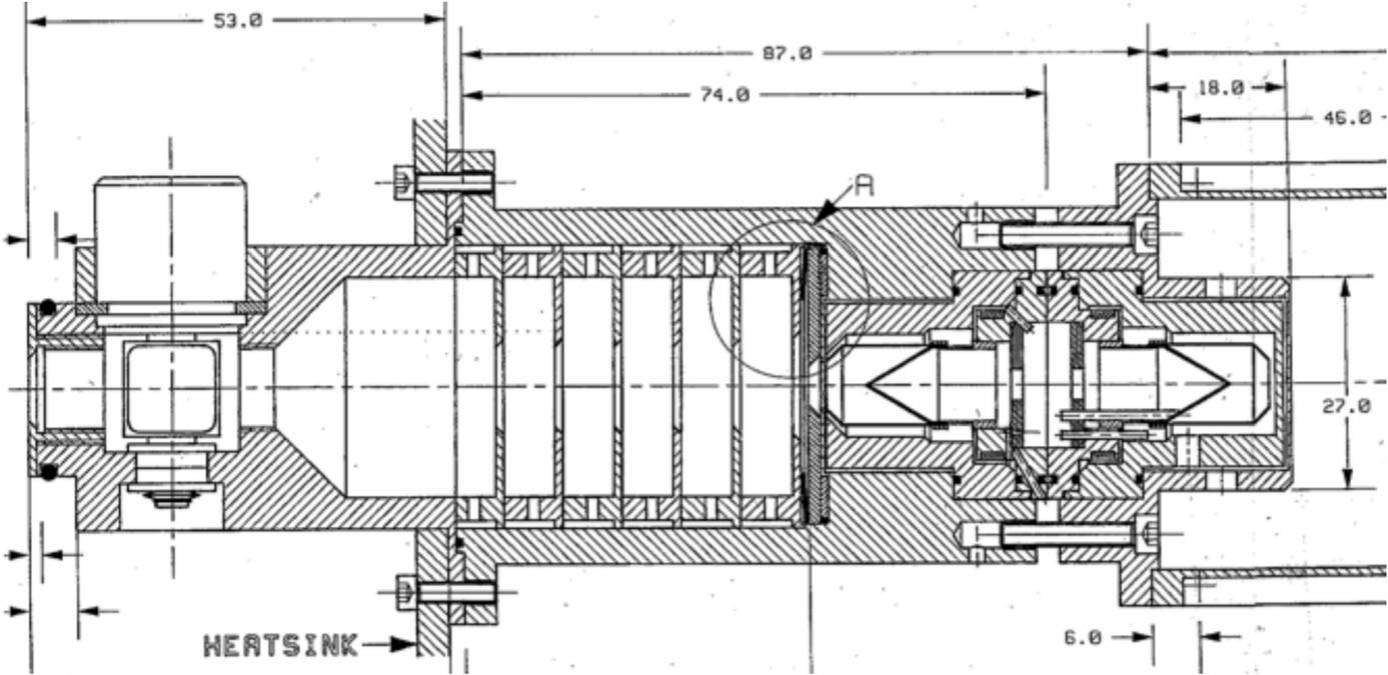
Cutaway of one VIRGO PMO6 cavity pair. The PMO6 portion of the VIRGO used two pairs of back-to-back cavities with an inverted geometry, such that the cone apex faced toward the entering sunlight, which, in this figure, enters from the left. Note the primary aperture, designated by the circle marked “A,” is deep inside the instrument, immediately in front of the primary ESR cavity. Several upstream baffles and other portions of the instrument are illuminated by sunlight and can scatter additional light into the primary ESR, as described in Sect. 2.4.5. (courtesy of C. Fröhlich, personal communication)

The ESA’s Solar and Heliospheric Observatory (SoHO) Variability IRradiance Gravity Oscillation (VIRGO; see Fröhlich et al. 1995, 1997) contains three irradiance monitors. Two absolute radiometers, the Differential Absolute Radiometer (DIARAD) and the PMOD/WRC PMO6 (shown in Fig. 12), measure TSI, while the SunPhotoMeter (SPM) measures spectral solar irradiance in three bandpasses (see Sect. 2.5.3.1).

The original VIRGO PI, Claus Fröhlich, used the different responses, measurement cadences, and degradation of the two TSI instruments to estimate and apply corrections for each, with the expectation that long-duration changes between the two instruments, which have very different optical designs and measurement principles, can indicate anomalous responses in one or the other. In cases of discrepancies between the PMO6 and the DIARAD, Fröhlich initially used comparisons to other on-orbit instruments, namely the ACRIM-2, ERBE, ACRIM-3, and SORCE/TIM, to help discern to which of the DIARAD or PMO6 corrections were applied. He provided the VIRGO data until May 2018, after which they are determined using a new methodology described by Finsterle et al. (2021) from the host PMOD institute. Both the older and newer data are available on daily, hourly, and 1-min cadences via ftp://ftp.pmodwrc.ch/pub/data/irradiance/virgo/TSI/.

**Fig. 13:**
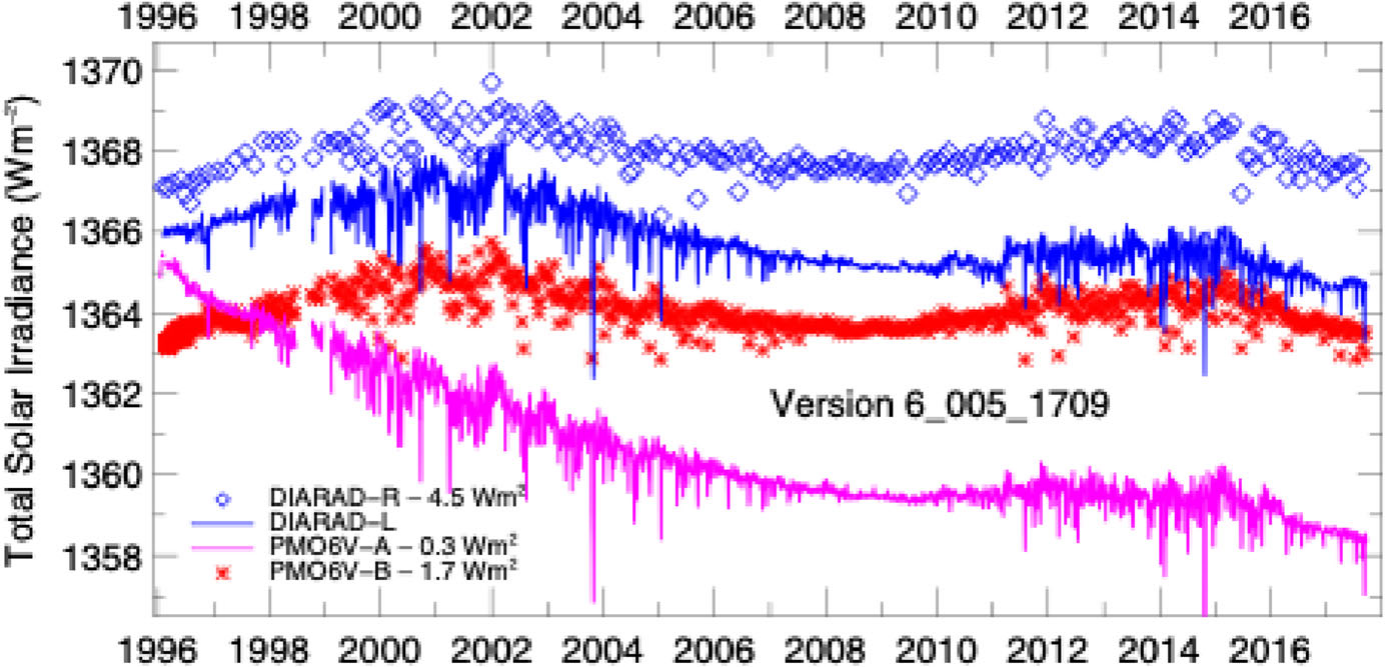
VIRGO degradation measurements. Measurements with each of the four cavities in the VIRGO enable degradation tracking due to solar exposure. The PMO6V-A shows greater degradation than any prior TSI radiometer. Measurements with PMO6V-B, used 150 × less, help correct this. The primary DIARAD channel, DIARAD-L, shows much less long-term degradation. The DIARAD pairs are used to track each other as well as allow additional corrections to the PMO6 channels. (Plot courtesy of C. Fröhlich, VIRGO PI. The plotted data are on the VIRGO’s “original” scale and have not been corrected to the newer lower value.)

The VIRGO has two major advantages over most other TSI-measuring instruments. The first is duration: The longer a data record is, the more beneficial it is for Earth-climate and secular solar-variability studies. The VIRGO TSI measurements currently span nearly 3 decades, having started operations in early 1996, giving it the longest-duration TSI-measurement record of any spaceflight instrument (see Table 3 and Fig. 13). The second is due to the SoHO’s location: It is at the L1 Lagrangian point, giving the instrument uninterrupted views of the Sun. All other TSI instruments are in LEO and have times when the Sun is occulted by the Earth. The nearly continual, high-cadence (1-min) VIRGO TSI record enables spectral-power analyses of solar variability to high frequencies with very little windowing.

There is a gap in the VIRGO data from 23 June to 6 Oct. 1998 due to a commanding error that mis-pointed the SoHO spacecraft, causing its batteries to discharge. (For details of the cause, see the “SoHO Mission Interruption Joint ESA/NASA Investigation Board Report,” 1998.) This “SoHO Vacation” had a fortuitous self-recovery, as the spacecraft awoke a few months later when its orbit around the Sun illuminated the solar panels. Fröhlich and Finsterle (2001) go to substantial efforts, including comparisons to the ACRIM-2 (which spanned this VIRGO data gap with lower noise than the ERBE), to ascertain corrections for gap-induced changes presumed due to thermal effects during the unpowered SoHO Vacation (as well as other instrument power cyclings) in each of the VIRGO radiometer responses. Some corrections across the SoHO Vacation are ~ 0.03%.

The VIRGO’s PMO6 radiometers used two pairs of back-to-back cavity pairs that are duty cycled in a 150:1 ratio to account for solar-exposure-caused degradation. A cutaway of one pair is shown in Fig. 12. The PMO6 cavities used an inverted conical-cavity design such that the cone’s 60° apex faces toward the entering sunlight. The specular black paint on the outside of this inverted cone reflects light onto the interior of the surrounding, black, cylindrical walls of the ESR cavity, as in the NIMBUS-7/ERB. These are mounted immediately behind a 5-mm-diameter precision aperture.

**Fig. 14:**
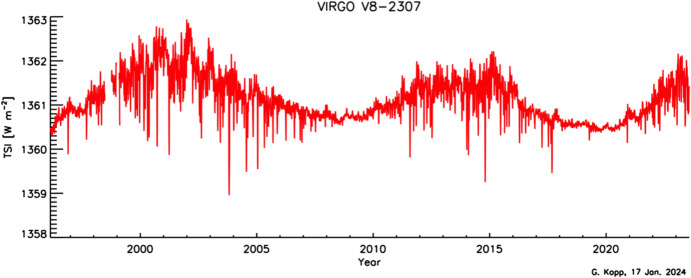
VIRGO TSI data. The VIRGO provides the longest-duration TSI measurement record of any single instrument

The primary VIRGO PMO6 radiometer, PMO6V-A, degraded ~ 0.4% in sensitivity over the mission’s duration (see Fig. 14). This is more than any prior instrument and is primarily thought to be due to brightening of the cavity’s interior black paint. Oddly, this instrument showed an initial ~ 0.06% *increase* in sensitivity before the longer and more pronounced decrease. Such a reversal had not been definitively observed in any prior TSI instrument, although, aside from the ACRIMs, other instruments had no means of tracking degradation; although, as shown in Fig. 8 and discussed in Sect. 2.4.1, Fröhlich did suspect an early increase in the ERB.

Actively pointed TSI instruments use mechanical shutters to modulate incident sunlight, as described in Sect. 2.3.1. Typically operating at periods of 90 to 130 s, this modulation allows corrections for the instrument-internal thermal background on timescales faster than that background varies. The VIRGO’s PMO6 radiometer shutters failed shortly after launch, so, instead of cycling at the intended 120-s period, they remained open and a protective cover at the front of the instrument was occasionally used to block incoming sunlight to obtain thermal-background measurements. This mechanism was not designed for frequent use, so was only cycled on an 8-h cadence, making the instrument more susceptible to higher-frequency thermal changes. Helpfully, being at L1, the VIRGO is in a more stable thermal environment than TSI instruments in low-Earth orbit.

The DIARAD utilizes a different operating principle, with an instrument design consisting of two Sun-facing cylindrical cavities containing a diffuse black paint on their interiors. These are duty cycled in a 1200:1 ratio to account for degradation in the primary (DIARAD-L). The heat flux through the rear of the cavities into the heat sink is measured to determine the incidence power. As with all other TSI instruments up to this point, the primary aperture is deep inside the instrument, which, as discussed in Sect. 2.4.5, causes excess scatter and erroneously high signals, if uncorrected; however, the DIARAD has a novel aperture design with the front (solar-facing) surface being concave such that it focuses much of the incident sunlight reflected from its front surface out through the large, view-limiting instrument-entrance aperture, reducing internal-instrument scatter. Shutters modulate the incident sunlight with a 90-s period, enabling TSI measurements at that cadence.

A large determiner of the VIRGO measurement uncertainty is the spread of the on-orbit TSI measurements from each channel. The two DIARAD channels measure on-orbit TSI values that differ by 3.76 W m^−2^ for reasons that were never ascertained. The PMO6 channels differ by even more, at 4.41 W m^−2^. The standard deviation of the large on-orbit channel-to-channel differences contributes to a VIRGO measurement uncertainty of 0.18% (Fröhlich 2014).

An early consensus within the VIRGO team was that both the PMO6 and DIARAD radiometers would be combined to produce a single VIRGO TSI data product and separate products from each instrument would not be released. While adherence did not persist, in this article I refer only to the single, combined-radiometer VIRGO product released by the original and subsequent PMOD PIs.

#### SORCE/TIM

The NASA’s SOlar Radiation and Climate Experiment (SORCE) Total Irradiance Monitor (TIM) is the first of the modern-design TSI instruments with several advances described by Kopp and Lawrence (2005) that helped it establish a ~ 10 × more accurate measurement of the TSI than prior instruments due to a different optical design that reduced internal-instrument scatter (see Kopp and Lean 2011). This new, lower TSI value of 1361 W m^−2^, a seminal finding of the SORCE/TIM, is now the IAU-accepted mean TSI value (Prša et al 2016) and defines the Earth’s net incoming energy.

**Fig. 15:**
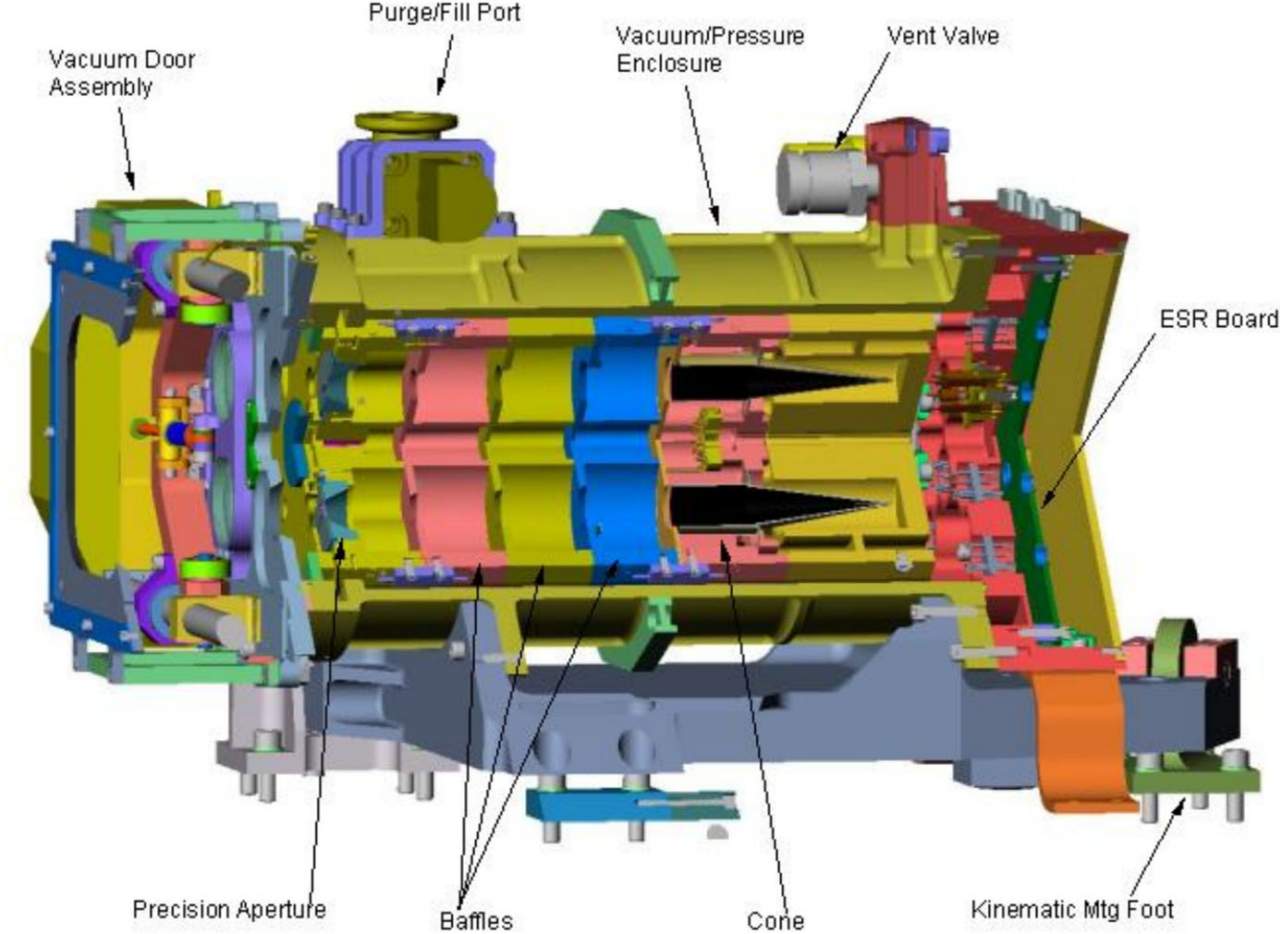
TIM instrument cutaway. Four black absorptive cavities (two shown) measure solar power passing through precision apertures in a temperature-controlled instrument. In this drawing, sunlight enters from the left, passing through a small front-mounted light-limiting precision aperture before reaching the ESR cavities deep within the instrument interior. This geometry, used on most subsequent TSI instruments, only allows the light desired to be measured into the instrument, reducing internal-instrument scatter that plagued prior instruments. (Compare to VIRGO PMO6 design in Fig. 12.)

A cutaway of the SORCE/TIM is shown in Fig. 15. Each of the four TIM ESRs is a 20°-apex conical cavity with the mouth facing toward entering sunlight to encourage scattered light to bounce deeper into the cone interior for subsequent absorption. A cylindrical extension on the mouth of the cone further helps capture light that is not absorbed deeper inside, increasing effective efficiency. All four ESRs face toward the entering sunlight rather than being in the back-to-back configurations of many prior instruments. This enables any of the four to view the Sun, providing four-way redundancy and enabling four-way degradation tracking, which is done in 100:1, 200:1, and 500:1 ratios. All are axially mounted to a common heat sink to reduce common-mode sensitivities to thermal fluctuations from the surroundings.

The primary novel features of the TIM over prior instruments include:

- The precision 8-mm-diameter aperture, defining the area over which sunlight is collected, is at the front of the instrument. It thus only allows light into the instrument that is intended to be measured and thereby reduces internal scatter. Pre-launch calibrations at the NIST/Gaithersburg determined the diamond-turned precision-aperture areas to 0.0025% (*k* = 1) uncertainty.
- Nickel phosphorus (NiP) is used as the black absorptive coating inside the ESR cavities. Being a metal, this provides better thermal conductivity and robustness than the hydrocarbon-based black paints used on all prior instruments. Flight results show the best intrinsic stability of any prior TSI instrument, with up to 16 × lower degradation. NiP is diffuse and not as absorptive as specular blacks in conical-cavity geometries, requiring characterizations of the cavity reflectances across the solar spectral range (Kopp et al. 2005a, b).
- Digital control of the servo systems regulating ESR temperatures for greater stability and on-orbit programmability.
- Phase-sensitive detection (Gundlach et al. 1996) in the instrument’s thermal-control servo systems and in ground processing of the data reduces sensitivity to thermal drifts, 1/*f* noise, and parasitic thermal variations that are not at and in-phase with the shuttered sunlight, giving a noise level of  < 4 × 10^–6^. This method is unique to the TIM instruments, as all others are analyzed purely in the time domain, so include contributions from the entire power-spectral range of background sources.

The most significant improvement implemented by the SORCE/TIM is having the precision aperture as the foremost element. All prior instruments have larger view-limiting apertures as their first sunlight-facing element and the smaller precision aperture deep inside the instrument near the radiometer cavity (see, for example, Figs. 7 or 12). These prior designs allowed two to three times the amount of light as is ultimately intended to be measured into the instrument interior, and any scattering of this excess light into the cavity itself caused an erroneously high measurement. These scatter effects were shown to cause errors of ~ 0.4% based on ground versions of existing instruments (Kopp and Lean 2011).

With the TIM’s aperture placement, the instrument gave lower TSI values of ~ 1361 W m^−2^, whereas prior instruments reported values nearer to 1366 W m^−2^, or 0.35% higher (see Fig. 23). After several years of TSI-community discussion, lab-based diagnostic experiments, and recalibrations of engineering models of other instruments on the ground-based SI-traceable TSI Radiometer Facility (TRF; Kopp et al. 2007), the largest cause of the differences was established as uncorrected internal-instrument scatter in prior instruments causing erroneously high TSI values (Kopp and Lean 2011). As with the initial uncertainty about the TSI varying in-phase with the solar cycle described in Sect. 2.4.1, this transition to a new, lower value of the TSI took several years of community discussion. A chronology of those efforts is given in Sect. 2.4.12.

Aperture placement and other SORCE/TIM advances provided measurement uncertainties of 0.035%, while other instruments reported estimated uncertainties of ~ 0.1%. (These latter were actually shown to be ~ 0.4% based on the absolute-value errors ascertained in the reported measurements.) The TIM’s NiP provided total degradation of a mere 0.025% over the SORCE mission’s 17-year lifetime (compare to Fig. 13).

**Fig. 16:**
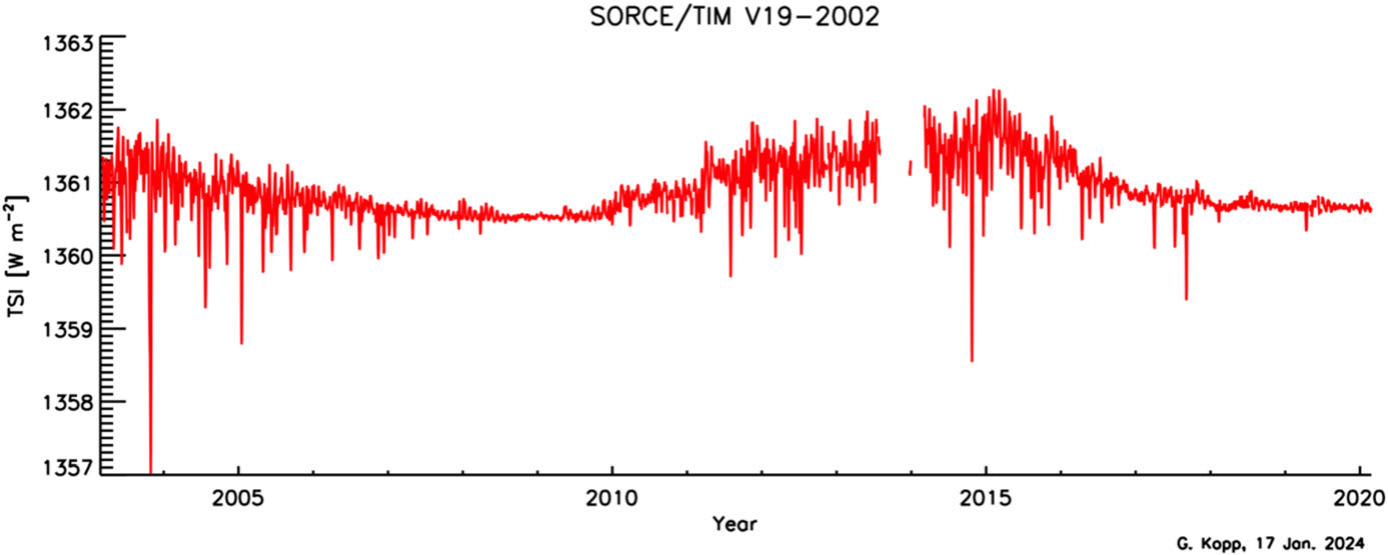
SORCE/TIM TSI data. SORCE/TIM, being a new instrument design that achieved roughly 10 × lower uncertainties than prior instruments, established the now-accepted lower mean TSI value of 1361 W m^−2^

The SORCE/TIM’s 17-year measurement record is shown in Fig. 16. The instrument modulates incident sunlight with a bistable 10-ms open/close shutter operating at 0.01 Hz and produces TSI data on a 50-s cadence with its noise-reducing phase-sensitive-detection methodology. Dark-corrected data meeting acceptable criteria for instrument temperatures, glint, and pointing are averaged into both 6-hourly and daily data products, which are available at https://lasp.colorado.edu/home/sorce/data/tsi-data/.

There is a gap in the SORCE data from 30 July 2013 to 5 March 2014 (with a few days of data in Dec. 2013 to acquire overlapping measurements with the newly launched TCTE/TIM) due to the spacecraft’s aged battery being unable to sustain operations during eclipse periods in low-Earth orbit. Daily operations were resumed after software updates enabled the spacecraft computer to autonomously reboot and then restart the instruments after it acquired solar-panel power upon emergence from eclipse on each orbit (see Woods et al. 2021). In ascertaining TIM-measurement stability after its lengthy power off, comparisons with other instruments’ data were analyzed. Before the battery anomaly, other operating instruments included the ACRIM-3, PREMOS (Sect. 2.4.6.1), and VIRGO, and the SORCE-follow-on TCTE/TIM (Sect. 2.4.7) launch was imminent. However, during the gap, communications with the ACRIM-3 were permanently lost, the TCTE/TIM launched but lacked pre-gap data for comparisons, and the PICARD, carrying the PREMOS, was decommissioned, leaving only the VIRGO and solar models to span the gap. Comparisons with those suggested no discernable discrepancies in the SORCE/TIM accuracy to the  < 0.005% level after resuming operations. Uncertainties of this level were applied by Kopp (2021) to post-gap data.

#### PICARD

##### PREMOS

The CNES’s PREcision MOnitor Sensor (PREMOS) measured TSI and SSI (see Sect. 2.5.3.2) from the PICARD spacecraft, which was launched in June 2010 and operated for a little less than 4 years before being decommissioned (Schmutz et al. 2013). The PREMOS TSI instrument was a rebuild of the 2-channel, inverted-cone, back-to-back VIRGO PMO6 radiometers, even applying interior specular black paint from the same paint container as used for the VIRGO. Its primary channel thus, unsurprisingly, followed a similar on-orbit degradation change as the VIRGO’s PMO6, having an initial 0.06% increase in sensitivity before suffering the same large (~ 0.4%) decrease. Ball et al. (2016) characterized this sensitivity change, taking advantage of inverting the instrument radiometer pairs’ duty cycling ratio prior to the mission’s termination and verifying that the secondary radiometer experienced the same solar-exposure-dependent degradation pattern as the primary. Instrument operations were identical to those of the VIRGO, although the PREMOS was able to operate its shutters at the intended 120-s period and flew in a near-polar LEO that provided near-continual solar viewing aside from seasonal eclipses by the Earth’s limb.

**Fig. 17:**
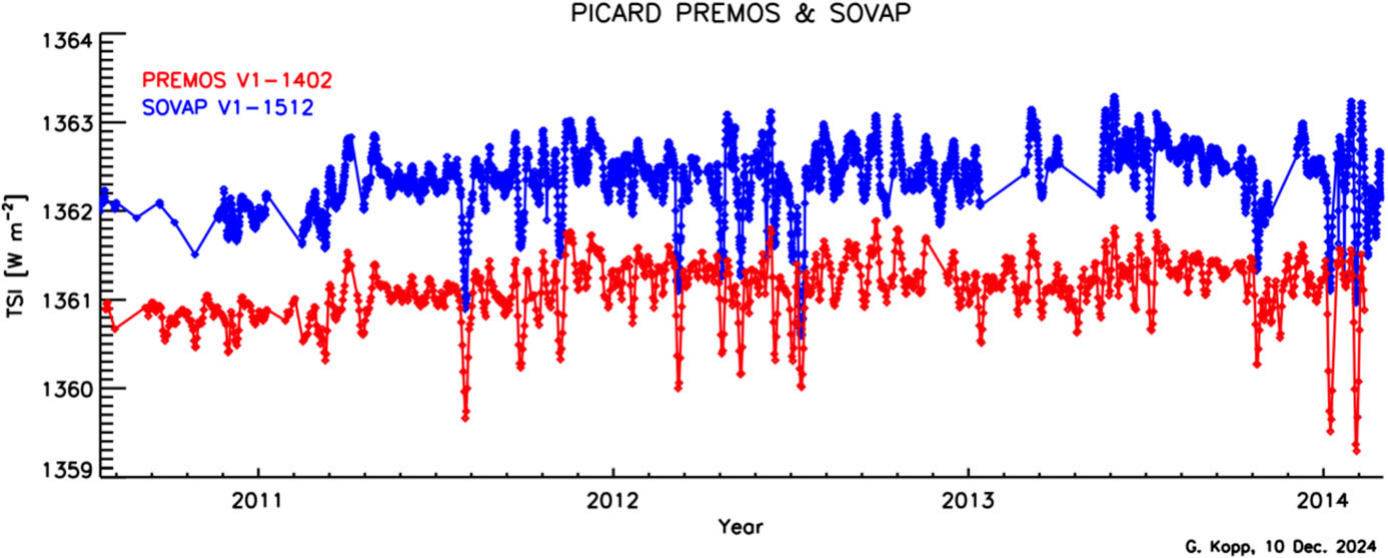
PICARD PREMOS and SOVAP TSI data. The PREMOS was the first TSI instrument to transfer TSI calibrations from the ground-based SI-traceable TRF to space. Its results support the now-accepted lower TSI value established by the SORCE/TIM. The SOVAP reported measurements much lower than those on the VIRGO/DIARAD but still higher than other concurrent TSI instruments. Other than the scale difference, the two agree well on TSI variability

Although the mission duration was short for climate science, the primary science contribution from the PREMOS was transferring its ground calibrations on the relatively new (at the time) TRF to space and confirming the lower TSI value of 1361 W m^−2^ established by the SORCE/TIM. The resulting PREMOS data are shown in Fig. 17 and are available at http://idoc-picard.ias.u-psud.fr/sitools/client-user/Picard/project-index.html.

##### SOVAP

The PICARD mission also included the SOlar VAriability PICARD (SOVAP), which acquired measurements from 22 July 2010 to 2 Mar. 2014. This instrument is a DIARAD like that flown on the SoHO/VIRGO and is operated the same way. The SOVAP was largely overshadowed by the accuracy improvements provided by the TRF-calibrated PREMOS; the SOVAP lacked those end-to-end calibrations, and initial pre-degradation on-orbit SOVAP measurements showed sizeable differences of 2 W m^−2^ (0.15%) between its intended two measurement modes, “Auto2” and “Auto3.” Shortly after operations began, the instrument suffered a shutter failure that left one of the two channel’s shutter open. This limited the degradation-tracking ability and required a new “Rad10” operating mode as well as changes to thermal corrections. Most of the instrument’s data were acquired in this new mode. A side benefit in having a constantly open shutter was acquiring continuous solar measurements, and the instrument was able to produce measurements at 10-s rather than only 90-s cadences. After initially reporting surprisingly high TSI values, a reanalysis by Meftah et al. (2016) introduced a new thermo-electrical non-equivalence term that lowered the SOVAP values to 1361.8 ± 2.4 W m^−2^ representative of the 2008 solar minimum. (This was accomplished using scalings via the VIRGO/DIARAD, as the PICARD did not launch until after that minimum.) While still slightly higher than the TSI values of the concurrent SORCE/TIM, PREMOS, and ACRIM-3, the SOVAP uncertainties place it in agreement with the others.

SOVAP data are available at http://idoc-picard.ias.u-psud.fr/sitools/client-user/Picard/project-index.html and are shown in Fig. 17.

#### STP-Sat3/TCTE/TIM

The Glory/TIM was intended to maintain TSI-measurement continuity after the SORCE mission, but a Taurus launch-vehicle failure destroyed the Glory mission in March 2011. Already 3 years past the end of the SORCE’s prime mission, NASA fast-tracked a replacement TSI instrument. Using one of two already-built laboratory-based TIMs from the SORCE mission, the Laboratory for Atmospheric and Space Physics (LASP), where the SORCE instruments were built and calibrated, refurbished, calibrated, and integrated the replacement instrument for flight in an astonishingly brief 5 months, resulting in the NOAA TSI Calibration Transfer Experiment (TCTE) launched on the Air Force’s Space Technology Demonstration Satellite (STP-Sat3).

**Fig. 18:**
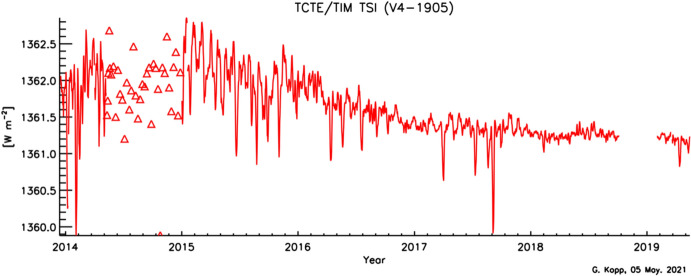
TCTE/TIM TSI data. The TCTE/TIM was a mission to help guarantee overlap between the predecessor SORCE and the successor TSIS-1 TIMs

This TCTE/TIM operated in LEO for nearly five-and-a-half years before being decommissioned. Intended as a “hot” on-orbit calibration transfer instrument in the event of failure of the SORCE mission’s TIM, the TCTE provided only weekly measurements during much of its first year of operation, after initially establishing the needed cross-calibration overlap with the SORCE/TIM. The TCTE operates identically to the SORCE/TIM (Sect. 2.4.5), using the same degradation-tracking ratios for its four channels. The instrument provided direct temporal overlap with both the SORCE/TIM and the successor TSIS-1/TIM, helping mitigate a potential measurement gap in the TSI climate-data record and providing further links between the series of spaceflight TIMs. Checked pre-launch on the TRF (but primarily relying on its superior component-level calibrations), the TCTE/TIM, with data shown in Fig. 18, provided independent validation of the lower TSI value established by the SORCE/TIM to within ~ 0.03%.

Daily and 6-hourly TCTE data are available at https://lasp.colorado.edu/home/tcte/data/.

#### FY-3A to -3F

Part of the China Meteorological Administration’s National Satellite Meteorological Center, the FengYun (FY) is a series of meteorological satellites operating in low- and geostationary-Earth orbits. The LEO satellites FY-3A, -3B, -3C, -3E, and -3F each contain a TSI-measuring instrument called the Solar Irradiance Monitor (SIM) or, beginning with the FY-3E, the Solar Irradiance Absolute Radiometer (SIAR). (Note that the FY-series’ SIM is a different instrument than the identically acronymed SORCE and TSIS-1 Spectral Irradiance Monitor.) Starting with the FY-3C, these SIMs were placed on solar-pointing platforms, giving more stable TSI measurements consistent with cotemporary instruments. The SIM data are publicly available at https://satellite.nsmc.org.cn/DataPortal/en/home/index.html (although they are a bit tedious to access, with each file only covering a very short time duration).

The fifth in the FY series (FY-3E) is providing the best-quality TSI data and, like the VIRGO, contains two different, independent instruments integrated into the Joint Total Solar Irradiance Monitor (JTSIM; Song et al. 2021).

**Fig. 19:**
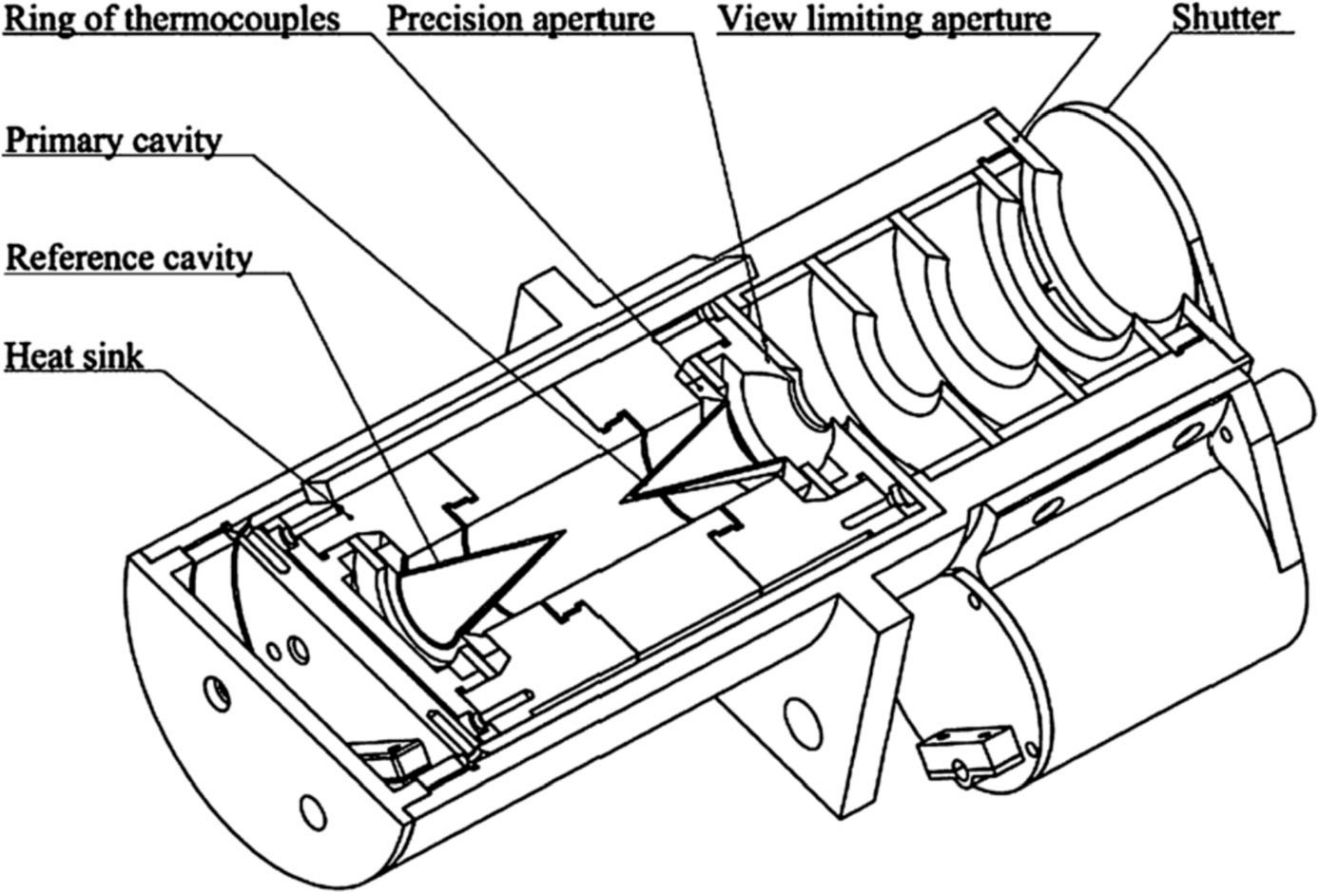
FY-3E SIAR ESR cutaway. The SIAR uses three back-to-back ESR pairs, such as the one shown here. The precision aperture is mounted immediately sunward of the ESR and deep within the instrument, as in older instrument designs. Image reproduced with permission from Song et al. (2021), copyright by the author(s)

The FY-3E/SIAR is a newer version of the prior FY-series’s SIM’s (see Song et al. 2021). It is a three-channel ESR in a linear arrangement with each channel consisting of back-to-back conical cavities with the mouth on the active cavity facing the Sun (see Fig. 19). The cavity interiors are painted with a specular black. The precision 8-mm-diameter apertures are just in front of the Sun-facing cavities, as in the earlier instrument designs, so may be susceptible to scattered light, as those older instruments were. The instrument shutters sunlight with a 120-s period, giving TSI measurements at that cadence.

**Fig. 20:**
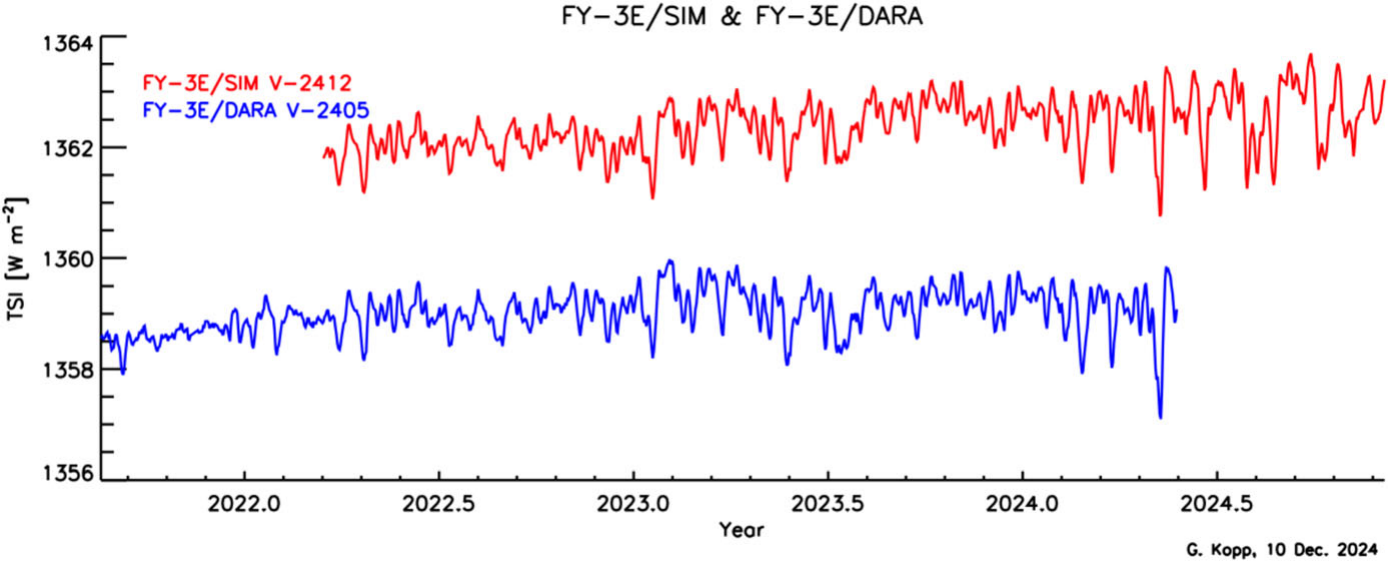
FY-3E TSI data FY-3E/SIM is the best of a series of Chinese Space Agency instruments acquiring TSI measurements that is providing publicly available data. The FY-3E/DARA data have also recently been released. These data are inexplicably lower than the mission’s SIM instrument (or any other currently flying instrument) by more than their combined estimated uncertainties. [The FY-3E mission is owned and operated by the China Meteorological Administration (https://doi.org/10.1007/s11207-021-01794-5). The FY 3E DARA data are retrieved and processed via the cooperation between the Changchun Institute of Optics, Fine Mechanics and Physics Chinese Academy of Sciences (CIOMP/CAS, China), the China Meteorological Administration (CMA, China), and PMOD/WRC (Davos, Switzerland) and are available at the Interdisciplinary Earth Data Alliance (IEDA) https://doi.org/10.60520/IEDA/113059]

The agreement in absolute value of the FY-3E SIAR with concurrent instruments is very good (see Fig. 6). Quoted SIAR uncertainties are 0.0233% based on SI-traceable component-level calibrations. An end-to-end WRR-based calibration gives < 0.099% uncertainties, being larger because of the additional complications of operating in air and accumulating uncertainties from the intercomparison itself. The data from the FY-3E SIM (aka SIAR) are shown in Fig. 20. Unlike all other instruments, the FY-3E/SIM data have no gaps in their reported daily measurements over the nearly 3-year time range currently available.

The FY-3E/JTSIM’s second instrument is a new design from the PMOD that incorporates some of the TIM improvements, such as the front-mounted precision limiting 5-mm aperture and three sunlight-facing (rather than back-to-back) conical-interior absorptive cavities (rather than the inverted cavities in the ERB, VIRGO, and PREMOS) in an ACRIM-like triangular arrangement. Data from this compact Digital Absolute Radiometer (DARA) are also shown in Fig. 20 (Montillet et al. 2024; data are available at ftp://ftp.pmodwrc.ch/pub/data/irradiance/JTSIM/6HourlyRelease/). In addition to the very low values reported from the DARA (which are being reviewed by the instrument team), there are trend differences between the FY-3E/DARA data and those of the FY-3E/SIM even though acquired from the same platform at similar times. These differences are likely attributable to uncorrected thermal effects in the DARA data (personal communication with DARA team).

#### TSIS-1/TIM

The TSIS is a series of intended total- and spectral-solar-irradiance monitors for NOAA and NASA. The TIM on each TSIS suite is a modified version of that first flown on the SORCE, with new ESR electronics for improved power linearity and with redesigned radiometer cavities for better uniformity of the NiP at the conical tip. Built and calibrated at LASP, the first of these, the TSIS-1/TIM, was installed on the International Space Station (ISS) in Dec. 2017. A two-axis pointing system allows solar viewing independent of the ISS platform, although nearby ISS structures and operations frequently preclude observations, reducing the percentage of acquired daily values relative to dedicated free-flyer platforms (see Table 3).

**Fig. 21:**
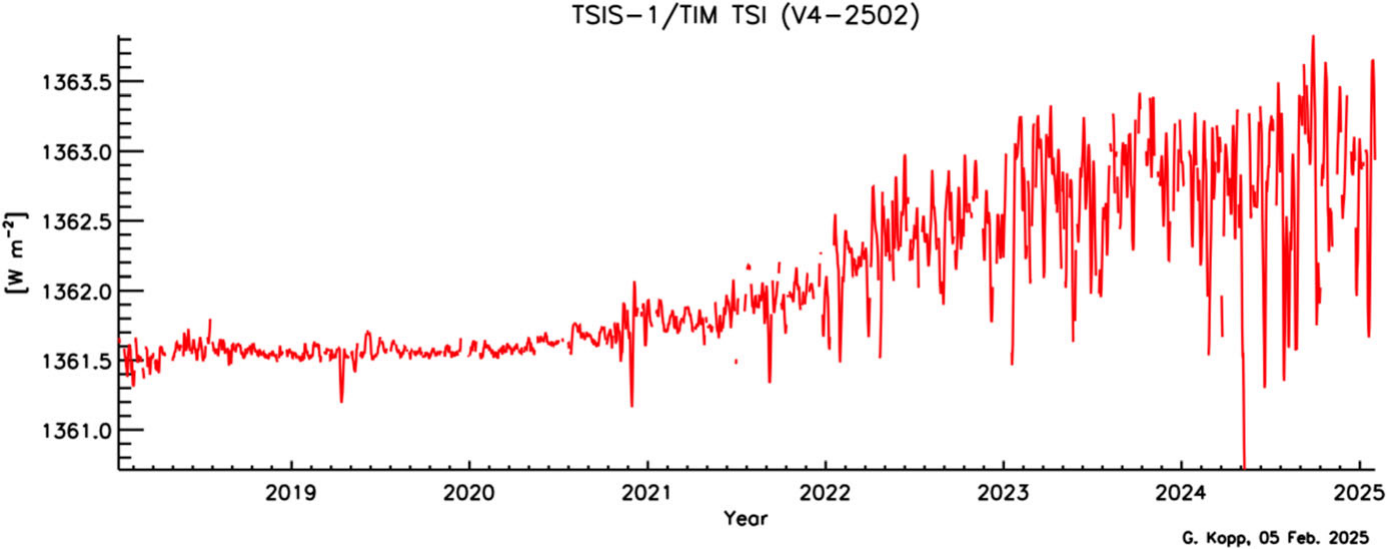
TSIS-1/TIM TSI data. The TSIS-1/TIM, an updated SORCE/TIM instrument, continues the NASA and NOAA climate-data record of TSI

The TSIS-1/TIM has the lowest uncertainties of any spaceflight TSI instrument due to its new electronics and improved component-level ground calibrations. As with the SORCE and TCTE TIMs, the NiP interiors of the TSIS-1/TIM ESRs show the best intrinsic on-orbit stability of any prior flight instrument. Degradation in the primary radiometer is a mere 0.00025% year^−1^ for a total applied degradation correction to date of only 0.0013%. TSIS-1/TIM operates identically to the SORCE and TCTE TIMs and uses shared data processing streams. Data are available at https://lasp.colorado.edu/home/tsis/data/tsi-data/ and shown in Fig. 21.

Builds of TSI and SSI instruments identical to those on the TSIS-1 are underway for the follow-on TSIS-2, which is intended to provide direct measurement overlap with the TSIS-1 from a free-flyer and continue the NASA and NOAA efforts to maintain continuity of these climate-data records. The TSIS-2 is intended for launch in late 2025.

#### CTIM

**Fig. 22:**
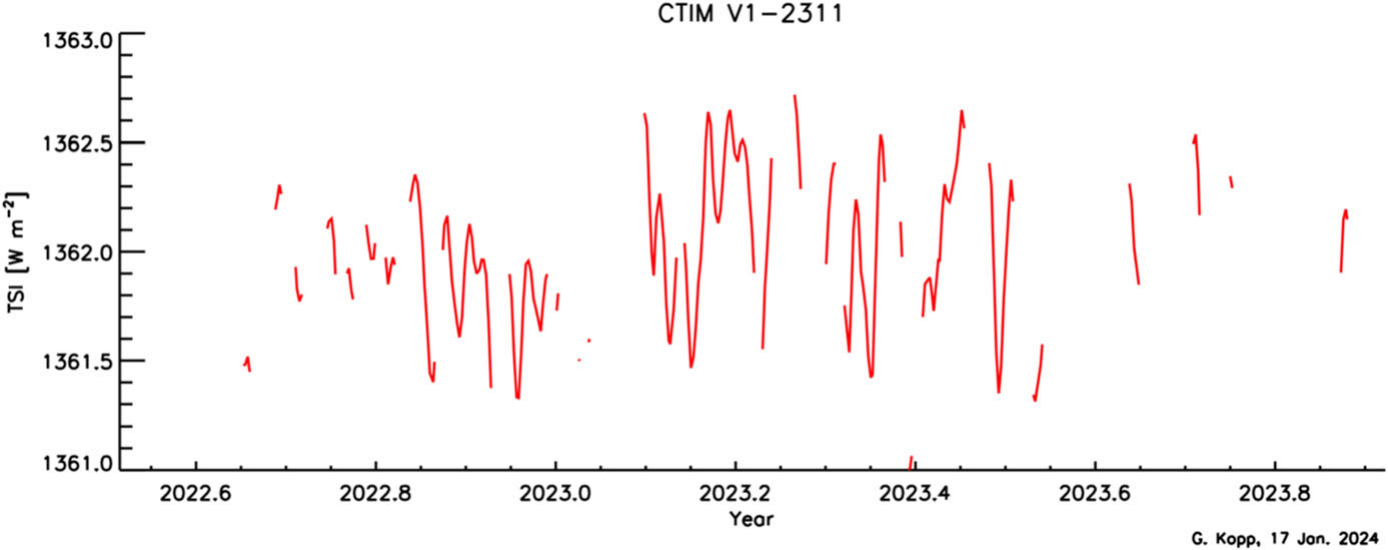
CTIM TSI data. The CTIM is a technology-demonstration mission using miniature ESRs. As a CubeSat-based demonstration, its measurement record has more gaps than most prior instruments, but it agrees well with concurrent instruments in both temporal variability and absolute scale

The Compact Total Irradiance Monitor (CTIM) was a NASA In-Space Validation of Earth Science Technologies (InVEST) program built by LASP (see Harber et al. 2019). This 6U CubeSat contains eight ESRs of a new design utilizing vertically aligned carbon nanotubes (CNTs) coated on silicon, providing low-cost, compact, rapid, and integrated ESR fabrication utilizing photolithography techniques. The CNT coating is more absorptive than the NiP used on prior TIMs and has superior thermal conductivity. Launched in 2022, the CTIM acquired TSI measurements from LEO until the CubeSat reentered the Earth’s atmosphere in late 2023. These data are shown in Fig. 22 and available via https://lasp.colorado.edu/ctim/.

Although this technology-demonstration mission’s short and intermittent data subsample the solar variability too much for long-term Earth-climate or solar studies, they agree well with other current measurements both temporally and on an absolute scale. This mission shows promise for similar future high-accuracy, low-cost, rapid-build TSI instruments. With these virtues, a series of overlapping CTIMs may reduce the likelihood of a TSI-measurement gap compared to fewer and more expensive flagship-mission instruments in the uninterrupted 47-year climate-data record.

#### Other TSI instruments

##### NOAA9

NOAA9 included TSI measurements from 22 Jan. 1985 to 19 Dec. 1989. During this nearly 6-year duration, it acquired a sparse 116 measurements, which averaged a somewhat high 1364.78 W m^−2^.

##### NOAA10

NOAA10 measured the TSI from 21 Oct. 1986 to 31 Mar. 1987, reporting only 14 measurements over its less than 1.5 years of data with an average value of 1363.24 W m^−2^.

##### SOLCON

The SOLar CONstant (SOLCON), a DIARAD-like instrument, flew on the space shuttle in April 1992 and autumn of 1998 (Dewitte et al. 2001). Its primary achievement with these short-duration flights was to determine measurement ratios to longer-duration on-orbit instruments, particularly the ACRIM-2 the ERBE, and the individual VIRGO radiometers, to help determine their long-term stability.

##### EURECA/SOVA2

The SOVA2 flew on EURECA and provided data from 11 Aug. 1992 to 16 May 1993. At only 279 days duration, this record is short for either long-term solar or Earth-climate studies. The TSI values are erroneously high (but not inconsistent with other instruments at the time), averaging 1366.87 W m^−2^ over the period.

##### SOLAR/SOVIM

The SOVIM on the SOLAR mission acquired 31 measurements between 1 May 2008 and 4 Oct. 2008 before electronics issues ended the measurements. The average TSI values reported were 1364.94 W m^−2^.

##### NorSat-1/CLARA

The Compact Lightweight Absolute Radiometer (CLARA) was launched on the Norwegian NorSat-1 micro-satellite in July 2017. Although first-light results were reported (Walter et al. 2018), data from this instrument are not yet publicly available.

#### Chronology of progression to the new, lower TSI value of 1361 W m^−2^

This subsection provides a chronology of the data releases and publications from international efforts that led to the acceptance of the new, lower TSI value and is an example of the importance of open-data policies and collaborative research. (It also shows how science can be affected by social peer-pressure desires to agree with others.)

**Fig. 23:**
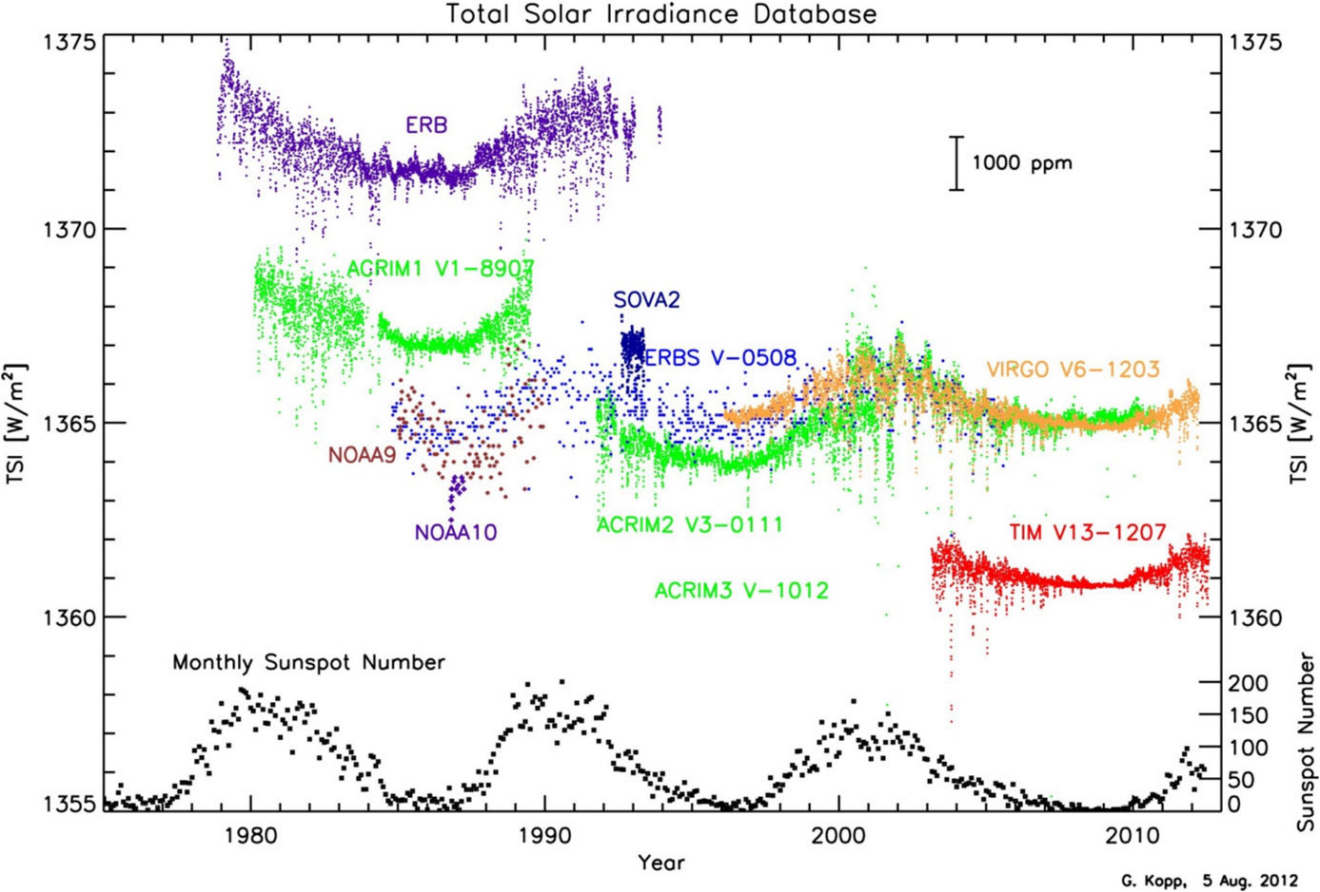
TSI data ca 2012. This is a figure using TSI data from early 2012 to show the state of the measurements at that time. The TSI instruments prior to the SORCE/TIM were in consensus agreement on values near 1366 W m^−2^, making the SORCE/TIM values of 1361 W m^−2^ initially suspect. After nearly 10 years of community discussion and lab tests, the higher values were found to be erroneous. The ACRIM-3 values were retroactively lowered in ground processing in 2011 and then the VIRGO values in 2014. [Includes data from: www.ngdc.noaa.gov/stp/SOLAR/solar.html (NIMBUS7/ERB, ERBS/ERBE, NOAA9, and NOAA10); http://www.acrim.com (ACRIM1, ACRIM2, and ACRIM3); the VIRGO team via ftp://ftp.pmodwrc.ch courtesy of the VIRGO Experiment on the cooperative ESA/NASA Mission SOHO from VIRGO Team through PMOD/WRC, Davos, Switzerland; http://lasp.colorado.edu/home/sorce/data/tsi-data/ (SORCE/TIM).]

- The first two TSI instruments flown, the ERB and ACRIM-1, measured higher TSI values than subsequent instruments, the latter of which (the ERBE, ACRIM-2, VIRGO, and ACRIM-3) were lower and consistent with each other, suggesting that good measurement accuracies had been achieved (see Fig. 23).
- In early 2003, shortly after launch, the SORCE/TIM released data claiming a new, lower TSI value of 1361 W m^−2^ (Kopp et al. 2005b). Figure 23 shows the magnitude of this difference from prior measurements at the time. Because this value was lower than those of prior instruments by more than the claimed uncertainties, NIST and NASA hosted an international workshop in 2005 (Butler et al. 2008) to discuss the calibrations of all current TSI instruments and recommended several optical-power, irradiance, and aperture-area ground validations using primary standards.
- In 2007, The TRF (Kopp et al. 2007) was completed under the NASA Glory project. The Glory/TIM (a newer version of the SORCE/TIM), PICARD/PREMOS, an engineering version of the ACRIM-3, and a radiometer representative of the VIRGO PMO6 were calibrated and/or characterized on the TRF from 2008 through 2010. These results included scatter measurements, which Fehlmann et al. (2012) reported to be “nearly an order of magnitude higher than the original 250-ppm correction previously estimated for PMO6-type [VIRGO and PREMOS] radiometers.” That same article, transferring the PREMOS’s comparisons on the TRF and separate PREMOS-to-WRR comparisons, showed that the WRR, against which several older TSI instruments such as the VIRGO and ERB were calibrated, was 0.34% higher than the true SI scale, suggesting that those earlier-instrument calibrations were erroneously high by that amount.
- In June 2010, the PICARD/PREMOS launched, becoming the first flight instrument to transfer the TRF’s SI-traceable end-to-end TSI irradiance calibrations to space. No results were publicly available for more than a year, leaving the question of absolute value in dispute at the time.
- In March 2011, the Glory/TIM failed to reach orbit due to an Orbital Sciences Corporation Taurus launch-vehicle failure, again leaving a secondary confirmation of the SORCE/TIM’s absolute value unresolved.
- In April 2011, data corrections of ~ 0.5% attributed to previously unknown scatter were applied by Willson (2014) to the ACRIM-3 in ground processing after testing an engineering unit of that instrument on the TRF. This brought that instrument’s released data into agreement with the SORCE/TIM, becoming the first instrument to do so. [The ACRIM team intended similar scattered-light characterizations of engineering units representative of their ACRIM-1 and -2 instruments as well, but never completed those. As the three ACRIM instruments are of nearly identical designs and constructions, expectations are that those earlier instruments would require similar-magnitude TSI-lowering corrections to those of the ACRIM-3, but those corrections were never applied by the team (see Fig. 6).]
- In August 2011, the first PREMOS data were released. They agreed with the SORCE/TIM’s lower TSI value of 1361 W m^−2^.
- The TCTE/TIM (Sect. 2.4.7), validated pre-launch on the TRF, was launched in Nov. 2013 and released data in March 2014. Those data again confirmed the lower TSI value.
- In an unpublished article later that same year, Fröhlich (2014) updated several corrections to the VIRGO instrument, some lowering and some raising the instrument’s TSI values. A new data version in Oct. 2014 lowered the VIRGO values to be 0.0633% below those of the SORCE/TIM. This was followed a month later by another data version that raised the VIRGO to nearly agree with the SORCE/TIM. These newer values are 0.42% lower than the (still quoted as) “original” VIRGO values of ~ 1365.4 W m^−2^. To indicate the extent and complexities of these VIRGO updates, Fröhlich updated the PMO6 corrections to account for ~ 0.30% of scattered light per TRF results described by Fehlmann et al. (2012) and adjusted the VIRGO aperture areas by 0.0650 ± 0.0190% per comparisons with NIST, the non-equivalence by 0.230% for the PMO6–VA channel and 0.334% for the B channel, and aperture heating corrections by 0.0150 ± 0.0080%. These are all intrinsic instrument calibrations. Updates to the WRR calibrations included a 0.0381% change for the PMO6-VA channel and 0.1516% for the B channel as well as the newly described WRR-to-SI scale correction of 0.34% described by Fehlmann et al. (2012). Even with the detail that went into these sizeable changes which lowered the VIRGO values substantially, Fröhlich remained perplexed that the two VIRGO PMO6 cavities differed from each other by 0.3240% and suggested that his estimated absolute uncertainties of 0.1820% (*k* = 1) may be underestimated.

These details illuminate the substantial community efforts that went into understanding the TSI instruments’ accuracies and the large measurement differences from the newer SORCE/TIM, with the result being a lowering of the older instruments’ values and consistency among subsequent instruments. The results of the ground-based intercomparisons (ACRIM-3 and VIRGO) and end-to-end calibrations (PREMOS and TCTE) give the present-day agreement shown in Fig. 6. This more accurate, lower TSI value reduced discrepancies in Earth energy balance estimates (Loeb et al. 2009), so the TSI community’s efforts were highly visible.

### Space-borne SSI measurements

Earth-climate responses to solar forcing are vertically coupled and wavelength dependent (Gray et al. 2010). Understanding these responses relies on sophisticated ocean–atmosphere coupled models and on knowledge of the incident SSI.

In principle, broadband SSI measurements with extremely high levels of accuracy and stability could be spectrally integrated to achieve a TSI value. Currently, however, SSI instruments are limited by their spectral range (200 to 2800 nm at most), accuracies (uncertainties > 0.24%), and stabilities (> 0.01% year^−1^), thus being ~ 15 × higher than the achieved TSI accuracy uncertainties (~ 0.016%) and ~ 10 × higher than the TSI stability uncertainties (0.0012% year^−1^), with the TSI achieving spectral coverage from the soft X-ray to ~ 100 µm. The spectrally broadest SSI measurements give an integrated irradiance of 1324 W m^−2^, which is 97% of the average TSI value (1361 W m^−2^). TSI and SSI measurements are thus complementary: With their superior accuracy, stability, and spectral range, TSI measurements are needed to achieve the long-term climate-driven requirements in Table 2; supplementing those with spectral resolution, SSI measurements help understand the responses of the Earth’s atmosphere and surface via atmospheric-chemistry climate-models.

Spectral-irradiance measurements from 120 to 400 nm have been continual since 1979 from overlapping space-borne instruments. Rottman (2005) summarizes the consensus from these measurements as: (1) the most energetic X-rays can vary by factors of 10 or more, the EUV-spectrum (λ < 120 nm) by factors of 2, and the UV-spectrum (λ < 300 nm was used in the paper’s analysis) by up to 50%; and (2) the spectrally integrated UV (λ < 300 nm) variability accounts for roughly 30% of the solar-cycle TSI variability with the remaining 70% attributed to the visible and infrared. The SSI observational record over solar-cycle timescales in the UV is limited by measurement stability, however, leading to different estimates of variability. Pagaran et al. (2009) estimate 55% of the variability over the solar cycle is from wavelengths below 400 nm by extrapolating their SCIAMACHY measurements on ~ 27-day timescales to the solar cycle. Snow et al. (2022) claim FUV (115 to 200 nm) variations of > 10%

**Fig. 24:**
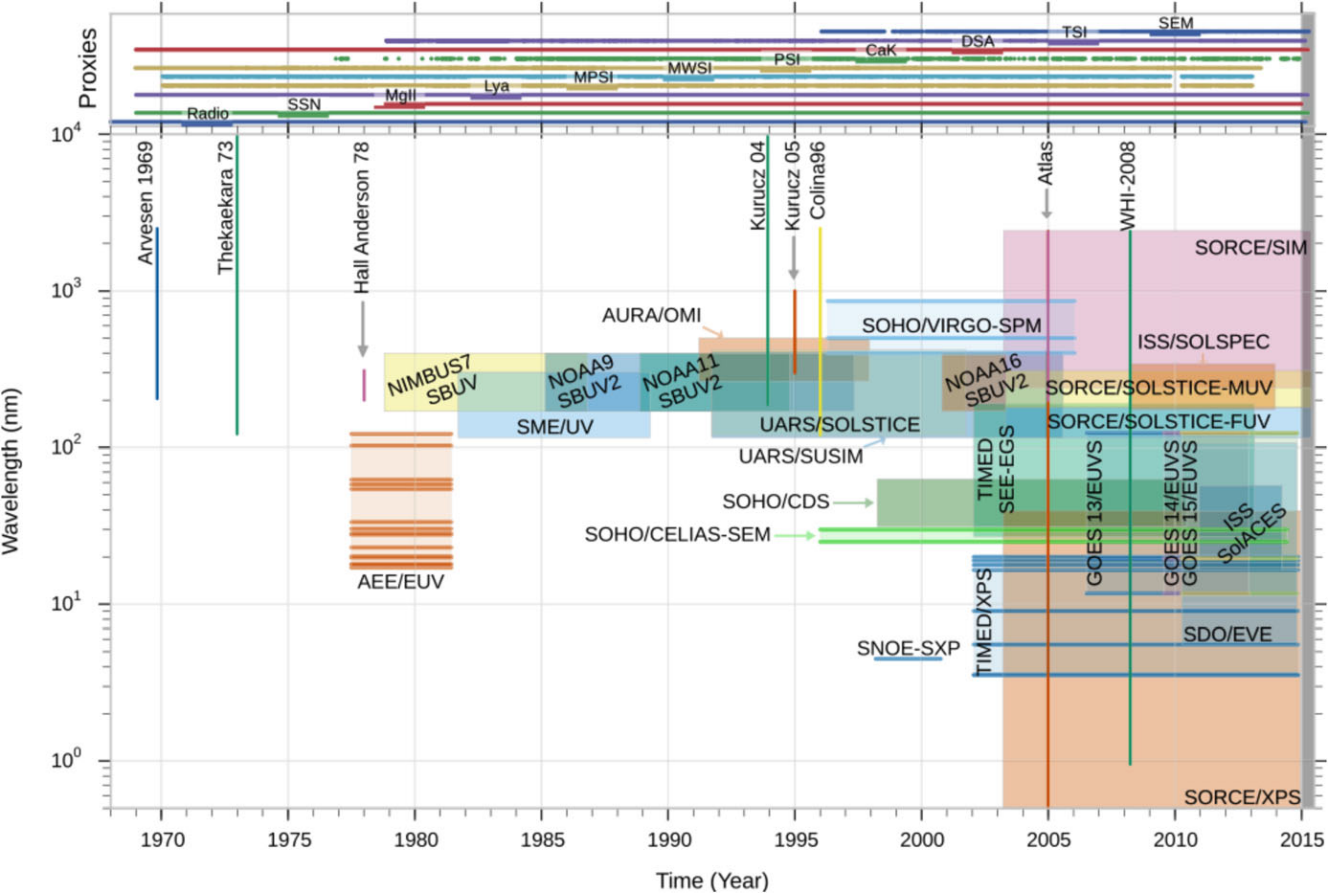
SSI measurements and related data. This plot by Schöll et al. (2016) summarizing the SOLID Composite input data shows select space-borne instruments acquiring SSI measurements and other related data as a function of time and wavelength. Instrument data are shown by the colored boxes, time-specific reference spectra by the vertical lines spanning their wavelength range, and proxy data by the horizontal lines at the top

Starting in 2003, coverage over the UV, visible, and NIR was acquired with radiometric instruments providing daily values. Schöll et al. (2016) created a graphic, reproduced here as Fig. 24, that shows the temporal and spectral coverage of many SSI-measuring instruments.

In this article, we focus on space-based measurements that span a broad wavelength range, excluding many UV- and EUV-only spectra, as they sample only a portion of the net solar irradiance. We consider two aspects of the SSI: (1) What is the solar spectrum? and (2) How does it vary with time?

#### SSI reference spectra

Several reference spectra representative of specific eras in time have been directly measured, and composites extend these to broader spectral regions or mesh them with results having high spectral resolution. Being short-term, these reference spectra don’t individually address solar variability, but they do provide high-quality spectral information. The newer reference spectra have improved accuracies, being derived from the latest SSI measurements. Some are combinations of low-resolution (but accurate) space-based SSI measurements with higher-resolution (but less accurate) spectra that occasionally include modeled spectra. The reference spectra discussed here in chronological order are representative of full-disk irradiances; that is, they give accurate spectral fluxes from the spatially integrated Sun as opposed to specific types of activity on the solar surface. Plots are shown for most reference spectra. Although subtle differences in values cannot be discerned in these figures, they provide a quick visual indication of the spectral ranges and resolutions of each spectrum.

##### JPL ATMOS ATLAS spectra

The NASA/JPL Atmospheric Trace Molecule Spectroscopy (ATMOS) experiment (Farmer and Norton 1989; Farmer 1994), a scanning Michaelson interferometer with a 1-s scan time, acquired some of the first space-based solar spectra. It flew on four space shuttle flights, including the Spacelab 3 (April 1985) and three ATmospheric Laboratory for Applications and Sciences shuttles (ATLAS 1, March 1992; ATLAS 2, April 1993; and ATLAS 3, Nov. 1994). These gave high-resolution spectra free from telluric lines over the infrared spectral range 2 to 16 µm with a resolution of ~ 10^5^. The resulting data show ~ 16,000 solar features, many being diatomic molecular photospheric constituents (CO, CH, OH, and NH) and some ~ 1700 being atomic lines. The instrument’s field of view with its largest field stop is 0.22°, so it cannot acquire full-disk solar integrations needed for irradiance measurements, nor was it calibrated for absolute flux, as its primary purpose was using the Sun as a source for measuring relative Earth-atmospheric absorptions via occultations. These data could, however, be combined with shorter-wavelength spectra to fill in the long-wavelength region with high spectral resolution.

##### ATLAS-1, -2, -3/SOLSPEC and EURECA/SOSP

The National Centre for Scientific Research (CNRS) SOLar SPECtrum (SOLSPEC) experiment on the ATLAS-1, -2, and -3 space shuttle payloads acquired SSI measurements in the spectral range from 180 to 3000 nm with an accuracy of 0.1%. A flight-backup twin, the SOlar SPectrum (SOSP), flew on the EUropean Retrieval CArrier (EURECA) mission. These instruments consist of three double-grating dual-monochromator spectrometers to reduce scattered light. The three cover the UV (180 to 370 nm), visible (350 to 900 nm), and near-infrared (800 to 3000 nm). Spectral scanning acquires a complete spectrum in eleven minutes. Two deuterium lamps for the UV, two tungsten ribbon lamps for the visible and NIR, and one hollow cathode lamp for spectral knowledge provide on-board calibration. A 3300-K blackbody and several tungsten ribbon lamps at the Physikalisch-Technische Bundesanstalt (PTB) provided pre-launch calibrations to an accuracy of 2 to 3% in the NIR.

Thuillier et al. (2003, 2004) used averages of these measurements with modeled high-resolution spectral features from Kurucz (1995) to create the ATLAS-3 reference solar spectrum covering the range 0.5 to 2397.51 nm. EURECA provided most of the IR spectra, while the UV and visible came from the ATLAS missions. Wavelengths below 120 nm were provided by suborbital rocket flights (Woods et al. 1998) and wavelengths from 120 to 200 nm by the mean UARS spectrum (Woods et al. 1996). The composite is then scaled by 1.4% to match contemporary TSI measurements. The resulting reference spectrum has a resolution of 1 nm below 870 nm and 20 nm above. Higher resolution (1 nm) in the IR was obtained by scaling the spectrum of Kurucz and Bell (1995) to the SOSP data, thus including effects of Fraunhofer lines.

**Fig. 25:**
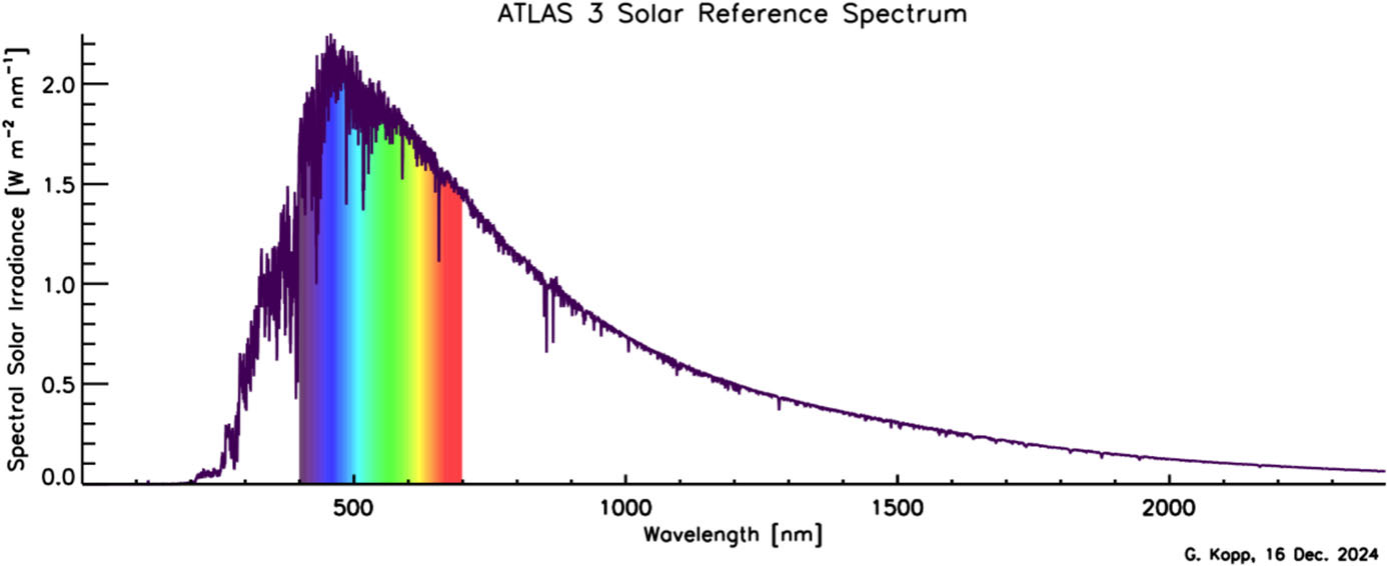
ATLAS reference spectrum. This classic reference spectrum is a composite built around SOLSPEC measurements from ATLAS shuttle flights

The resulting “ATLAS” spectrum, shown in Fig. 25, is often considered the classic reference spectrum against which others are compared; although later measurements, described below, indicate the spectrum measured up to 8% too high in the NIR.

##### SCIAMACHY

Hilbig et al. (2018) produced what they term a reference spectrum from the SCanning Imaging Absorption SpectroMeter for Atmospheric CHartographY (SCIAMACHY; see Sect. 2.5.2.6). The reference-spectrum date, 27 February 2003, was early in on-orbit SCIAMACHY operations, intended to be prior to significant instrument degradation and to best reflect pre-launch calibrations.

More accurately considered here as a “baseline spectrum” to which subsequent instrument degradation corrections were applied, this isn’t a spectrally contiguous reference spectrum as others in this section are due to several inter-channel spectral gaps, making intercomparisons, such as those in Fig. 30 and Table 4, difficult. (See Hilbig et al. 2018 for such comparisons, albeit with spectral gaps.) This spectrum is presented in Sect. 2.5.2.6 and plotted in Fig. 32.

**Table 4:** Portion of energy in different spectral regions

Reference Spectrum	200–400 nm (%)	400–700 nm (%)	700–2400 nm (%)	200–2400 nm [W m^−2^ and % of TSI]
ATLAS-3	8.28	40.26	51.46	1329.9 W m^−2^ (97.71%)
WHI	8.07	40.39	51.54	1309.4 W m^−2^ (96.21%)
SOLSPEC	8.02	40.53	51.45	1321.3 W m^−2^ (97.08%)
HSRS	8.24	40.90	50.86	1310.1 W m^−2^ (96.26%)

##### ISS/SOLAR/SOLSPEC

**Fig. 26:**
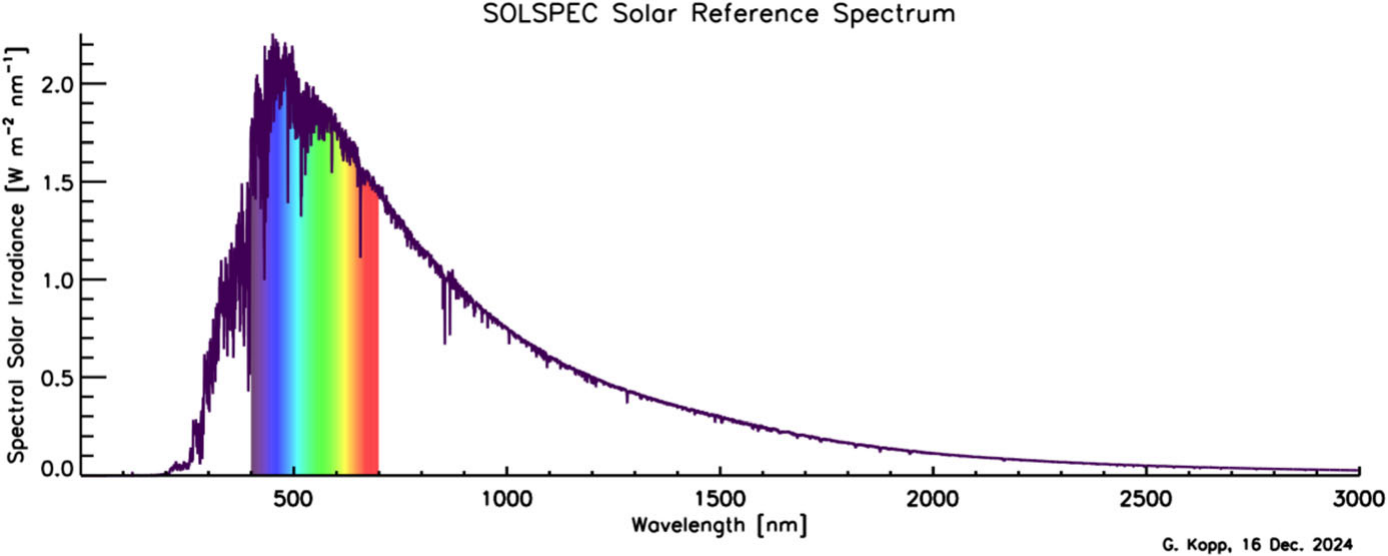
SOLAR-ISS-V2.0 SOLSPEC reference spectrum

SOLAR was launched to the ISS in 2008. The payload consisted of another SOLSPEC wih three pairs of concave holographic gratings in a double-monochromator configuration to cover the UV, visible, and NIR. By combining these measurements with existing, higher-resolution spectra, Meftah et al. (2020) created a solar reference spectrum (SOLAR-ISS-V2.0) representative of the 2008 solar minimum with spectral resolution of  < 0.1 nm below 1000 nm and of 1 nm above. This SOLAR-SOLSPEC-ISS spectrum is shown in Fig. 26. The authors report that the integrated spectrum, corrected for unmeasured out-of-range wavelengths, is 1372.3 ± 16.9 W m^−2^ (*k* = 1), thus being slightly higher than the IAU-accepted TSI value of 1361 W m^−2^.

This spectrum is available at https://dc.g-vo.org/rr/q/lp/custom/CDS.VizieR/J/other/SoPh/295.14.

##### WHI/SIRS

Three Solar Irradiance Reference Spectra (SIRS) were made for three time-periods of the Whole Heliosphere Interval (WHI) spanning Carrington Rotation 2068 (20 March to 16 April 2008) during the extended 2008 solar minimum. These included 25–29 March, 29 March to 4 April, and 10–16 April 2008. The latter is most representative of solar-minimum conditions, while the former two are considered moderately low activity periods, having a small amount of sunspot darkening (with a sunspot number of 33.4) affecting the first period and facular brightening offsetting the sunspot-number count of 16.2 during the second; but these are low-activity effects compared to variations over an entire solar cycle.

**Fig. 27:**
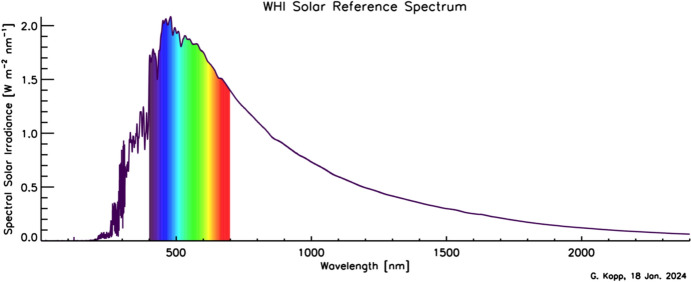
WHI reference spectrum. The lower resolution at visible and NIR wavelengths in this spectrum compared to other reference spectra is because the WHI reference spectrum is purely instrument based and does not attempt to improve spectral resolution with other (ground or aircraft) measurements

The SIRS includes measurements from several different instruments to span the wavelength range from 0.1 to 2400 nm. The TIMED/SEE XPS provided 0.1-nm resolution data from 0.1 to 6.0 nm. An SDO/EVE prototype flew on a suborbital rocket on 14 April 2008 to acquire measurements from 6.0 to 105 nm that were binned down to 0.1-nm resolution for use in the reference spectra. Those results were scaled by the TIMED/SEE when included in the other WHI time periods. The TIMED/SEE EGS provided data from 105 to 116 nm. The 0.1-nm resolution SORCE/SOLSTICE provided data from 116 to 310 nm, and the SORCE/SIM provided low-resolution measurements (interpolated to 0.1 nm) from 310 to 2400 nm. For better absolute accuracy, all spectra were scaled to the SORCE/TIM TSI during the relevant periods. The reference spectra of all three WHI periods are available at http://lasp.colorado.edu/lisird/data/whi_ref_spectra and described by Woods et al. (2009). That of April 2008 is plotted in Fig. 27.

##### SAO2010 Chance–Kurucz reference spectrum

Chance and Kurucz (2010) combine high-accuracy space-based measurements with aircraft, balloon-, and ground-based measurements having higher spectral resolution to create the Smithsonian Astrophysical Observatory reference spectrum SAO2010. This spectrum is most notable for its extremely high spectral resolution, and significant effort went into determining spectral accuracies. The highest-resolution spectra are from the Fourier Transform Spectrometer at the McMath-Pierce Solar Telescope (see Sect. 2.5.1.9), which achieves spectral accuracies of 0.001 nm longward of 293.09 nm. Balloon-based measurements having spectral accuracies of 0.003 nm provide the shorter wavelengths not accessible from the ground. The SAO2010’s quoted < 5% radiometric accuracy is based on the ATLAS reference (see Sect. 2.5.1.2), so may inherit any inaccuracies that have since been found in that reference, to which the SAO2010 agrees to ~ 1%. The SAO2010 spans 200.07 to 1000.99 nm with a resolution of 0.04 nm.

**Fig. 28:**
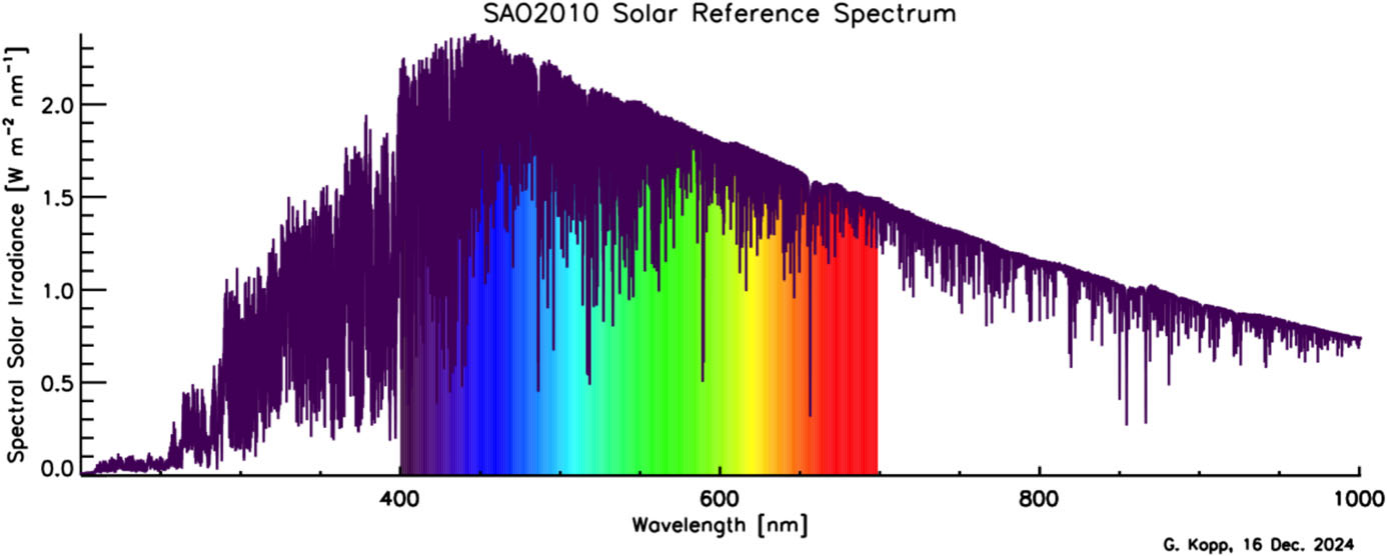
SAO2010 reference spectrum. Being a combination of high spectral resolution ground- and balloon-based measurements with radiometric space-based measurements, even on the scale shown here, the SAO2010 spectrum has higher spectral resolution than other reference spectra

Available from https://lweb.cfa.harvard.edu/atmosphere/links/sao2010.solref.converted, the SAO2010 reference spectrum is plotted in Fig. 28.

##### TSIS-1 HSRS

Version 2 of the TSIS–1 Hybrid Reference Spectrum (HSRS; Coddington et al. 2023), intended to be representative of the solar minimum between Solar Cycles 24 and 25, spans 202 to 2730 nm with 0.01 to ∼ 0.001-nm spectral resolution. Being another hybrid spectrum, the HSRS applies a wavelength-dependent scaling to high-resolution solar-line data to match lower-resolution but more radiometrically accurate SSI measurements, giving accuracies of 0.3% between 460 and 2365 nm and 1.3% outside that range (Coddington et al. 2021, 2023). This reference spectrum normalizes high-spectral-resolution datasets from the Air Force Geophysical Laboratory ultraviolet solar-irradiance balloon observations, the ground-based Quality Assurance of Spectral Ultraviolet Measurements In Europe Fourier transform spectrometer solar-irradiance observations, the Kitt Peak National Observatory solar-transmittance atlas, and the semi-empirical Solar Pseudo-Transmittance Spectrum (SPTS) atlas to match the lower-resolution, but higher-accuracy, TSIS-1/SIM spectrum. The Version 2 data correct a processing error in the prior version (Coddington et al. 2021) that affected the shortest wavelengths and updates to the 2020 SPTS (https://mark4sun.jpl.nasa.gov/toon/solar/solar_spectrum.html) solar lines (Toon 2014) that affect wavelengths > 743 nm. (n.b.: A 2024 version of these lines now exists.) The spectrum is then scaled to the TSI, which is known to much better accuracies (~ 0.015%) than the SSI measurements. The resulting HSRS Version 2 is shown in Fig. 2.

**Fig. 29:**
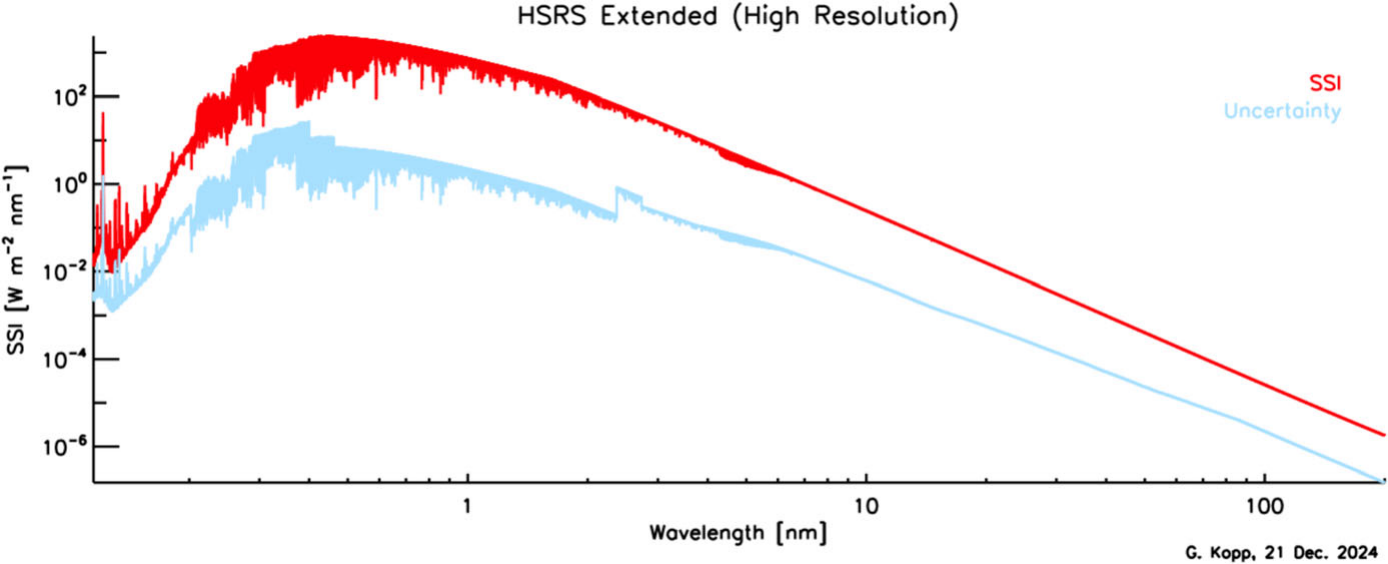
HSRS extended. The high-resolution version of the HSRS Extended spectrum (also known as the FS-HSRS, for “full spectrum”) is plotted along with the uncertainties

An extension of the version 2 HSRS extends the wavelength range from 115 nm to 200 µm. Longward of the SIM measurement range, the spectrum is extended by scaling the SPTS to a spectrum by Kurucz (1995) that is adjusted to match the SIM’s longest wavelength measurement. The Kurucz spectrum alone is used longward of 16.5 µm. Shortward of the SIM range, the SORCE/SOLSTICE results are used. The high-resolution version of this spectrum and its uncertainties are shown in Fig. 29.

##### Comparisons of reference spectra

**Fig. 30:**
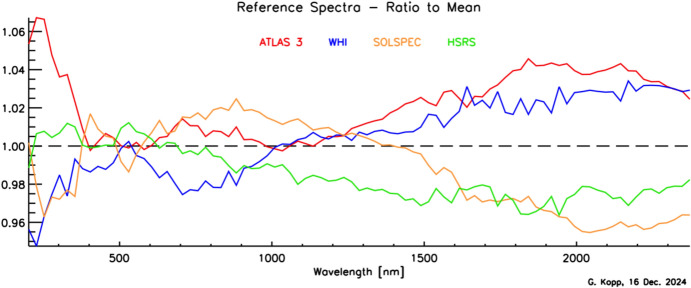
Comparisons of reference spectra. The four plotted reference spectra are ratioed to their mean. Differences are largest above 1500 nm, where the older (ATLAS-3 and WHI) spectra are up to ~ 8% higher than the TSIS-1/SIM-based HSRS

The four reference spectra that contiguously span 200 to 2400 nm, namely the ATLAS-3, WHI, SOLSPEC, and HSRS, are smoothed to 25-nm bins and plotted as ratios to their average in Fig. 30. The SAO2010 is not shown here because it only goes up to 1000 nm and, being based on the absolute accuracy of the ATLAS-3, would be within ~ 1% of those results. Likewise, the SCIAMACHY is not included because its spectral gaps make comparisons difficult. (See Hilbig et al. 2018 for such comparisons.) Agreement of the four plotted is best below 1300 nm but diverge significantly at longer wavelengths. The HSRS is the only one of these reference spectra based on the TSIS-1/SIM, which is considered to have the best absolute value of any SSI instrument over the visible and NIR. Although completely independent of the TSIS-1, the SOLSPEC reference spectrum most closely matches the HSRS. The ATLAS-3 and SORCE/SIM-based WHI are up to ~ 6% higher at the longer wavelengths and are likely erroneously high (see Richard et al. 2024).

A breakdown of the portions of the total flux due to different spectral regions for each of the four reference spectra covering 200 to 2400 nm is given in Table 4 along with the amount of the TSI’s 1361 W m^−2^ that they include.

##### NOAO atlases

Brief mention should be made of the high-resolution spectral atlases using the Fourier Transform Spectrometer at Kitt Peak’s McMath-Pierce Solar Telescope (Kurucz et al. 1984; Wallace et al. 1996, 2007). Attempts are made to remove telluric contamination from these ground-based observations. The primary benefit of these atlases comes from their spectral resolutions of ~ 500,000, which exceed any other ground- or space-based measurements. These are useful for details of spectral lines and identifying constituents and temperatures in the solar atmosphere, but they are not irradiance measurements, as they do not provide accurate spectral fluxes and some atlases are of specific active regions of the Sun rather than being disk integrated. However, normalized by more radiometrically accurate space-based measurements, they help give higher-resolution irradiances in a hybrid product.

#### Broad spectral range SSI time-series measurements

Temporally continuous measurements in the UV preceded broadband SSI measurements because the relative solar variabilities in those shorter wavelengths are much greater than in the visible and sensitivities of the Earth’s upper atmosphere to variabilities are more severe, as they affect radio communications and satellite drag. Because of the greater solar variabilities, climate-relevant measurement requirements at these wavelengths are less stringent than given in Table 2. McClintock et al. (2005b) estimate needed absolute accuracies of 5% and stabilities of 0.5% year^−1^ at wavelengths below 300 nm, where solar radiation is completely absorbed by the Earth’s atmosphere and is the dominant direct energy input to the atmosphere above about 15 km. Despite the more relaxed measurement requirements, ultraviolet measurements themselves remain difficult because of the lower overall spectral-irradiance signal levels and the increased susceptibility to instrument contamination, resulting in on-orbit degradation and poor measurement stability that hindered earlier instruments from achieving desired accuracies and stabilities. Rottman (1988) gives a detailed overview of the early UV and EUV measurements along with early-instrumentation techniques and difficulties.

Measurements of the SSI in the UV and shortwave visible (~ 120 to 400 nm) began with the NIMBUS-7 in 1978. In 2002, the wavelength range was extended through the visible and into the NIR with SCIAMACHY. Except for the UARS SOLar-STellar Irradiance Comparison Experiment (SOLSTICE), up until 2003 these instruments’ primary purpose was not acquiring radiometrically accurate SSI measurements. Instead, these were Earth-atmosphere-measuring instruments that used their solar-irradiance readings for on-orbit calibration of their throughput efficiencies, making long-term solar variability difficult to distinguish from instrument degradation. However, to the level of their intrinsic or correctible instrument stability, they do provide SSI-variability measurements that are reliable short-term. Additionally, their high spectral resolution can often resolve solar spectral lines that dedicated SSI instruments generally cannot, and since spectral-line core-to-wing ratios are relatively insensitive to instrument degradation, this enables diagnosing solar responses to various types of magnetic activity; see, for example, Criscuoli et al. (2023), who report on variations due to solar activity in time series of Ca II H&K, Mg II h&k, and Hα lines using data from SCIAMACHY, OMI, and GOME-2.

It was not until 2003 that radiometrically accurate broadband SSI measurements began with those being their primary purpose. The summaries below give a chronology of space-borne instruments acquiring time series of SSI measurements over broad spectral ranges.

##### SBUV and SBUV-2

The Solar Backscatter Ultraviolet (SBUV; Heath et al. 1975) operated on the NIMBUS-7 from October 1978 to July 1990. A second generation, the SBUV-2, maintained data continuity, beginning in January 1985 with successive flights on the NOAA-9, -11, and -14, -16, -17, -18, and -19 before being replaced with the newer OMPS (see Sect. 2.5.2.11) instruments. The SBUV instruments maintained design and operational consistency, all being nadir-viewing Ebert-Fastie double monochromators flying in Sun-synchronous orbits and providing daily SSI measurements from 160 to 406 nm with a 1.1-nm bandpass. Although the instruments’ primary purpose was Earth-atmospheric measurements, daily solar-irradiance measurements were made by deploying a solar-diffuser plate when crossing the day-night terminator and performing two consecutive spectral scans with the instrument. Intended as calibration measurements used to track changes in instrument sensitivity with time, these serendipitously provide a time series of SSI measurements across the instruments’ wavelength range and time durations to the level of stability of the diffuser scattering properties.

Pre-launch SBUV and SBUV-2 calibrations provide the instruments’ radiometric scales and accuracies. These include calibrations of wavelength scale, electronic gain ratio, nonlinearity, radiance and irradiance sensitivity (with NIST traceability), and solar-diffuser reflectivity and angular dependence. The SBUV-2 improvements over the NIMBUS-7/SBUV involved an on-board Hg-lamp calibration system to monitor long-term changes in the solar-diffuser’s reflectivity, as described by Weiss et al. (1991). There were no on-orbit radiometric calibration sources.

The NOAA-9 SBUV-2 (DeLand et al. 2004) observed from March 1985 to May 1997, providing the first SSI data from one solar minimum to the next. The new Hg-lamp calibration system didn’t work properly on this instrument, so substantial efforts were required to obtain a relatively stable solar-cycle-long data series. Following a method also used by the NIMBUS-7 SBUV, which lacked the SBUV-2’s on-board diffuser-monitoring system, exposure of the NOAA-9 SBUV-2’s diffuser was increased 10 × during the 1986 solar minimum, a time at which the solar irradiance was expected to be stable. This helped separate beginning-of-mission diffuser degradation from time-dependent instrument degradation. That, along with intermittent, coincident vicarious comparisons from eight Shuttle SBUV (SSBUV) flights acquiring solar-irradiance measurements during 1989 to 1996, allowed DeLand et al. (2004) to determine long-term NOAA-9 SBUV-2 corrections and derive SSI measurements over the solar cycle. They report spectrally dependent corrections of 20% at wavelengths from 190 to 210 nm decreasing to 3 to 7% at wavelengths longer than 300 nm and 2% at 395 to 400-nm wavelengths. The resulting NOAA-9 irradiance data for Solar Cycle 22 show an amplitude of approximately 9.3% at 200 to 205 nm, which is consistent with the NIMBUS-7 SBUV’s 8.3% for Cycle 21 and the UARS/SUSIM’s 10% for Cycle 23.

**Fig. 31:**
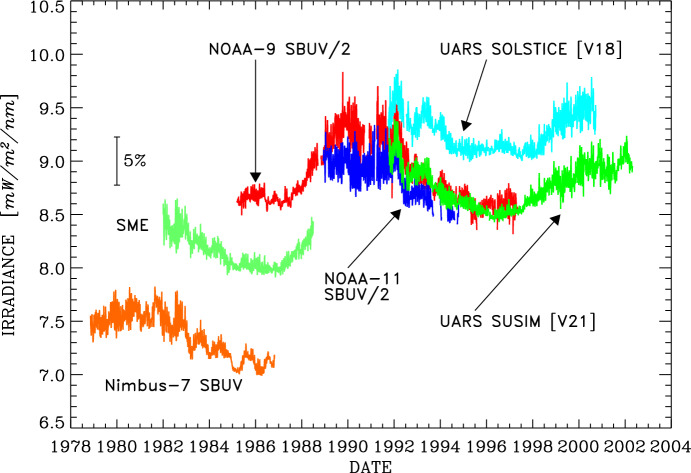
SSI measurements from 200 to 205 nm from the earlier SBUV-instrument series along with those from other instruments during the era. Image reproduced with permission from DeLand et al. (2004), copyright by AGU

The results from the early SBUV instruments along with other concurrent instruments are shown in Fig. 31.

##### SME/UV

The Solar Mesosphere Explorer (SME), another satellite designed to measure ozone in the Earth’s atmosphere, measured the SSI from 120 to 305 nm with 0.7-nm resolution using a small UV spectrometer (Rottman et al. 1982). After launch into a nearly circular Sun-synchronous orbit on 6 October 1981, the NASA- and JPL-managed mission operated from the Solar Cycle 21 maximum to the Cycle 21-to-22 minimum. The solar instrument on board was another Ebert-Fastie spectrometer measuring sunlight scattered from a diffuser. Separate spectral channels covered 120 to 200 nm and 174 to 305 nm, each with photomultiplier tubes having sensitivity calibrations traceable to the National Bureau of Standards (NBS; now NIST). Estimated intrinsic on-orbit uncertainties were ± 30%. Degradation tracking of the main diffuser was enabled by additional diffusers that were duty cycled to have lower solar exposure; little to none was observed in the short-wavelength channel with  < 1% observed in the long-wavelength channel over a 700-day period. The instrument did not have on-orbit radiometric calibration sources.

The instrument acquired a solar measurement at an individual 0.25–nm-stepped wavelength every 12 s as the spacecraft spun, and a daily spectrum is produced using averages from that day’s 24-h period. Data are available from 11 October 1981 to 13 April 1989 from 115.5 to 302.5 nm in 1-nm bins. Mid-wavelength data are shown in Fig. 31.

Six suborbital rocket underflights improved stability, requiring ~ 15% corrections at Lyman-*a* and up to 6% at longer wavelengths over the mission’s 7-year timeframe (DeLand et al. 2004). Measurements were normalized to an underflight on 17 May 1982, giving accuracies of ± 15%, as described by Mount and Rottman (1983). Rottman (1988) reported the long-term stability to be no better than 2% per year at the shortest wavelengths (near Lyman-*a*) and 1% per year at longer wavelengths. With these uncertainties, he suggested solar-cycle variability of ~ 5% at 200 and 3% at 300 nm, which are lower than prior estimated variabilities and have  ± 100% uncertainties, so were indistinguishable from no variability.

##### UARS/SOLSTICE

The SOLSTICE (Rottman et al. 1993) was one of ten instruments aboard the UARS spacecraft, launched on 12 September 1991. The SOLSTICE measurement goals (requirements) were to acquire daily SSI measurements from 115 to 420 nm having spectral resolution λ/Δλ ≈ 1000 (0.1 to 0.2 nm), uncertainties  < 5% (10%), and stabilities  < 1% (2%) over the planned 18-month mission (Woods et al. 1993). The instrument operated from October 1991 until September 2001. This modified Monk-Gillieson spectrometer consisted of three channels having similar optical designs based on a rotating grating scanning the spectral region across that channel’s photomultiplier-tube detector. The wavelengths covered by each channel were 115 to 190 nm, 170 to 320 nm, and 280 to 420 nm, with the overlaps helping determine channel offsets and provide data validation. The channels were stacked within a common housing, differing from each other in their mirror coatings, photocathodes, planar diffraction gratings, and windows of the photon-counting photomultiplier-tube detectors. Pre-flight calibrations were conducted at the NIST’s Synchrotron Ultraviolet Radiation Facility (SURF), which also provided preliminary estimates of expected degradation due to UV exposure.

Although the SOLSTICE had no on-orbit radiometric calibration sources, flight measurement stabilities were achieved by comparisons to an ensemble of stable, bright, early-type (O and B on the main-sequence) stars to monitor instrument degradation. These stars were chosen for (1) their blue-weighted spectra that favor the SOLSTICE spectral range, where they are 5 to 8 orders of magnitude less bright than the Sun (rather than 10^12^, as in the visible), and (2) their inherent low variability, which is estimated to be  < 1% in the UV over 10^4^ years. This large dynamic range between the solar and stellar irradiances is accommodated by a combination of interchangeable instrument entrance apertures, whereby solar measurements are attenuated by 10^4^ relative to stellar measurements; larger stellar bandpasses, giving a factor of 10; and integration times giving a factor of 10^3^. On-orbit comparisons to this ensemble of stars were possible to a precision of ~ 3% over the mission.

In addition to a daily solar spectrum across the SOLSTICE’s spectral range, the instrument provided daily 121.6-nm Lyman-α emission products and a 279.6-nm Magnesium (Mg II) emission core/wing ratio indicative of solar faculae. Being a ratio, this core-to-wing ratio is largely free of degradation. Nevertheless, other complications, such as wavelength-dependent sensitivity changes causing drifts, offsets, and bandpass differences between instruments, can affect the long-term Mg II record (see DeLand and Cebula 1993; Cebula and DeLand 1998; Viereck et al. 2004; Snow et al. 2014).

Mid-wavelength UARS/SOLSTICE data are plotted in Fig. 31. The SOLSTICE data are available from 3 October 1991 to 30 September 2001 via the NASA GSFC DISC at https://daac.gsfc.nasa.gov/datasets/UARSO3BS_018/summary.

##### UARS/SUSIM

The UARS Solar Ultraviolet Spectral Irradiance Monitor (SUSIM; Brueckner et al. 1993) covers the wavelength range 110 to 415 nm, so acquires SSI measurements similar to those of the UARS/SOLSTICE but uses a very different calibration scheme. This SUSIM is a long-term UV solar-irradiance-monitoring successor to that flown on Spacelab flights in 1984 and 1985. Those predecessor Spacelab instruments demonstrated a marked improvement in absolute accuracies from prior solar UV measurements as well as the importance of pre-launch cleanliness for reducing degradation, both of which helped support and improve the redesigned UARS/SUSIM follow-on. Unlike the preceding SBUV and SME and the contemporary SOLSTICE, the SUSIM included an onboard UV irradiance standard enabling radiometric calibrations.

The UARS/SUSIM consists of two double-dispersion Wadsworth spectrometers, one of which views the Sun and the other of which monitors on-board calibration sources, which can then transfer radiometric calibrations to the solar-viewing spectrometer when the reference views the Sun. Each spectrometer has four optical channels for a total of eight optical paths (rather than the two of the earlier Spacelab instruments). Those four channels are determined by selection of one of four identical gratings on a rotating turret. The selected grating disperses incident light onto a fixed secondary grating in each dual-grating spectrometer. Its rotation angle determines the wavelength observed with 0.02 nm-per-step resolution. The multiple-channel design allows for duty cycling of the gratings to track exposure-dependent degradation and backup as the more-used gratings degrade. Each of the dual spectrometers had three entrance and three exit slits to determine spectral resolution, giving options of 0.15, 1, and 5 nm. Five photodiodes and two photon-counting detectors are common to both spectrometers to read the output signals. Two filter wheels, one in front of the entrance slit and one after the exit slit, contain six filters plus an open position. Any of those can be placed inline in either spectrometer. Four deuterium-lamp calibration sources, any of which can be moved in front of either of the two spectrometers, were included to reduce usage-dependent lamp degradation.

All optical elements except the second (fixed) grating in each spectrometer can be moved in or out of the optical path. This flexibility in channel (grating) selection, output detector, slits, filters, and calibration sources enables multiple optical-component combinations from which degradation of individual elements can be isolated. Degradation of optics is minimized by having only one optical surface, namely the selected grating in the four-channel rotating turret, exposed to the full incoming solar irradiance, with the expectation that most of the degradation would occur on this optic; and even it is partially protected by the selected filter before the entrance slit. Cleanliness drove procedural requirements for both the instrument and spacecraft to reduce on-orbit degradation caused by solar-UV-caused polymerization of contaminants.

The threshold measurement requirements were spectral resolutions between 0.5 and 5 nm; accuracies of 10% below 285 nm, 3% from 285 to 330 nm, and 1% at longer wavelengths; and precisions of 5%, 1%, and 0.5% for the same ranges. The most-accurate preflight calibrations primarily relied on NIST’s SURF and FEL tungsten irradiance lamps, with the SURF spanning the entire SUSIM wavelength range. Wavelength-scale uncertainties limited actual accuracies to a uniform ~ 5.1% across the SUSIM’s spectral region. While requirements were not met at the longer wavelengths, the more important shorter-wavelength precision was achieved. The imposed cleanliness requirements were beneficial: the SUSIM degraded initially by 2% per day at Lyman-*a* (which decreased to 0.31% per day after a year), while previous spacecraft UV spectrometers had shown decreases of 10^3^ in the first few days of operation.

The SUSIM acquired daily SSI measurements from 115 to 410 nm at 1-nm and 5-nm resolutions and weekly scans at high resolution (0.15 nm). Mid-wavelength UARS/SUSIM data are included in Fig. 31. Daily SSI data with 1-nm resolution are available from the NASA Open Data Portal at https://data.nasa.gov/dataset/UARS-Solar-Ultraviolet-Spectral-Irradiance-Monitor/5vv3-gnfi/about_data.

##### GOME and GOME-2

The Second European Remote Sensing Satellite (ERS-2) launched in April 1995 carrying the Global Ozone Monitoring Experiment (GOME; Burrows et al. 1999). The GOME, a small-scale version of the SCIAMACHY planned for the ENVISAT-1, was intended to initiate global trace-gas measurements as soon as possible after increased appreciation of the negative effects of Antarctic ozone depletion. Those trace-gas retrievals required observations over the spectral range 240 to 790 nm with 0.2 to 0.4-nm resolution. Measurement objectives also included knowing the incoming solar irradiance and monitoring solar UV irradiance variability over the solar cycle, so the GOME became the first SSI-measuring instrument to span the visible range with lengthy time-series measurements. Data are available from June 1995 until July 2011, when the mission was taken out of service.

The instrument is a four-channel grating-based spectrometer with prisms providing pre-dispersion. They and a dichroic beamsplitter provide spectral separations into the four channels of 240 to 315 nm, 315 to 405 nm, 405 to 610 nm, and 595 to 790 nm. A sanded aluminum plate with a Cr/Al coating acts as a solar diffusor to enable SSI-calibration measurements. 1024-element linear reticon arrays acquire the entire spectrum in each of the four channels. Unlike the SBUVs, SME, SOLSTICE, and SUSIM, the GOME thus acquires its entire spectrum simultaneously rather than building up a scan across the spectrum piecemeal over what can be multiple orbits spread throughout a day. The SSI measurements are acquired in a 50-s period once a day when the ERS-2 crosses the day/night terminator in its Sun-synchronous, near-polar orbit.

Preflight GOME calibrations were done using a FEL lamp traceable to a NIST reference standard and are estimated to be 1.5% (*k* = 1) at 240 nm and 1.1% from 300 to 400 nm (Weber et al. 1998). Relying on the SSI measurements as a relative indicator of internal-efficiency degradation once on orbit, the GOME has no internal means of absolute radiometric calibration for long-term accuracy maintenance, although monthly measurements with an internal hollow-cathode calibration lamp monitor solar-diffusor degradation and provide wavelength calibrations. Weber et al. (1998) discuss validations of the GOME’s SSI measurements via intercomparisons with other instruments and solar-activity proxies. They estimate overall SSI calibration uncertainty to be better than 5% with day-to-day precision of  < 1% after correcting for UV degradation and temperature-dependent etaloning on the detectors. They use these corrected solar measurements to create Mg II and Ca II K proxies, and they additionally discuss solar-cycle forcing of Earth-atmospheric ozone, since the GOME measures both.

ERS-2 GOME data are available from https://earth.esa.int/eogateway/catalog/ers-2-gome-spectral-product-l1.

First launched on the European Organisation for the Exploitation of Meteorological Satellites (EUMETSAT) polar-orbiting Sun-synchronous MetOp-A satellite in October 2006, the redesigned GOME-2 series measures from 240 to 790 nm with 0.24 to 0.53 nm resolution (Callies et al. 2000; Munro et al. 2016). This instrument was followed by others on the MetOp-B (September 2012) and MetOp-C (November 2018). The GOME-2’s optical design is nearly identical to the original GOME with changes primarily including an improved Polarization Monitoring Unit, holographic gratings with lower polarization sensitivity, and the inclusion of a quartz tungsten halogen lamp for broadband calibrations helpful in monitoring the detector-surface etaloning that plagued the GOME-1. More substantial changes to the instrument involved the electronics and data handling systems, as needed for integration with the different space platform and its solar-viewing opportunities. Munro et al. (2016) describe the ground-based and in-orbit calibration improvements, particularly of the solar diffuser’s bidirectional scattering distribution function, over those of the GOME-1.

As with the GOME-1, the GOME-2 acquired a solar-irradiance spectral calibration once per day. The GOME-2 also produced a daily Mg II product. GOME-2A and -2B data are available from 31 March 2007 to 30 July 2020 (see https://navigator.eumetsat.int/product/EO:EUM:DAT:0533), although the GOME-2 SSI data are difficult to access, since they are not a primary data product.

##### SCIAMACHY

One of ten remote-sensing instruments on the ESA’s Envisat, the SCIAMACHY further expanded spectral coverage, measuring the SSI from 240 to 2380 nm with 0.2 to 1.5 nm spectral resolution (Bovensmann et al. 1999). This is another Earth-atmospheric trace-gas imaging spectrometer that acquires a daily 30-s solar-irradiance measurement to monitor internal instrument-sensitivity changes on orbit. It operated from August 2002 to April 2012.

Other than the scan mirrors to select regions of study, all spectrometer optics are fixed. A predispersing prism and gratings reduce stray light similarly to a double-spectrometer design. The grating-based spectrometer records spectra contiguously and simultaneously from 240 to 1750 nm with additional contiguous windows from 1940 to 2040 nm and 2265 to 2380 nm. These spectral regions are separated into eight optical channels, each with its own grating, optics, and 1024-element cooled linear diode-array detector. Dichroics separate the light for channels 3 through 8, which collectively measure all light longward of 405 nm. Spectral stabilities are maintained to  < 0.005 nm for most channels by stringent thermal control of the instrument. As on GOME-2, polarization measurement devices are included, although these are not relevant for solar-irradiance measurements.

Similarly to GOME-2, a Pt/Cr/Ne hollow-cathode tube provides on-orbit spectral calibrations while a tungsten-halogen lamp (of 3000-K equivalent blackbody temperature) provides relative broadband radiometric calibrations. The radiometric accuracies of solar-irradiance measurements depend on the pre-launch calibrations using a NIST-calibrated FEL lamp and integrating spheres plus the on-orbit stability of an onboard diffuser. Hilbig et al. (2018) give lower-limit solar-irradiance uncertainties from 6.5% at 240 nm to 2.9% at 500 nm and 5.1% at 2400 nm (*k* = 2). They suggest an additional uncertainty of up to 13% due to a stray-light problem longward of 800 nm.

**Fig. 32:**
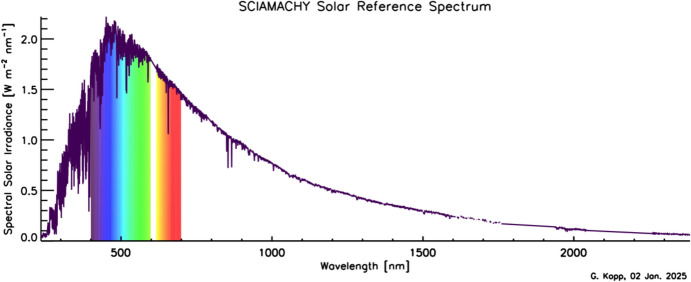
SCIAMACHY SSI reference spectrum from 27 February 2003. The spectrum is more accurately termed a beginning-of-life “baseline” spectrum from which relative degradation corrections are tracked, as it has several spectral gaps. (data from https://www.iup.uni-bremen.de/UVSAT/data/solarreference/) (see Hilbig et al. 2018)

To improve on-orbit stabilities, Hilbig et al. (2020) iteratively degradation-correct the SCIAMACHY SSI measurements using the onboard tungsten-halogen lamp as well as fits to solar proxies to allow for long-term solar variability. Their baseline reference day, shown in Fig. 32 and described by Hilbig et al. (2018), is 27 February 2003, chosen early in the mission with the intent of being prior to significant degradation. Their degradation-corrected SSI results relative to this baseline still show residual instrumental issues when compared to cotemporaneous spaceflight measurements from other instruments. The authors summarize that their applied corrections are sufficient for the SCIAMACHY’s intended atmospheric applications and for timescales of solar rotations but not for studies of long-term (i.e., solar-cycle) variability.

**Fig. 33:**
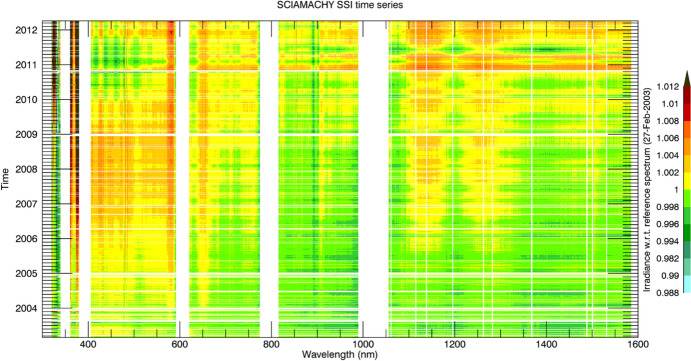
SCIAMACHY SSI time series. (from https://www.iup.uni-bremen.de/UVSAT/data/solartimeseries/, based on Hilbig et al. 2020)

**Fig. 34:**
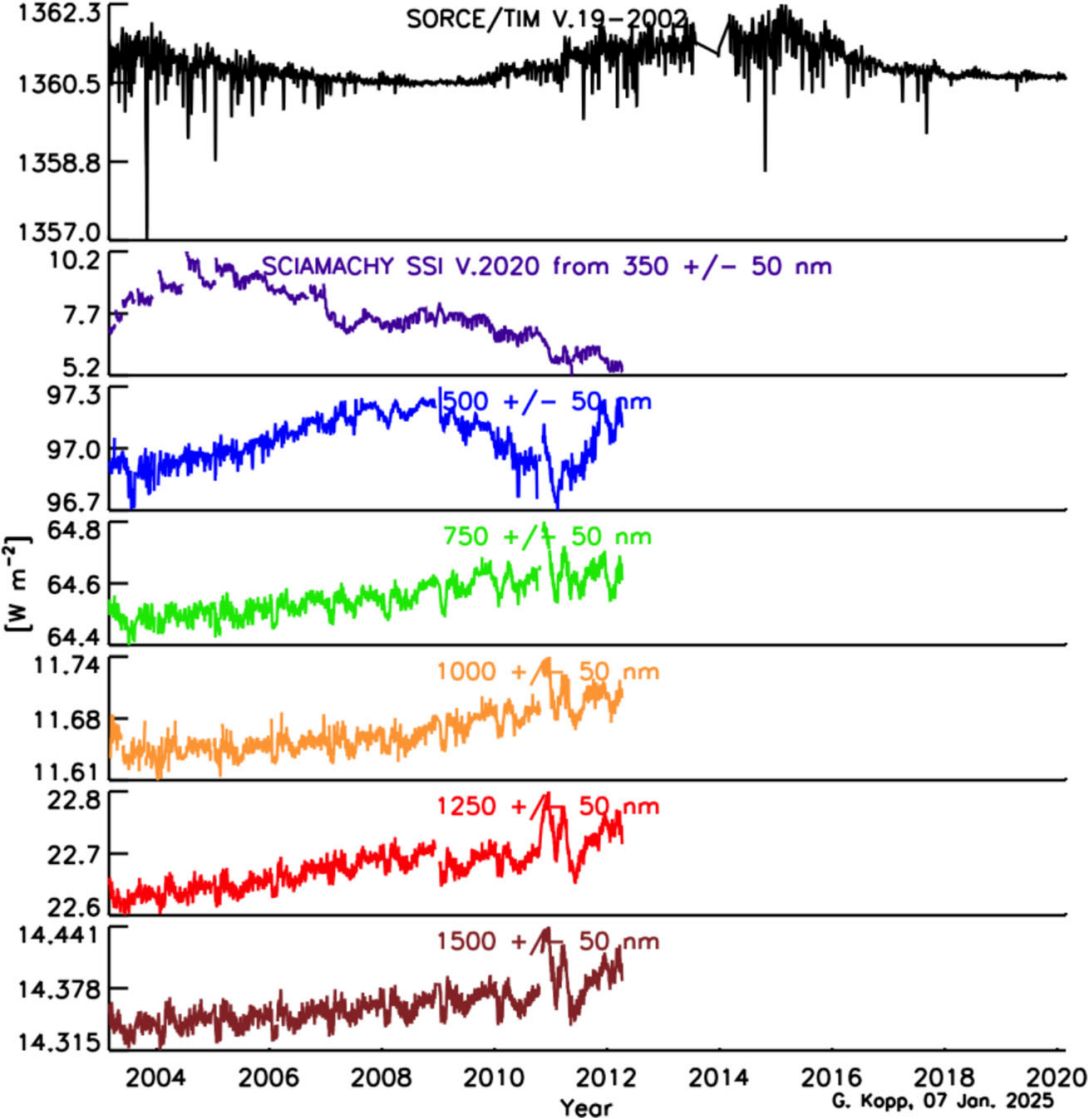
SORCE TSI and binned SCIAMACHY SSI. While SCIAMACHY can provide useful short-term solar-variability data, the longer-term (solar-cycle) results are influenced by residual instrument-stability effects. (See Hilbig et al. 2020.)

The SCIAMACHY solar-irradiance data were acquired daily and are easily accessible. Time-series data are available from https://www.iup.uni-bremen.de/UVSAT/data/solartimeseries/ and a reference spectrum from https://www.iup.uni-bremen.de/UVSAT/data/solarreference/ (Hilbig et al. 2018). These data still have limitations in degradation corrections and currently only span 320 to 1585 nm with spectral gaps at the SCIAMACHY spectral channel boundaries as well as near 350 nm (see Fig. 33). Spectrally binned time series at different wavelengths are plotted in Fig. 34. As Hilbig et al. (2020) warn, the data are not sufficiently stable for solar-variability studies on solar-cycle timescales but are useable on short-term timescales.

##### SORCE/SOLSTICE

Two SOLSTICE instruments were flown on the SORCE mission, continuing the UARS/SOLSTICE measurements (albeit after a temporal data gap) from 2003 until 2020 (McClintock et al. 2005a). The on-orbit operations are similar to those of the UARS/SOLSTICE, using an ensemble of main-sequence B and A stars to track instrument degradation. Based on the UARS observations of those stars, 18 were selected for their brightness and stability for the SORCE/SOLSTICE.

These two newer SOLSTICEs spanned 115 to 320 nm with 1-nm resolution and a 6-h measurement cadence. This spectral range did not extend into the visible, as the UARS/SOLSTICE did, since the SORCE included the SIM that overlapped the UARS/SOLSTICE’s longer wavelengths and scanned contiguously into the NIR; thus, only the two shorter-wavelength channels from the UARS design were needed. Measurement requirements for absolute accuracies of 5% and stabilities of 0.5% year^−1^, as given by McClintock et al. (2005b), are more stringent than the those of the UARS/SOLSTICE. These were largely achieved by improvements to the SURF calibrations and instrument modifications including real-time pointing knowledge and more uniform detectors to reduce field-of-view corrections, a grating-position encoder to improve wavelength scales, redundant detectors and a corresponding 1.5 × focal-length increase to better track instrument sensitivities, and a composite optical bench for better wavelength stability.

With these changes, the FUV spectra are acquired using a 2265-step scan covering 115 to 190 nm using 1-s integrations, while MUV spectra cover 150 to 320 nm in 4975 steps using 0.5-s integrations. Spectra collected during each 6-h interval are averaged and binned from their native  < 0.1 nm to 1 nm. The SORCE/SOLSTICE achieved 1.2 to 6% absolute accuracy with a relative stability uncertainty of 0.60 to 1.04% year^−1^ (Snow et al. 2022). Daily data of 1- and 0.1-nm resolutions as well as a specific Lyman-*a* product are available from https://lasp.colorado.edu/sorce/data/ssi-data/

##### SORCE/SIM

The SORCE mission’s Spectral Irradiance Monitor (SIM) commenced a record of daily SSI-measurements spanning the visible and NIR with intendedly sufficient radiometric accuracies and on-orbit stabilities to discern solar-cycle variability. This instrument, described by Harder et al. (2005a, b), is a dual-channel ESR-based prism spectrometer spanning 240 to 2416 nm with between 1 and 34-nm spectral resolution. (The TSIS-1/SIM has similar resolution; see Fig. 40.) A small ESR in each of the two SIM channels provides stable radiometric optical-power measurements. This detector is used to calibrate the instrument’s photodiodes, which acquire spectral scans rapidly but lack radiometric accuracies and long-term stabilities. Different detectors cover different spectral ranges: silicon photodiodes cover the “UV” (205 to 308 nm) and the “visible” (310 to 900 nm), an InGaAs photodiode covers the “IR” (900 to 1550 nm), and the ESRs cover all longer wavelengths. A NIST-calibrated entrance slit defines the area over which sunlight is collected. A Féry prism is the only optical element in each channel, reducing the number of optical surfaces that can degrade with solar exposure. Rotating the prism provides spectral scanning. A CCD monitors the prism-rotation angle to determine the wavelength scale.

The two SIM channels, optically stacked such that a common prism mount rotates both prisms together, have solar-observation rates duty cycled in a 5:1 ratio. The released SIM data assume each channel degrades similarly as a function of solar-exposure time, wavelength, and prism angle. Prism degradation is assumed to be exponential with time.

Radiometric accuracy was estimated at ~ 1% (Harder et al. 2005a, b). Stability uncertainties, however, were not well quantified, particularly on yearly and longer timescales, primarily due to the difficulties determining the degradation functions between the two channels. The published data are particularly uncertain during the declining phase of Solar Cycle 23, where the SIM data differ significantly from solar models, solar proxies such as Mg II and F_10_, and independent solar-irradiance measurements. Since the instrument degradation is as large as 40% in the UV during this beginning portion of the mission, corrections to effects of this magnitude require very accurate knowledge of the actual degradation to achieve desired stabilities.

**Fig. 35:**
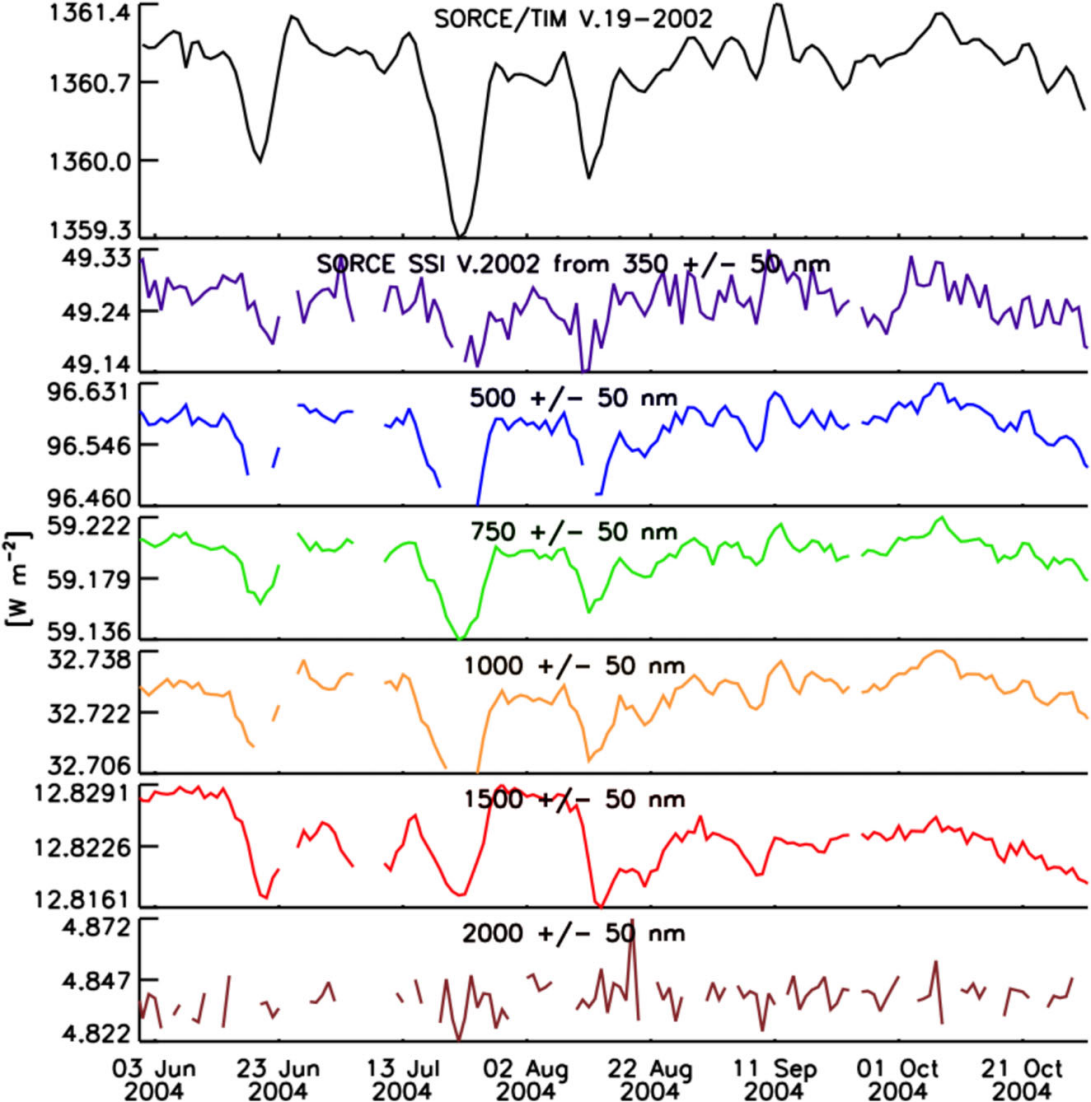
SORCE TSI and binned SSI Over a 5-month period. The SSI integrated over 100-nm bins about the shown center wavelengths generally follows the TSI over this 5-month period over which the SIM is relatively stable. The longest wavelengths were not scanned regularly, so have gaps

Figure 35 compares the TSI to various spectrally integrated bands of SIM SSI data on short-term timescales. Over the 5-month period shown, the SIM instrument is relatively stable, so there are good correlations between the TSI and SSI. The larger decreases in the TSI are due to sunspots and are also evident in the SSI in the visible and NIR. Sunspots have less contrast in the UV, and those wavelengths respond mainly to faculae, which cause brightenings. At the longest shown wavelength, the Sun has lower signal, the instrument has higher noise, and data were not acquired every day.

**Fig. 36:**
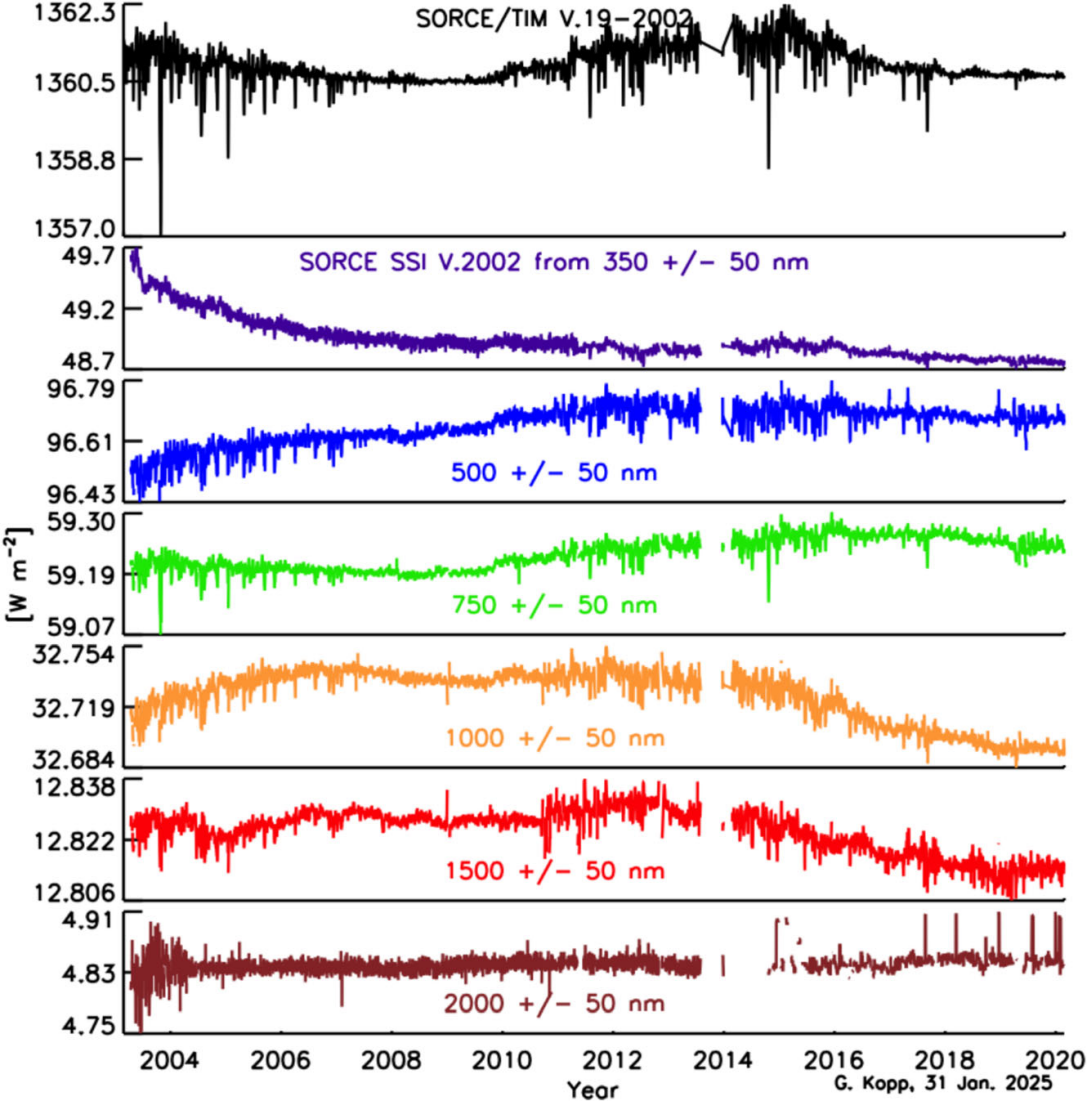
SORCE TSI and binned SSI. The SORCE TSI (top plot) shows solar-cycle variability over the 17-year mission duration. The binned SORCE SSI measurements (bottom 6 plots) lack the stability to definitively show realistic solar-cycle responses. The apparent trends are likely from uncorrected instrument artifacts

Although the SIM-measurement stabilities were much better than any prior such measurements over long-term (i.e., solar-cycle) timescales, the desired levels of accuracy and stability were not achieved in the visible and NIR (Skupin et al. 2005; Harder et al. 2009). Similarly, at shorter wavelengths, such as in the 250 to 300-nm range, the SORCE/SIM indicates nearly 3 times greater variability during the declining phase of Solar Cycle 23 (which peaked in 2001) than other instruments (or models) spanning the ultraviolet (Lean and DeLand 2012; Yeo et al. 2015). The instrument seemed to stabilize heading into Solar Cycle 24, the hypothesis being that the initial SIM measurements, particularly in the UV, were plagued by degradation due to outgassing of internal contaminants. Figure 36 shows the measured TSI compared to several SIM-measured SSI spectral bands showing long-term trends that are inconsistent with the TSI, with each other, and with the solar cycle.

While these differing trends do not necessarily indicate measurement instabilities, that scenario is much more likely than a solar-physics-based explanation for such trends. The cause of these inconsistencies is likely due to the degradation tracking ability of the dual-channel instrument as well as contamination from initial on-orbit instrument outgassing. The SORCE/SIM SSI-data, available from 14 April 2003 to 25 February 2020 via https://lasp.colorado.edu/sorce/data/ssi-data, are generally best used either over time ranges of limited duration, where such potential instrument-artifacts are less apparent, or after applying a short-pass detrending filter to the data.

**Fig. 37:**
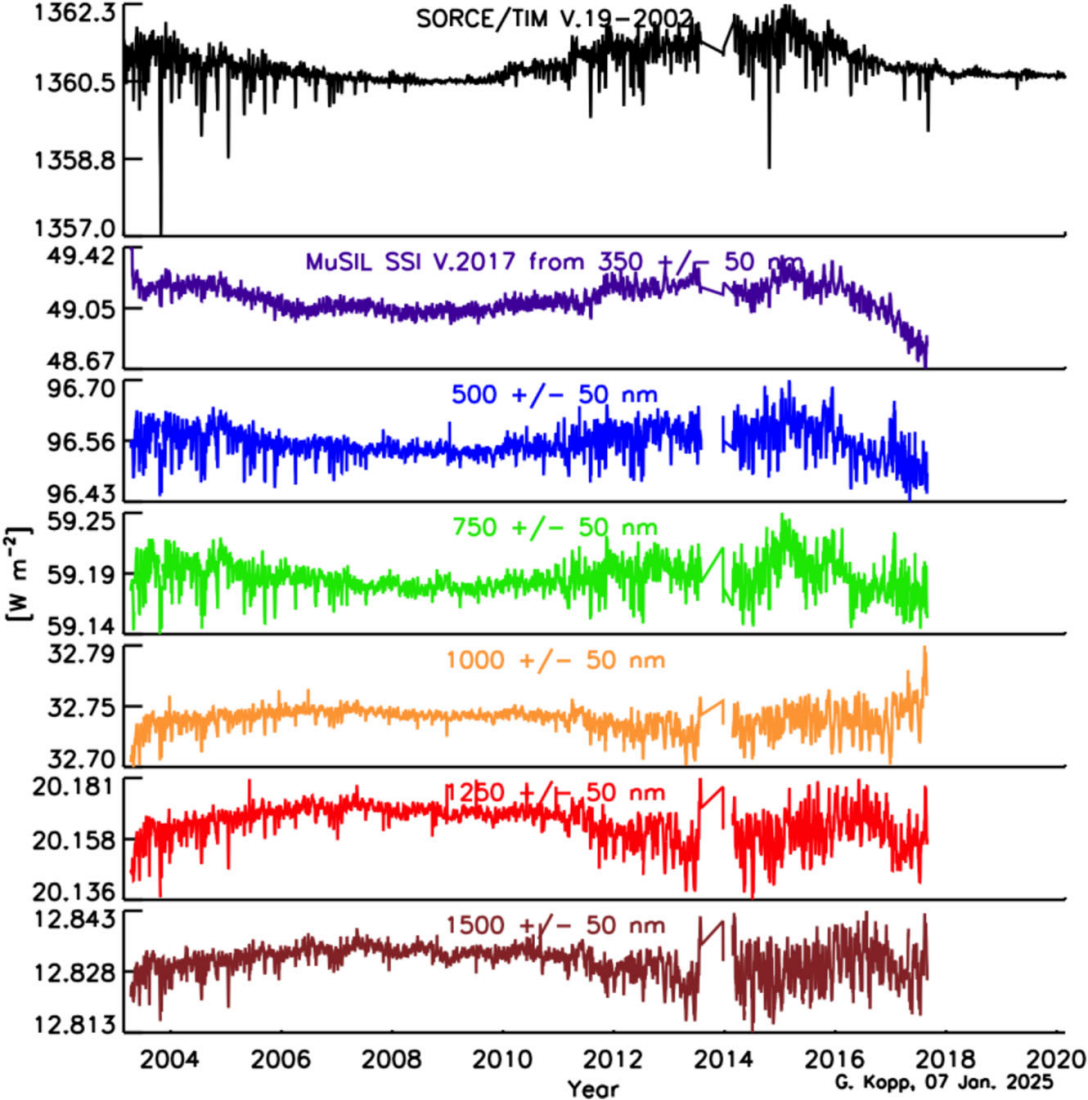
SORCE TSI and binned MuSIL SSI. The MuSIL approach corrects for some of the long-term SORCE/SIM trends by forcing agreement with solar proxies. These data suggest an out-of-phase solar-cycle dependence in the NIR

**Fig. 38:**
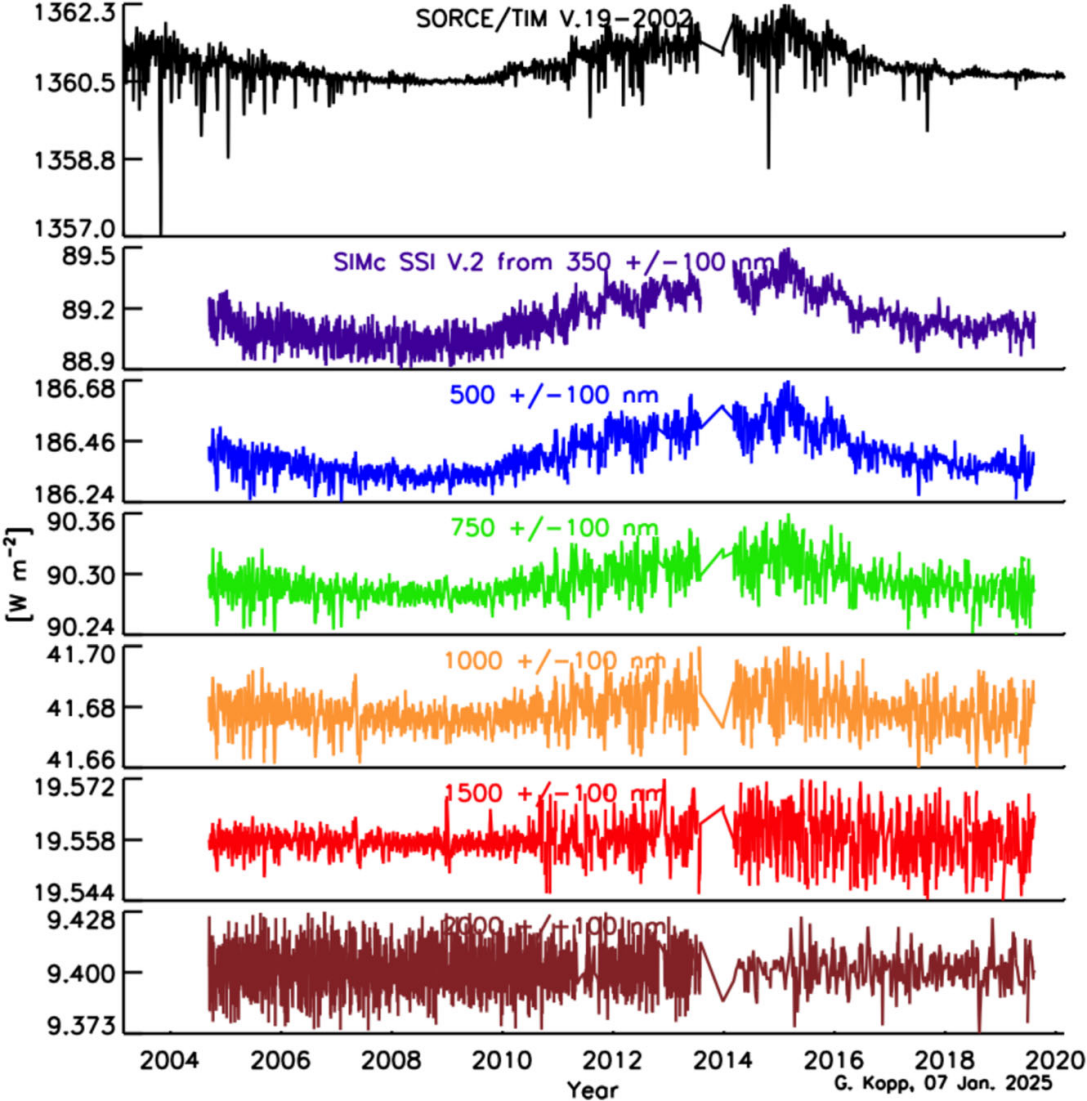
SORCE TSI and SIMc V.2 SSI. The SIMc corrects the long-term SORCE/SIM data by applying constraints to follow the TSI. These spectral bins are larger than in prior plots since the SIMc wavelengths are reported on sparser scales than the instrument data

Alternatively, adjusted data are available from various authors. These modified values rely on data other than those of the SIM measurements, such as solar proxies or the TSI, and thus are dependent on solar-variability assumptions. Some of these are summarized here:

- *MuSIL*: Woods et al. (2018) use the novel Multiple Same-Irradiance-Level (MuSIL) technique to attempt to discern and adjust for the long-term SORCE/SIM instrument effects. This method assumes there are no solar variations with periods longer than a solar cycle. It attributes any such variations in measurements, as indicated by differences in the measurements from times of identical solar activity (as determined by multiple solar proxies) to uncorrected instrument artifacts. This approach can introduce artifacts when spanning two very different solar cycles, such as Cycles 23 and 24 (to which it is applied), and when using the solar proxies for all wavelength regions, since, in reality, some of the proxies are inappropriate for some wavelength regions, such as the UV or NIR. The MuSIL-corrected SSI cover 240 to 1600 nm from May 2003 to August 2017. Since the technique relies on finding times of identical solar activity, it can have difficulties discerning instrument trends near the endpoints of the time range over which it is applied; in the case of the SORCE/SIM, these are likely prior to May 2004 and after January 2018 (Mauceri et al. 2020). Figure 37 shows the SORCE-measured TSI compared to MuSIL-reported SSI spectral bands.
- *SIMc V.1*: Mauceri et al. (2018) define a wavelength- and time-dependent prism- and photodiode-degradation model to improve the long-term SIM results with the constraints that they (1) agree with the TSI when spectrally integrated and (2) have spectrally smooth degradation corrections over 20-nm regions. This approach makes no assumptions about exponential degradation with time, nor does it assume both SIM channels behave similarly; they are treated independently. The “SIM-constrained” (“SIMc”) SSI results are adjusted to the WHI reference spectrum (see Sect. 2.5.1.5). These results agree with solar models (SATIRE-S and NRLSSI2) and the TSI, and they disagree significantly from the published SIM data (V.23 at the time), instead giving UV and visible variations that are in-phase with the solar cycle. The latter is not surprising, given that the technique forces consistency with the long-term TSI behavior.
- *SIMc V.2*: Version 2 of these corrections (Mauceri et al. 2020) covers a larger wavelength range, going from 205 to 2375 nm, and interpolates measurements to fill observational data gaps for wavelengths between 1550 and 2375 nm. The V.2 corrections extend from September 2004 to August 2019 and adjust to the TSIS-1/SIM’s absolute scale. This V.2 is a likely improvement, albeit a small one, over V.1, and has similar advantages and issues. One finding of this update is that the latest SIM data at the time of publication (V.25) underestimates instrument degradation below 400 nm, which is an important spectral region for Earth-climate studies. The authors recommend their SIMc V.2 SSI results over the released SIM data. Figure 38 shows the SORCE-measured TSI and select SIMc V.2 SSI spectral bands.

The SORCE/SIM measurements initiated a new spectral data record with better long-term stability than prior instruments and provide the longest duration of broad spectral coverage SSI records available. While not yet achieving the accuracy or stability requirements for long-term climate studies, the visible and NIR SSI data have proven valuable for studies of short-term solar-variability effects on the Earth’s atmosphere (Gray et al. 2010; Haigh 2011; Haigh et al. 2012).

##### Aura/OMI

The NASA Aura satellite’s Ozone Monitoring Instrument (OMI) was launched on 15 July 2004 into a Sun-synchronous polar orbit and continues to operate. A modernized and more complex successor to the GOME and SCIAMACHY, the instrument includes three overlapping grating-based spectral channels, which span 270 to 314 nm, 306 to 380 nm, and 350 to 500 nm with respective resolutions of 0.42, 0.45, and 0.63 nm (Levelt et al. 2006). As with the SBUV, GOME, and SCIAMACHY, this GOME-heritage Earth-monitoring instrument views the Sun via a diffuser for on-orbit calibration purposes once per day. Three such diffusers allow for duty cycling to track diffuser degradation. Pre-launch measurements provide radiometric calibrations, spectral knowledge, diffuser characteristics, and detector performance. Two major changes over the GOME and SCIAMACHY include the use of 2-D 576 × 780-pixel (spatial x spectral) CCD detectors with accompanying optics for spatial/spectral measurements and a polarization scrambler; although neither change is particularly relevant for solar-irradiance measurements. A tungsten-halogen lamp monitors on-orbit radiometric degradation as well as non-linearities and pixel-to-pixel variations of the CCDs.

Marchenko et al. (2016) summarize the instrument’s performance, stating an accuracy better than 4% over most of the spectral region. The degradation rate in the visible is 0.2 to 0.5% year^−1^, giving systematics that are larger than the solar variability on solar-cycle timescales but appropriate for shorter (i.e., solar-rotational) timescales. Marchenko and DeLand (2014) suggest that the short-term (weeks) UV variability scaling to Mg II should match the long-term (years) changes, in agreement with earlier conclusions by DeLand and Cebula (2012). Even with the long-term instrument-stability limitations, Marchenko et al. (2016) were able to use the OMI data to refute the high Solar Cycle 23 variability reported by the SORCE/SIM in the 290 to 320 nm range (Harder et al. 2009), with OMI more consistent with other indicators of variability over this time and wavelength range.

By the time of the Aura launch, there were dedicated SSI-measuring instruments with better accuracies. The niche of the OMI is in short-term solar-variability measurements, particularly in the UV, and in providing high spectral-resolution measurements of solar lines over a 2-decade time span.

Daily and high-cadence (98-min) OMI solar irradiances are available with a mere 1-day lag from https://disc.gsfc.nasa.gov/datasets/OML1BIRR_004/summary and https://disc.gsfc.nasa.gov/datasets/OML1BIRR_003/summary from October 2004 to the present.

##### ISS/SOLAR/SOLSPEC

The SOLAR/SOLSPEC (Thuillier et al 2009) was launched to the ISS in 2008. This instrument was similar to that on the ATLAS-3 (see details in Sect. 2.5.1.2 and resulting reference spectrum in Sect. 2.5.1.4), spanning the spectral range 165 to 3000 nm with a resolution between 0.6 and 9.5 nm. The SOLAR/SOLSPEC measured SSI from launch until 2017, thus covering most of Solar Cycle 24. Meftah et al. (2018) report a mean absolute uncertainty of ~ 1.26% (*k* = 1).

##### OMPS

The NOAA Ozone Mapping and Profiler Suite (OMPS) launched first on the Suomi National Polar-orbiting Partnership (S-NPP) on 28 October 2011. It was followed by OMPS instruments launching on the NOAA-20 on 18 November 2017 and the NOAA-21 on 10 November 2022 with future flights planned on the JPSS-4 in 2027 and the JPSS-3 in 2032. Flynn et al. (2014) describe the suite’s ozone-monitoring performance of the first of these suites. While all used solar-irradiance measurements to track on-orbit instrument performance, similarly to other ozone-measuring instruments described above, unlike those other instruments, the OMPS solar-irradiance measurements were sparse and never became a primary OMPS data product. The OMPS is included here since it has a long measurement duration spanning the UV to NIR, should these irradiance records ever be needed.

The OMPS consists of three instruments. The Nadir Profiler covers 250 to 310 nm with 1.1-nm resolution from a double monochromator, and the Nadir Mapper covers 300 to 380 nm with 1.0-nm resolution from a single-grating spectrometer. The performance of these two nadir-viewing spectrometers, which share a common telescope, is described by Seftor et al. (2014). The Limb Profiler, with on-orbit performance described by Jaross et al., (2014), covers 290 to 1000 nm with 1-nm resolution in the UV and 10 nm in the visible from a prism-based limb-viewing spectrometer. All use CCD detectors. The Limb Profiler has two transmissive diffusers that are duty cycled to track diffuser degradation, while the nadir-viewing instruments use two aluminum diffusers to provide relative on-orbit calibrations from observations of the solar irradiance. These solar-irradiance measurements are only acquired every 2 weeks, so have little benefit to SSI records. This lack of priority is reflected in the released OMPS data products, which make Earth-product data available a mere three hours after acquisition from https://www.earthdata.nasa.gov/data/instruments/omps/near-real-time-data but do not include solar irradiances as a provided product.

##### TROPOMI

**Fig. 39:**
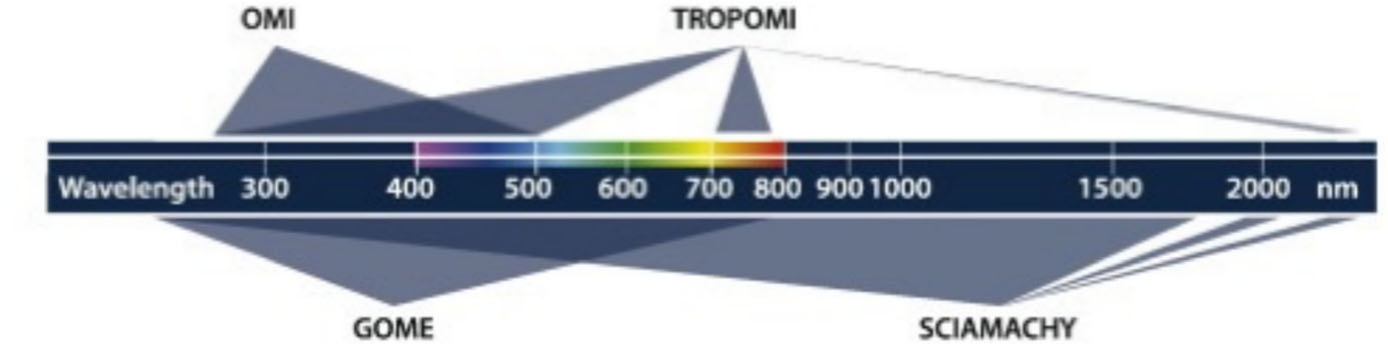
Spectral ranges of TROPOMI, OMI, GOME, and SCIAMACHY. (Cropped version of Figure 2 in Veefkind et al. 2012)

The TROPOspheric Monitoring Instrument (TROPOMI) was launched in Oct. 2017 as the sole instrument on the Copernicus Sentinel-5 Precursor (S5P) satellite and provides daily solar irradiances from 2018 to the present (Veefkind et al. 2012). With heritage from both the OMI and SCIAMACHY, it has three spectrometers that non-contiguously cover 270 to 500 nm, 675 to 775 nm, and 2305 to 2385 nm with spectral resolution of 0.25 to 0.55 nm (and 1 nm below 300 nm). Figure 39 pictorially summarizes the spectral ranges covered by the TROPOMI and related OMI, GOME, and SCIAMACHY. Most similar to the OMI, the TROPOMI consists of push-broom grating spectrometers using 2D CCD detectors. A HgCdTe array is used in the SWIR spectrometer, which includes an innovative immersed grating. Two quartz diffusers allow solar observations for on-orbit radiometric calibrations and duty cycling. These solar measurements also provide spectral calibrations from known spectral features, with the SWIR, being more spectrally uniform, additionally having a set of laser diodes. Similar instruments are intended for launch on the Sentinel-5 MetOp-SG-A and MetOp-SG-B in 2025.

Daily solar-irradiance data, a Level-1B product from the S5P TROPOMI, are available from 28 Aug. 2019 to the present from https://sentinels.copernicus.eu/web/sentinel/data-products/-/asset_publisher/fp37fc19FN8F/content/tropomi-level-1b-irradiances, although they are tedious to download, being daily files. The NASA EarthData site at https://search.earthdata.nasa.gov/search?fi=TROPOMI&fl=1B%2B-%2BRadiance%252C%2BSensor%2BCoordinates hosts daily data from 30 April 2018 to the present. While still being individual daily files, this site offers a “Download All” option.

##### TSIS-1/SIM

**Fig. 40:**
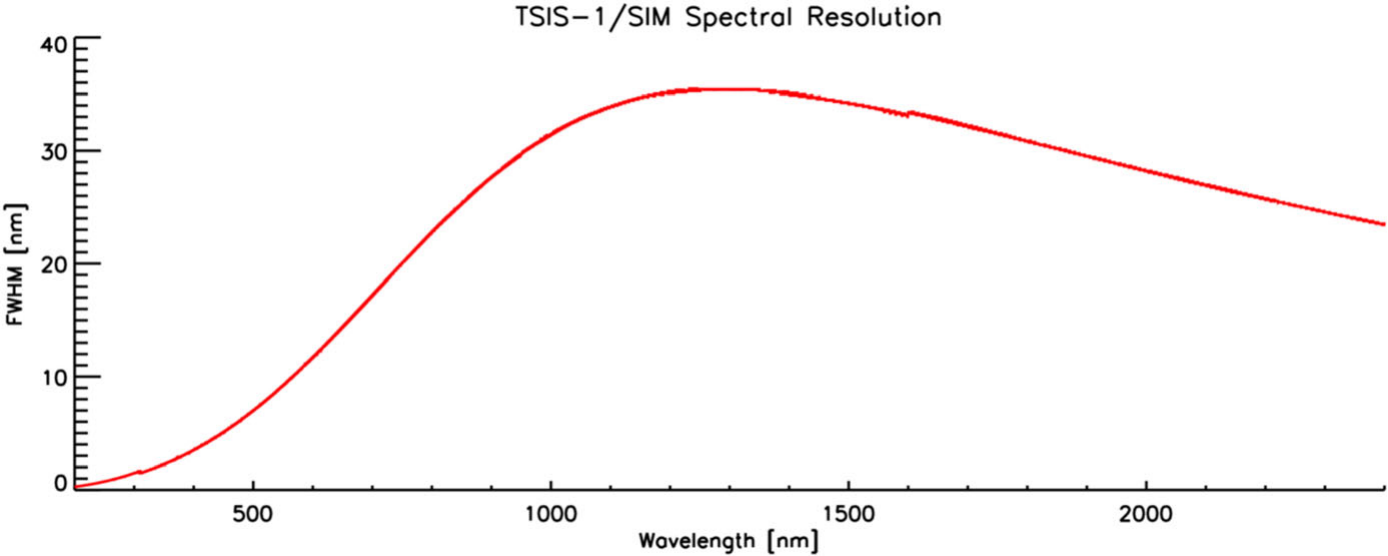
TSIS-1/SIM Spectral Resolution. The TSIS-1/SIM spectral resolution varies between ~ 1 and 34 nm over the 200 to 2400-nm range. The SORCE/SIM is similar

**Fig. 41:**
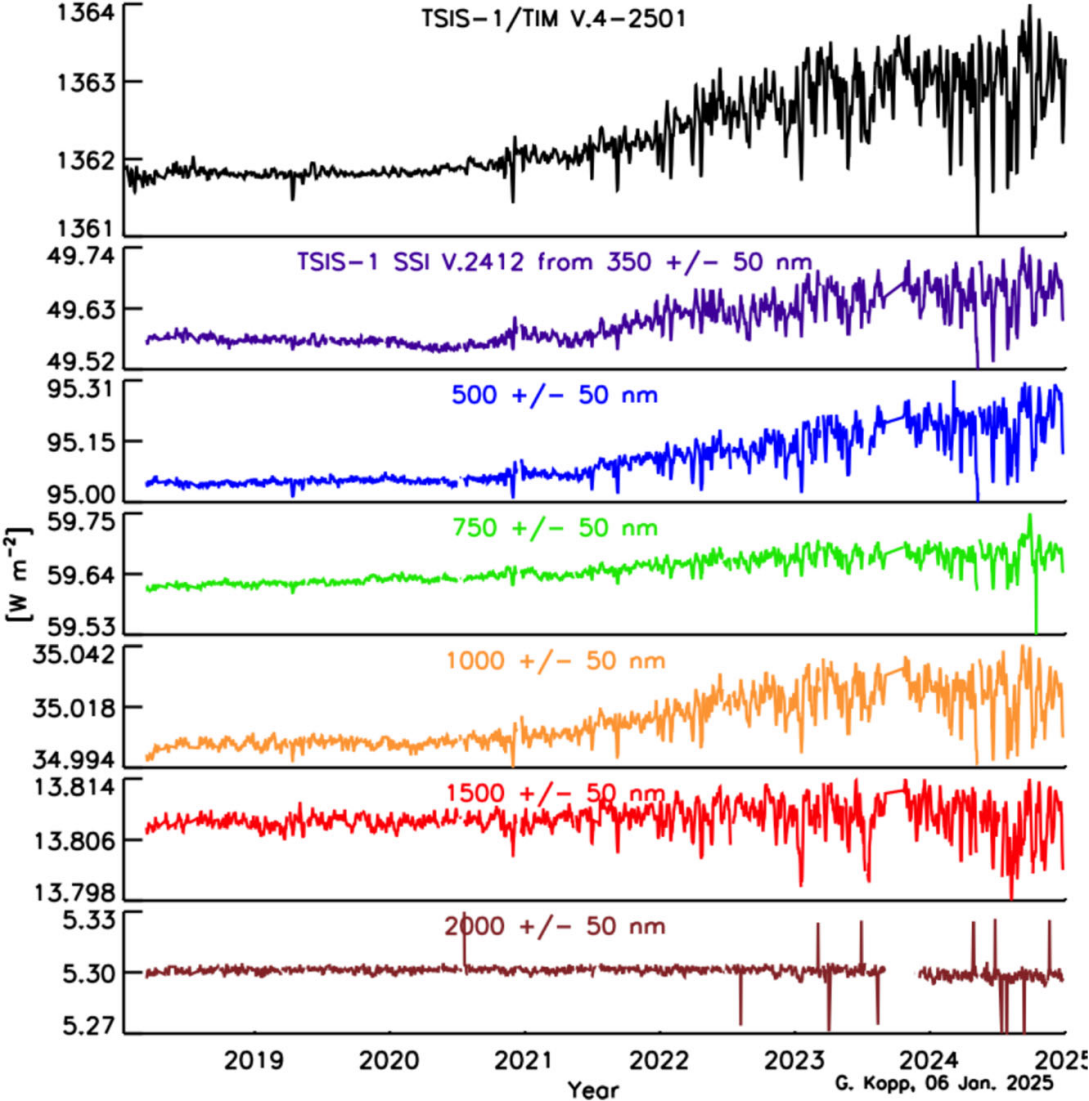
TSIS-1 TSI and binned SSI. The TSIS-1 TSI (top plot) shows increasing activity heading into Solar Cycle 25 that is reasonably similar to the binned TSIS-1 SSI measurements (bottom 6 plots), indicating the greatly improved stability of this instrument’s SIM (compare to Fig. 36)

The TSIS-1/SIM was a significant redesign over that on the SORCE, described in Sect. 2.5.2.8 (Richard et al. 2020, 2024). The most significant improvement was the addition of a third channel rather than the two on the SORCE/SIM, allowing better degradation tracking via duty cycling. The new version covers the wavelength region 200 to 2400 nm with spectral resolution similar to that of the SORCE/SIM and plotted in Fig. 40. Detector performance of the ESRs and photodiodes was improved, with the ESRs now covering the wavelengths longward of 1650 nm. Components thought to have caused outgassing in the SORCE/SIM were better isolated from the optical cavity, reducing inherent degradation. These improvements greatly improved instrument-stability uncertainties and are reflected in the binned SSI time series over the length of the mission shown in Fig. 41 (compare to Fig. 36). All binned spectral ranges except the longest, which is affected by noise, show variations that are in phase with the solar cycle.

Accuracy improvements were also achieved by end-to-end ground-based optical-power and -irradiance calibrations on the newly built Spectral Radiometer Facility (SRF; Richard et al. 2012). The result provides estimated on-orbit accuracies of  < 0.41% at wavelengths below 460 nm and  < 0.24% at longer wavelengths with stabilities of 0.04% year^−1^ below 400 nm and 0.01% year^−1^ at longer wavelengths. The TSIS-1/SIM data show the greatest improvements in accuracy relative to the SORCE/SIM in the NIR, where the TSIS-1 SSI values decrease nearly monotonically relative to the SORCE instrument longward of 700 nm and reach values ~ 6% lower at the longest observed wavelengths.

Although not achieving the climate-driven requirements in Table 2, the TSIS-1/SIM is a significant improvement over all predecessor instruments and provides the most accurate and stable SSI measurements to date. Data are available from 14 March 2018 to the present from https://lasp.colorado.edu/tsis/data/ssi-data/.

##### CSIM

The Compact Spectral Irradiance Monitor (CSIM) is a two-channel plano-prism spectral radiometer operating over 200 to 2800 nm from a 6-U CubeSat flight-demonstration mission launched on 3 December 2018 (Richard et al. 2019). Each channel in this instrument incorporates Si (for 200 to 950 nm), InGaAs (for 900 to 1700 nm), and extended InGaAs (for 1600 to 2800 nm) focal-plane photodiodes to measure the SSI. A miniaturized, full-spectrum carbon-nanotube ESR having 7.2 × lower noise than the TSIS-1/SIM’s ESRs provides calibration accuracy for each channel. The instrument’s spectral resolution increases from about 0.1 nm at 200 nm to 37 nm in the NIR, with 10- to 20-nm resolutions achieved across the visible, which is just slightly greater than that shown in Fig. 40 for the TSIS-1/SIM. As with the SIMs, the refracting-reflecting prism is rotated to scan the solar spectrum across the detectors. The photodiodes can capture a full solar spectrum in as little as 35 min using 0.5-s measurements at each of 2041 stepped-prism wavelength positions. One such scan is done each 12 h. The ESR is much slower, using 500 steps to cover the wavelength range with 20- to 40-s integrations per step. Spread over multiple orbits, a complete reference ESR scan is acquired in 14 days and is repeated on a 30-day cadence. The two CSIM channels are duty cycled in a 14:1 ratio for degradation tracking.

The instrument data are available from https://lasp.colorado.edu/csim/data-and-ham-radio/ from March to November 2019. While covering only a short time period, the CSIM achieved  < 0.5% (*k* = 1) uncertainties via its SRF calibrations and  < 0.05% year^−1^ stabilities, making it one of the most accurate and stable of SSI instruments. As with the CTIM for TSI measurements (Sect. 2.4.10), this technology-demonstration instrument indicates promise of maintaining SSI-measurement continuity via frequent, small, inexpensive, quick-turnaround missions.

#### Narrowband SSI time-series measurements

##### VIRGO SPM

**Fig. 42:**
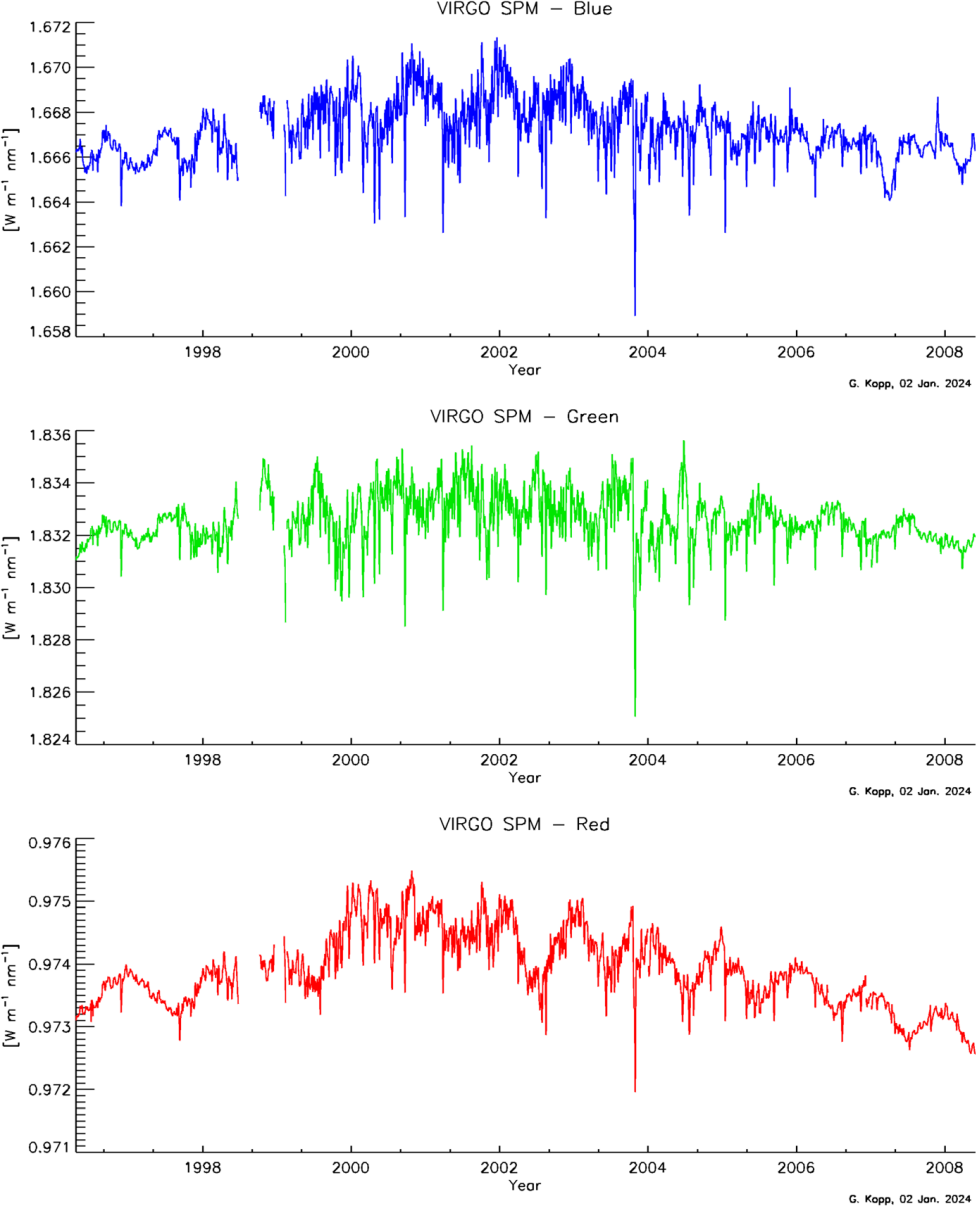
VIRGO SPM data. The degradation-corrected SPM daily data from the blue, green, and red channels are plotted for the early part of the SoHO mission spanning the maximum of Solar Cycle 23. The annual-period oscillations are likely an uncorrected thermal effect, being in-phase with the Sun-SoHO distance. (Data courtesy of C. Fröhlich, ‘SPM_lev20a_d_170496_290508.idl’)

The SoHO/VIRGO’s SunPhotoMeter (SPM) measures the spectral irradiance at 402 (blue in the plot below), 500 (green), and 862 nm (red) with a bandwidth of 5 nm using filters for spectral selection (Fröhlich et al. 1997). While degradation may be a concern over multi-year timescales and a (non-solar) annual-period oscillation is apparent in the data, the duration of this record and the 1-min cadence of the measurements have benefits for high-frequency solar-variability studies spanning solar cycles. Data with degradation corrections applied by the original VIRGO PI are plotted in Fig. 42.

##### PICARD/PREMOS

In addition to the TSI measurements acquired by the PICARD/PREMOS (Sect. 2.4.6.1), the instrument included six spectral-bandpass filter detectors which acquire spectral measurements (Cessateur et al. 2016). The filters are centered at 210, 215, 266, 535, 607, and 782 nm with passbands ranging from 0.58 to 20 nm. Severe instrument degradation and high thermal sensitivity leaving an annual signal require corrections using not only backup detector channels in the instrument but also external proxy data. The resulting time series are useful on solar-rotational timeframes but not on lengthier ones needed for solar-cycle or Earth-climate studies.

##### Spectral-line measurements

High-spectral-resolution integrated-disk measurements show solar variability in spectral lines. Two prominent such lines are Mg II and Ca II, both of which are good indicators of solar activity, particularly faculae. Since the relevant disk-integrated measurement is obtained from core-to-wing ratios, these are relatively insensitive to instrument degradation, although inter-instrument cross-calibrations must be done to create lengthier composite records to account for spectral-resolution differences between instruments. Being in the UV, Mg II h (280.2704 nm) and k (279.5528 nm) are only observable from spacecraft, but Ca K h (396.8469 nm) and k (393.3663 nm) can be measured from ground-based sites. Chatzistergos et al. (2018) developed a method to cross-calibrate ground-based full-disk images of Ca II K going back to 1907 and then used those results to reconstruct the TSI (Chatzistergos et al. 2021). Many historical solar-irradiance reconstructions, such as the prominent ones by Krivova et al. (2003, 2007), Dasi-Espuig et al. (2016), Lean (2018), and others in the comprehensive overview by Chatzistergos et al. (2024) may similarly benefit from these refined Ca K records as facular proxies in the pre-spacecraft era.

## Solar-irradiance composites

**Fig. 43:**
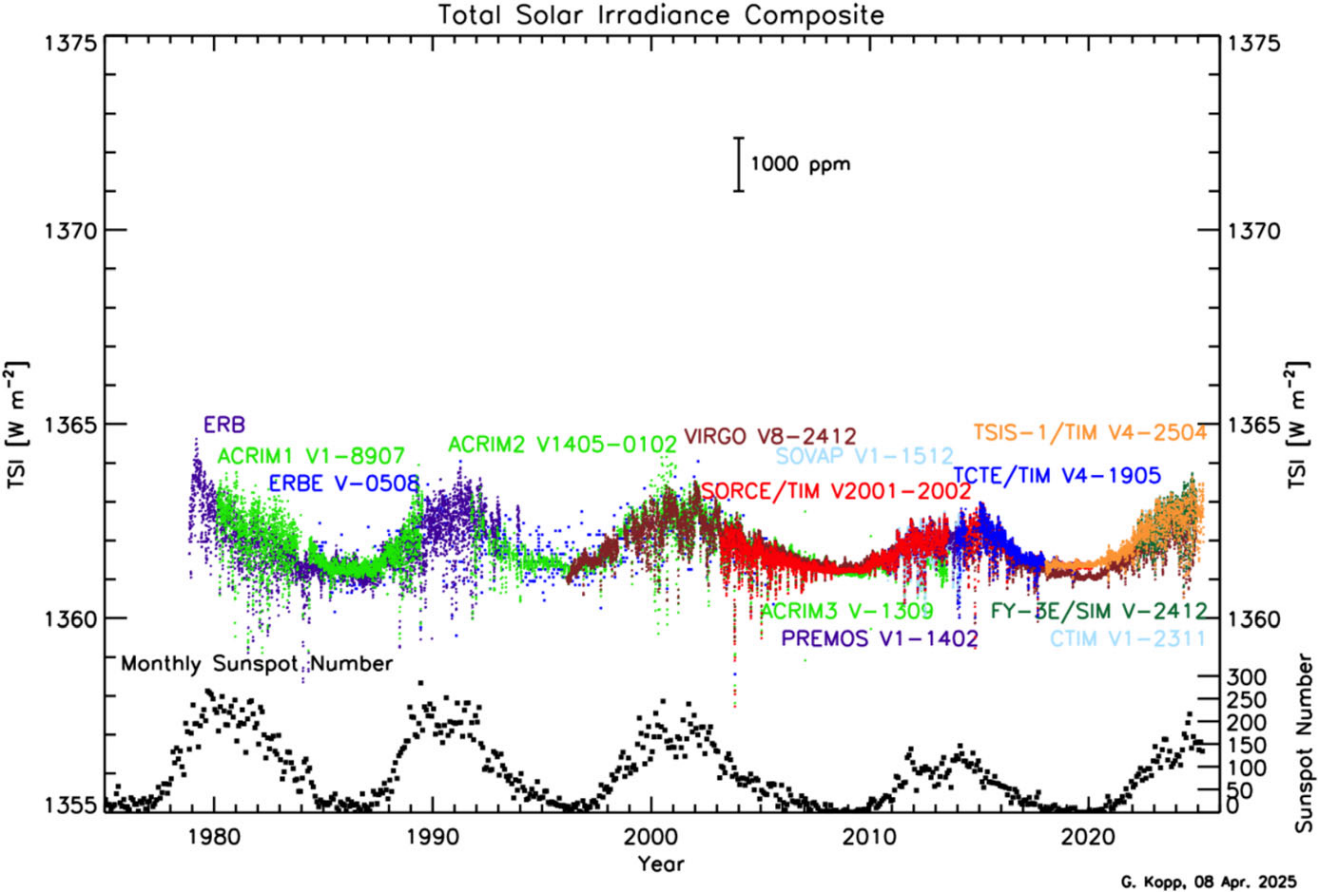
Pictorial TSI composite. Measurement continuity helps correct for instrument-scale differences shown in Fig. 6, allowing each instrument’s data to be scaled to the more-accurate current TSI values for the creation of composite solar-irradiance records

Solar-irradiance composites are the first step in creating long-term irradiance reconstructions needed for Earth-climate studies including those in Intergovernmental Panel on Climate Change (IPCC) Assessment Reports (AR; see IPCC 2021, 2023). Because of the continuity and measurement overlap in space-borne solar-irradiance measurements since 1978, the records from each contributing instrument can be scaled to match the current (and generally more-accurate) instruments’ values. A pictorial composite from the TSI measurements is shown in Fig. 43.

**Fig. 44:**
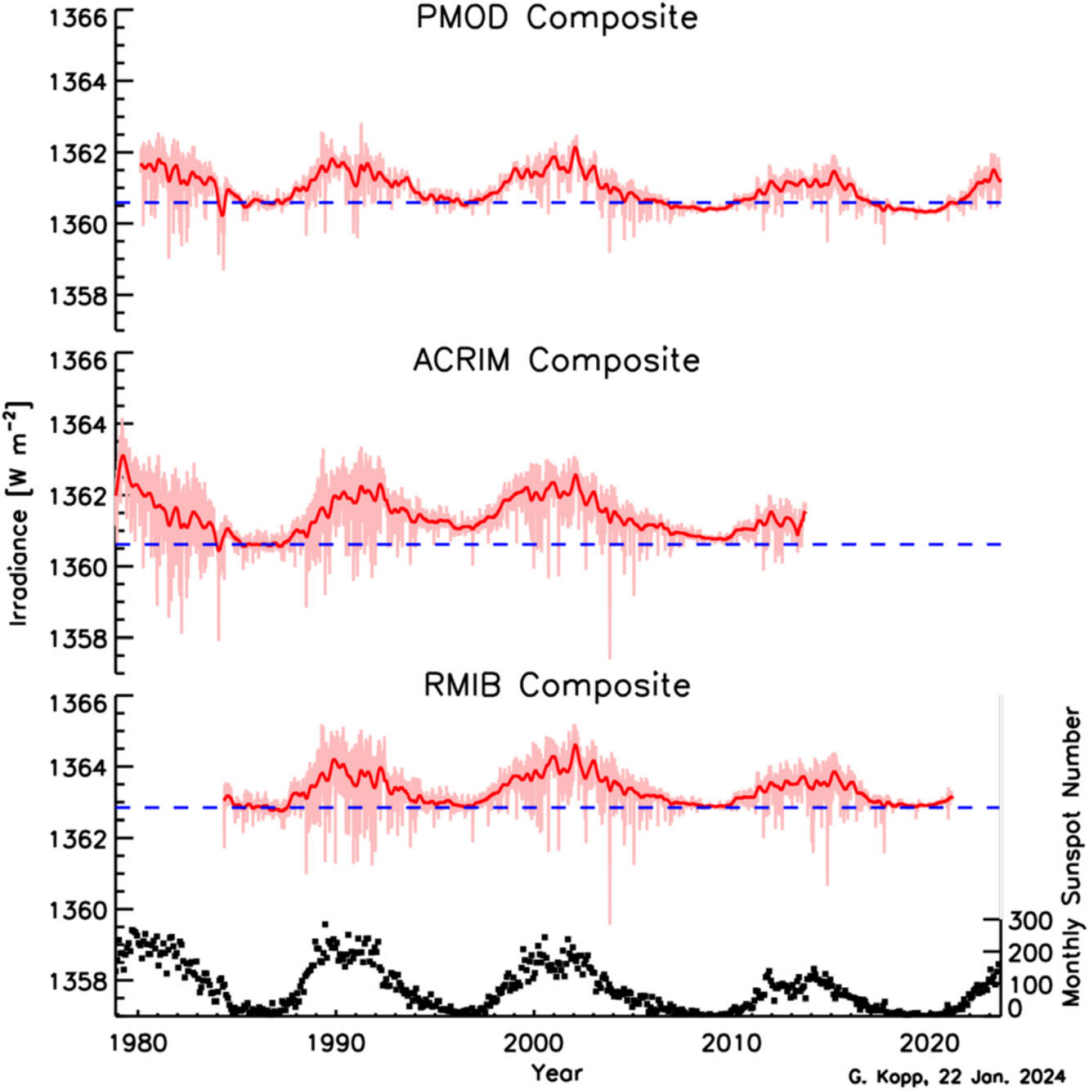
Traditional TSI composites. Three formerly prominent, traditional, PI-created TSI composites show different trends relative to the 1986 solar minimum (indicated by the dashed blue line in each plot) that are due to differing selections of how the contributing instruments are weighted or corrected for use in each composite. These trend differences are indicative of long-term uncertainties in the measurement record, which limit the ability to definitively discern a secular trend over the measurement period. Note also the absolute scales differ for each composite. The monthly sunspot number is shown in the bottom plot (black)

Three formerly prominent TSI composites are produced by different instrument principal investigators (Fröhlich 2006; Willson and Mordvinov 2003; Dewitte et al. 2004) and plotted in Fig. 44. Each is created using a daisy-chaining approach tied to the data from a favored (i.e., that PI’s) TSI instrument during each of several instrument-measurement time-periods. While the three composites generally agree on short-term variations in the Sun’s output, weightings of and corrections applied to individual instruments included in each cause different long-term trends between the three, as not all instruments have equal on-orbit stability. Some researchers adjust instrument data for various effects prior to use in a composite while others do not, causing particularly noticeable differences at the beginning of the measurement era, when the instrument stabilities were the most suspect.

The principal investigators creating these traditional composites applied their in-depth knowledge of the TSI instruments and their artifacts to the composites. Newer composites attempt more unbiased approaches, using data-driven or machine-learning techniques. Each approach has advantages: The former benefits from detailed knowledge of the instruments and the latter from avoiding personal biases. Some of the most prominent resulting composites are described below.

### ACRIM composite

The ACRIM TSI composite is created by ACRIM PI Richard Willson (see Willson and Mordvinov 2003) and uses data from the three ACRIM instruments when available. These instruments measured from 16 February 1980 to 17 September 2013 with a 3-year “ACRIM Gap” between the ACRIM-1 and ACRIM-2 due to the Space Shuttle Challenger disaster delaying the intended, earlier launch of the ACRIM-2 (see Fig. 10). Two other instruments, the NIMBUS-7/ERB and the ERBS/ERBE, provided TSI measurements during that gap. Willson used the Nimbus-7/ERB data (after updates by Hoyt et al. 1992) to link the non-overlapping ACRIM-1 and ACRIM-2 and to provide data for the 2 years prior to the ACRIM–1. Willson uses data provided by the original instrument teams and does not apply any modifications to those data. The contributing instruments and portions are the ACRIMs (73%), the NIMBUS-7/ERB (16%), and the VIRGO (11%). All are normalized to the ACRIM-3 absolute value. The resulting composite is plotted in Fig. 44.

### PMOD composites

#### Original PMOD composite

The original PMOD TSI composite (Fröhlich 2006) was created by VIRGO PI Claus Fröhlich and named after his home institute. He uses the NIMBUS-7/ERB and the ACRIM data prior to 1996, as with the ACRIM composite, but the VIRGO data thereafter. The instruments used for different time periods in the PMOD composite are indicated by differing colors in Fig. 52. Other than the use of the VIRGO data, the PMOD composite differs fundamentally from the ACRIM composite in that it modifies the original instrument-teams’ data for the NIMBUS–7/ERB, the ACRIM-1, and the ACRIM-2. These modifications, which are substantial, are described by Fröhlich and Lean (1998, 2004) and Fröhlich (2006) and are intended to bring consistency between the ERB, ERBE, and models based on ground-based observations. Partial justification for the ERB corrections is that that single-ESR instrument had no means of internally monitoring its on-orbit degradation, but, having similar ESR geometry and optical design to the VIRGO PMO radiometers, could be expected to show similar solar-induced degradation and could thus be adjusted using VIRGO-derived sensitivity corrections. The effect of these adjustments influences the resulting trends across the ACRIM Gap, where most of the long-term differences with the ACRIM composite are instilled. While these corrections have been the source of much controversy, they do have support from newer, independent statistical analyses (see, for example, Amdur and Huybers 2023). The final PMOD composite with these applied corrections is scaled to the absolute value of the Space Absolute Radiometric Reference (SARR), being the average of eight radiometers from April 1992 measurements (Crommelynck et al 1995). This composite shows a gradual linear downward trend up to 2018 compared to other composites and instruments (see Fig. 44, which shows an updated PMOD composite and is scaled to the now-accepted lower TSI value).

#### Current PMOD composite

Fröhlich continued to update the original PMOD composite—and the VIRGO data that directly went into it—until 2018, when an approach inspired by the unbiased, statistical-based methodology of Dudok de Wit et al. (2017) was implemented by Montillet et al. (2022). These authors use a frequency-dependent data-fusion method that considers stochastic noise to discriminate short- and long-term correlations. They combine the TSI data using a maximum likelihood estimator of separate, sparse Gaussian processes after gap-filling daily data via interpolates. The de-weighting of high frequencies by the data-fusion approach is compensated by a subsequent step using a wavelet filter to reconstruct the high frequencies based on the earlier (Fröhlich 2006) composite. The result is scaled to the new, lower TSI value (Kopp and Lean 2011; Prša et al. 2016) and is plotted in Fig. 44. This composite shows a downward trend relative to other composites and instruments up to 2018, as the original version by Fröhlich did, followed by a steady upward trend thereafter. It is curious that this turning point corresponds to the time when the new PMOD composite-creation methodology was implemented.

### RMIB composite

The Royal Meteorological Institute of Belgium (RMIB; now the Royal Observatory of Belgium, ROB) composite (Dewitte et al. 2004) uses a simple daisy-chaining approach after scaling the included instruments’ data to a common value. The instruments used include the ERB, ACRIMs, SOLCON, SOVAs, VIRGO, and SORCE/TIM. As with the original PMOD composite, this composite is scaled to the SARR and thus has the older—and much higher than currently accepted—absolute value.

An update to this composite by Dewitte and Nevens (2016) reviews overlapping data and rejects data from time periods when the authors believe certain instruments are suspect. The remaining data are averaged for each day and daisy chained. This composite, shown in Fig. 44, is scaled to the SOVIM/DIARAD instrument’s averages of both channels, giving an absolute value of 1362.9 W m^−2^ at solar minimum.

### Community consensus TSI composite

The PI-created ACRIM, original PMOD, and RMIB composites have three primary issues:

1. They reflect the composite-creators’ biases. All are singular in time, selecting data from only one instrument for any given day. That selection tends to favor the data from the composite-creator’s own instrument(s) at times when those data are available. Figure 52 shows an example of this single-instrument-selection method for the PMOD composite. This methodology highly weights the instrument chosen by that researcher and largely ignores concurrent instruments, possibly not benefitting from those other data which could perhaps indicate erroneous values from the single instrument chosen for the composite at a given time.
2. Corrections to data are not uniformly applied. Some researchers adjust data from other instruments to get agreement. The PMOD composite includes the most such corrections, particularly to data from the earlier space-borne TSI instruments. These corrections are intended to make those instruments’ data more consistent with other indicators of solar variability or with other instruments (Fröhlich 2006), imposing self-consistency via the applied corrections. Willson and Mordvinov (2003) instead argue that the original instrument teams knew best the most appropriate corrections to their data, and they do not apply any corrections to the instrument data used in the ACRIM composite.
3. None of the composites shown in Fig. 44 include uncertainties to indicate how well the stated values are known as a function of time. Those uncertainties might be expected to vary with instrument aging, operational changes, or thermal variations.

**Fig. 45:**
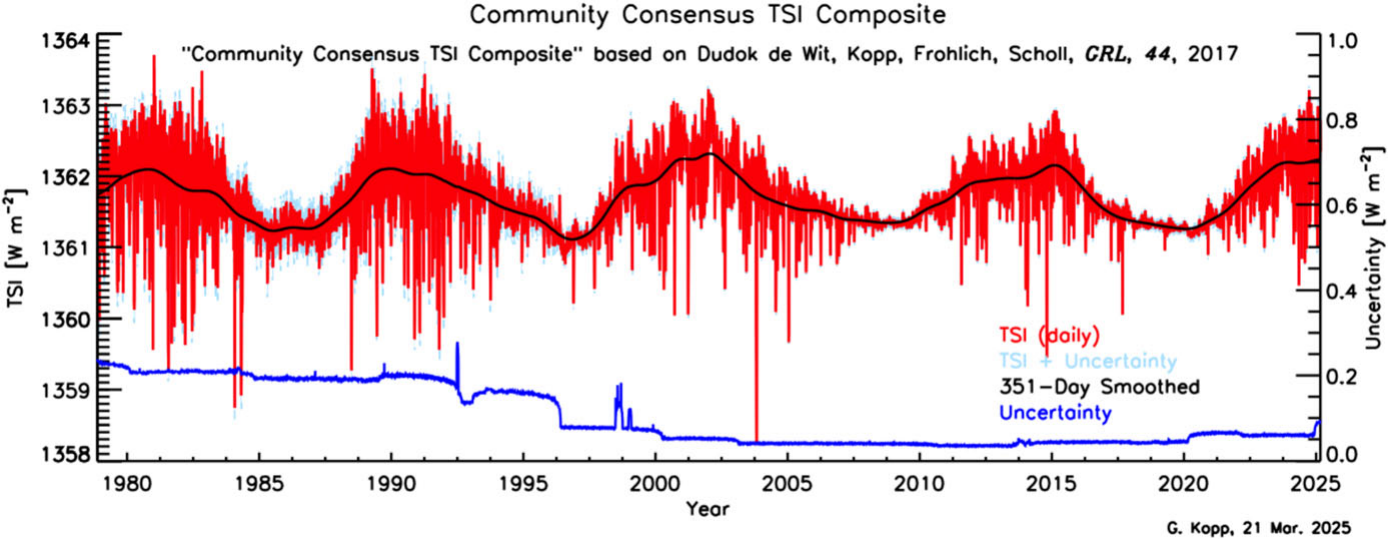
Community consensus TSI composite. The Community Consensus TSI Composite uses an unbiased statistics-based methodology to estimate instrument uncertainties and weightings to produce a TSI composite over the space-borne TSI-measurement era. The daily TSI values are shown in red with a near-annual smoothing in black using the left-hand vertical axis. The blue points indicate the statistical uncertainties in the composite using the right-hand vertical axis (note the large differences in axis scales). The light-blue values show the TSI measurements with those uncertainties added, although they are nearly indistinguishable from the data themselves due to the scale of the uncertainties. Note that the uncertainties are lower for the more recent (and more accurate) instruments and at times having a greater number of available instruments. (from http://spot.colorado.edu/~koppg/TSI)

These issues have now largely been addressed by the TSI community via an International Space Science Institute (ISSI) team that included the PIs representing most TSI instruments at the time (2012–2014). The various instrument teams provided insights to assess the uncertainties and stabilities of each TSI data record. End-users, such as solar- and climate-modelers, provided guidance for the final product, such as desires to not have to delve into the subtle details of each instrument or the corrections included as well as the importance of having continual-cadence daily data with uncertainties. In estimating measurement accuracies, this team reviewed instrument designs and calibrations, assessed on-orbit performance, and studied correlations between the various datasets contributing to the TSI data record. The ISSI team agreed on a composite-creation methodology that avoided individual-team biases, selecting a statistical methodology formulated by Dudok de Wit et al. (2017) that incorporates a maximum-likelihood approach with common-mode analyses between data from multiple instruments. A data-driven approach removes personal biases in estimating instrument weightings. The resulting “Community Consensus TSI Composite” applies a time-dependent and scale-wise (i.e., frequency dependent) weighting to the values for all available instrument data, weighting the most-accurate data at any time while benefitting from the use of the collective data. Time-dependent uncertainties are provided with the TSI values so that end users can discern times of better agreement between contributing instruments. This was the first attempt to create a TSI composite with a more comprehensive (by including all instruments) and less biased approach than the traditional PI-created composites. An updated version of this methodology gives the resulting composite in Fig. 45 and is available from http://spot.colorado.edu/~koppg/TSI.

### An ensemble of TSI composites

Starting with some of the TSI composites above, Connolly et al. (2024) created an additional 17 composites following each of the different approaches used by the original composites, updating many to include data to the near present. They don’t recommend a single best composite or even grouping of composites, leaving that to the reader. Rather than solve (and justify) that selection, the reader may best consider this collection of 21 composites as an ensemble indicating the variability of different composite-creation methodologies.

### SOLID SSI composite

**Fig. 46:**
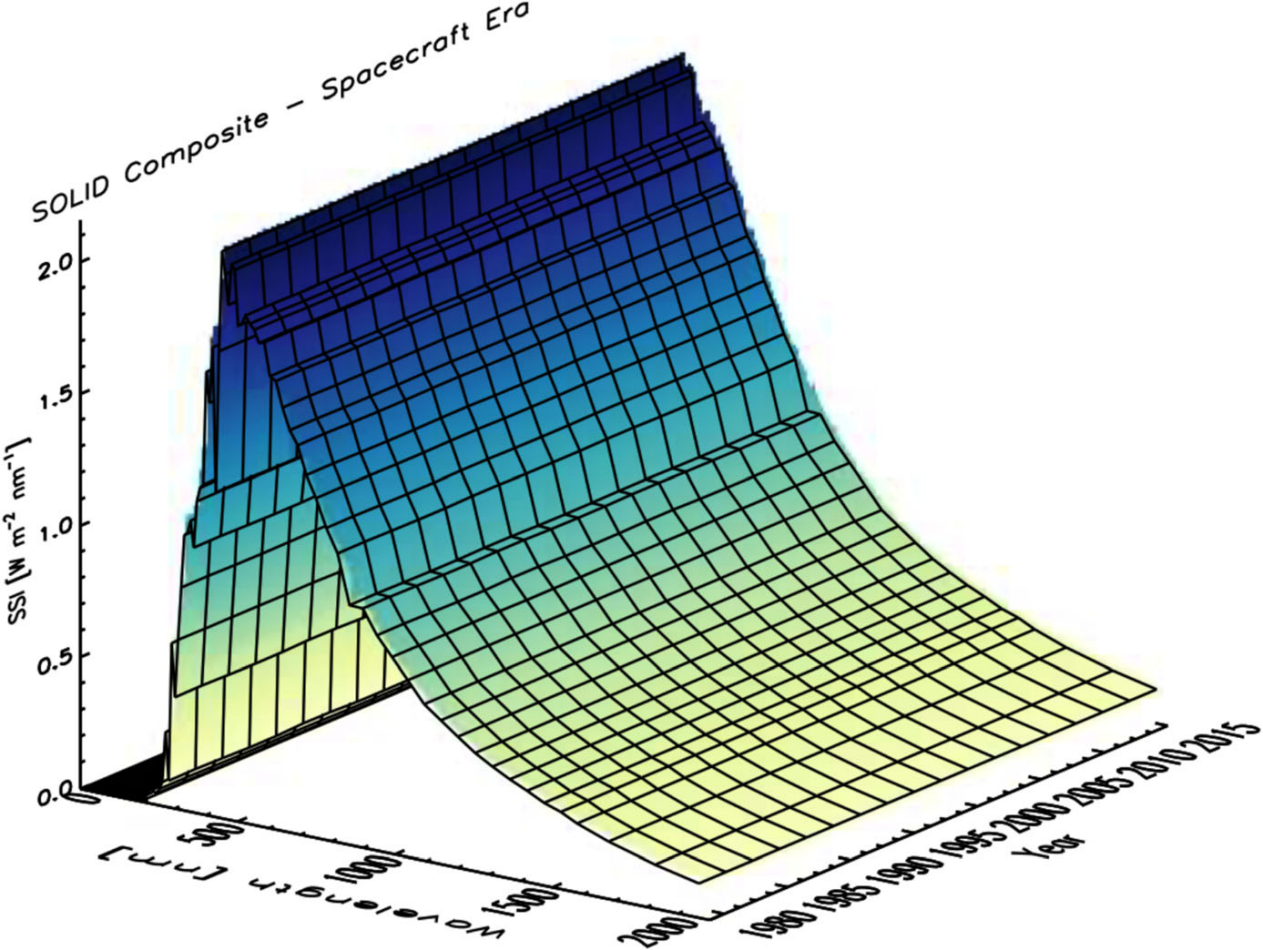
SOLID composite. This surface plot shows the SOLID SSI data over the spacecraft era. The full data extend from 1951 through 2014. Variations in time are nearly imperceptible on this scale

The ‘‘First European Comprehensive Solar Irradiance Data Exploitation project’’ (SOLID; http://projects.pmodwrc.ch/solid with data available at ftp://www.pmodwrc.ch/pub/) composite (Schöll et al. 2016; Haberreiter et al. 2017) is a spectral-irradiance composite combining time-series spectra from 20 different instruments, six solar proxies, and nine reference spectra. There is no attempt to correct any of the input data, intending this as a purely observational composite. An unbiased probabilistic approach applies weightings based on scale-wise uncertainties from each of the inputs, similar to the method applied by Dudok de Wit et al. (2017) for the Community Consensus TSI Composite. The SOLID composite temporally re-grids, interpolates, and gap fills to provide daily values and their uncertainties over the spacecraft era from 1 November 1951 to 31 December 2014 with wavelengths from 0.5 to 1991 nm with 1-nm resolution. The datasets used in this composite are graphically shown in Figure 1 of Schöll et al. (2016) and reproduced here as Fig. 24. This figure helps convey the level of complexity in making a composite dependent on time and wavelength with limits to data availability having temporal and spectral continuity. A surface plot of the resulting SOLID composite over the spacecraft era (8 November 1978 to 31 December 2014) is shown in Fig. 46.

### GSFCSSI2 and LASP-GSFC-SSI3 composites

The GSFC Composite Solar Spectral Irradiance (GSFCSSI2; DeLand et al. 2019) combines data from nine satellite instruments, including the SME, Nimbus-7 SBUV, NOAA-9 SBUV/2, NOAA-11 SBUV/2, UARS SUSIM, UARS SOLSTICE, NOAA-16 SBUV/2, Aura OMI, and SORCE/SOLSTICE. The daily-averaged data span the wavelength range 120.5 to 500 nm in 1-nm bins. While this includes variability of the important Lyman-*a* line, the authors recommend using the Lyman-*a*-specific composite time series provided by Machol et al (2019) instead. Updates by DeLand and Marchenko in 2023 extend the data from 8 Nov. 1978 to 24 July 2022 and normalize the spectrum to the HSRS (Sect. 2.5.1.7).

**Fig. 47:**
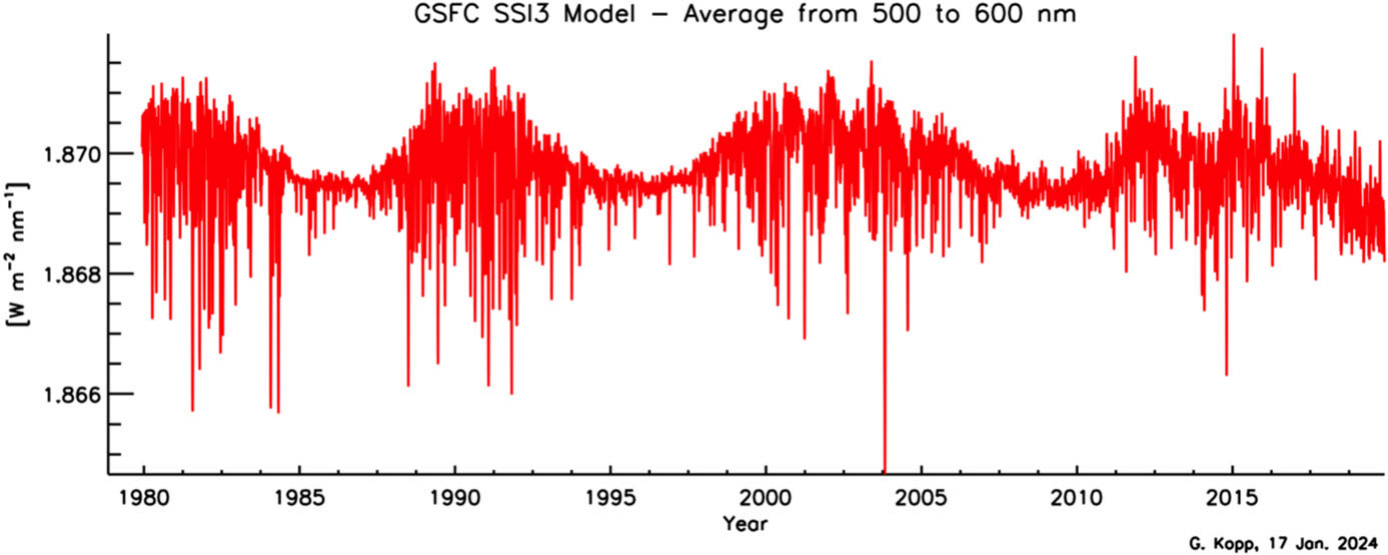
GSFC SSI3 composite. This SSI composite spans a broad wavelength range by combining instrument measurements and solar proxies. The average over a 100-nm mid-visible bandpass is shown

The LASP GSFC Composite Solar Spectral Irradiance (LASP-GSFCSSI3; Woods and DeLand 2021) extends the GSFCSSI2 wavelength range down to 0.5 nm using SORCE/XPS (0 to 6 nm), SDO/EVE (6 to 33 nm), and TIMED/SEE (33 to 120 nm) and up to 1597.5 nm using SORCE/SIM data. The composite covers the time range 8 Nov. 1978 to 21 Nov. 2019 (see Fig. 47). The UV part of the GSFCSSI2 composite is scaled to the SORCE/SOLSTICE, which has better radiometric accuracy than previous observations, and the GSFCSSI2 is adjusted to agree with the TSIS-1/SIM irradiance over the 500 to 1600-nm spectral range on 14 March 2018. These longer-wavelength corrections are less than 1% from 500 to 900 nm but up to 5% from 900 to 1600 nm. Several solar-activity proxies, including sunspot number, Mg II, Lyman-*a*, F10.7, and the TSI, are used to adjust the time series at each wavelength. 81-day averages of the proxy models remove suspected instrument trends, while data gaps are filled with short-term variations in the proxies.

The GSFCSSI2 composite is available from https://lasp.colorado.edu/lisird/data/gsfc_composite_ssi and the LASP-GSFC-SSI3 composite from https://lasp.colorado.edu/lisird/data/lasp_gsfc_composite_ssi.

## Solar-irradiance variability

Estimates of solar-irradiance variability on all timescales except Milankovitch and stellar evolutionary are linked to the TSI measurement record. Augmenting space-borne measurements with ground- or space-based imaging observations of the Sun enables understandings of the causes of irradiance variability. Correlations of irradiance measurements with sunspots and faculae occur on timescales ranging from several hours to the solar cycle (see earlier discussions in this article or recent summaries by Kopp 2016 and Shapiro et al. 2017). Correlating measured irradiances with such surface manifestations of time-varying magnetic activity, as described in detail by Domingo et al. (2009), enables longer-term knowledge of those features to estimate solar irradiances prior to the direct-measurement record. Proxies of faculae extend back over a hundred years while the observational sunspot record spans more than 400 years. Modulation of cosmogenic isotopes by solar variability further extends historical irradiance reconstructions over millennia (i.e., Lean 2018; Wu et al. 2018; Usoskin 2023).

The time variability of solar irradiances on several timescales is described below.

### Short-term variability

**Fig. 48:**
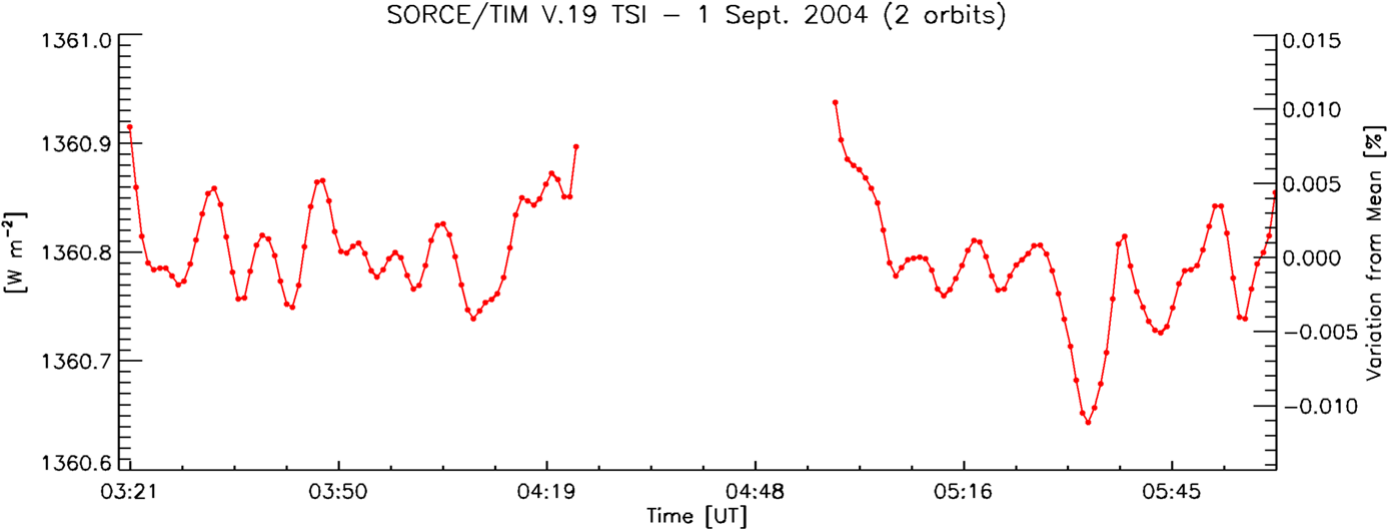
Short-term TSI variability. The TSI varies continually at the ~ 0.01% level on timescales of minutes due to the superposition of convection and oscillations on the visible solar disk (updated from Kopp et al. 2005a, b)

On timescales of minutes to hours, the TSI varies at the ~ 0.01% level due to the globally averaged superposition of solar convection and oscillations (Fröhlich and Lean 2004; Kopp et al. 2005a, b; Kopp 2016). These ever-present variations in TSI have periods in the 3- to 10-min range. An example time-series of such short-term variability is shown in Fig. 48.

**Fig. 49:**
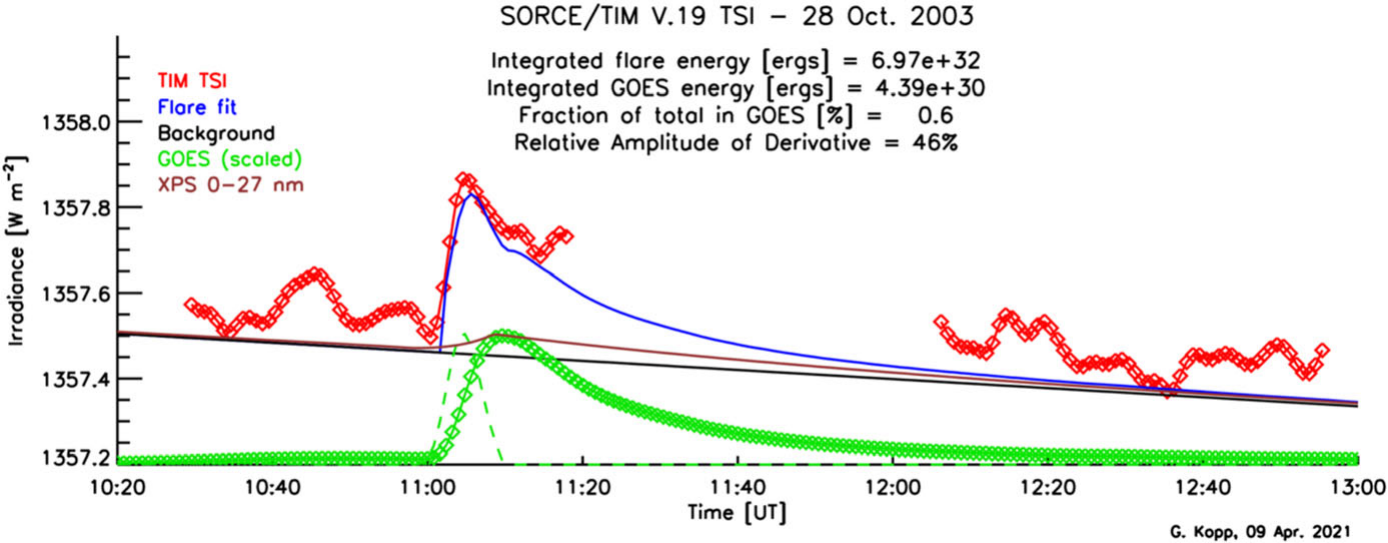
Flare measurement in TSI. The X17 flare on 28 October 2003 caused an abrupt, short-duration 0.028% increase in the TSI. Data shown are from the SORCE/TIM with 50-s cadence (red) and the GOES XRS (green). The TSI measurements enable estimates of the total radiant flare energy

Large solar flares have occasionally been observed in TSI (Woods et al. 2003; Woods et al. 2006). While prominent in the ultraviolet and X-ray spectral regions, where the background intensity from the quiet Sun is low, flares are relatively minute in both spatial extent and in net energy compared to the TSI. The fourth largest flare ever recorded by the GOES X-Ray Spectrometer measurements, an X17 flare near disk center on 28 October 2003, caused an abrupt but short-duration 0.028% increase in the TSI (Kopp et al. 2005a, b; Kopp 2016, 2021). As shown in Fig. 49, the initial impulsive phase of this flare unambiguously exceeded the ever-present background variations from disk-averaged solar convection and oscillations. Being measurements of the spectrally integrated energy of the Sun, TSI observations of flares provide the total radiant energy released by the flare, which is not available from spectrally limited observations. The time average of the TSI signature of this flare in Fig. 49 gives an energy more than 100 × that in the GOES X-ray bandpass.

Extended-duration full-spectral-range SSI measurements do not exist at these high cadences. Scanning spectral-irradiance instruments must sweep through wavelengths to acquire an entire spectrum. This operational methodology is incompatible with obtaining high-cadence spectra. The grating-based spectrometers, which acquire the entire spectrum quickly and can make high-cadence measurements, are exclusively Earth-atmosphere instruments and acquire solar-irradiance measurements intermittently—usually on a daily cadence—so generally only obtain measurements lasting a few minutes.

Since they produce so little energy compared to the total radiant power from the Sun and since they occur on such short timescales that the Earth’s climate cannot respond to them, solar variability caused by flares, convection, and oscillations has no direct effect on the Earth’s climate.

### Variability on solar-rotation timescales

On 27-day (solar rotational) timescales, Willson and Hudson (1991), using the early ACRIM-1 data, report that sunspots account for the majority of the TSI variability on timescales of a few days while the more spatially extended and longer-lasting faculae (the magnetically active regions that generally surround and are associated with sunspots) account for most of the variability over a few weeks.

The passage of sunspots across the Earth-facing portion of the solar disk causes short-term decreases in the TSI and is responsible for most of the high-frequency variations in the daily data shown in Fig. 6. These generally cause few-day dimmings of ~ 0.1%, although the passage of large sunspot groups across the disk in late October 2003 dimmed the Sun by 0.34% and was the greatest short-term decrease in TSI ever recorded (Kopp et al. 2005a, b; Kopp 2021). Dimmings due to sunspots are most pronounced by spots near disk-center, as foreshortening and the Wilson depression reduce sunspot contrast nearer to the solar limb. These effects hold for SSI at wavelengths in the visible and infrared; sunspots are not pronounced at wavelengths shorter than ~ 350 nm.

**Fig. 50:**
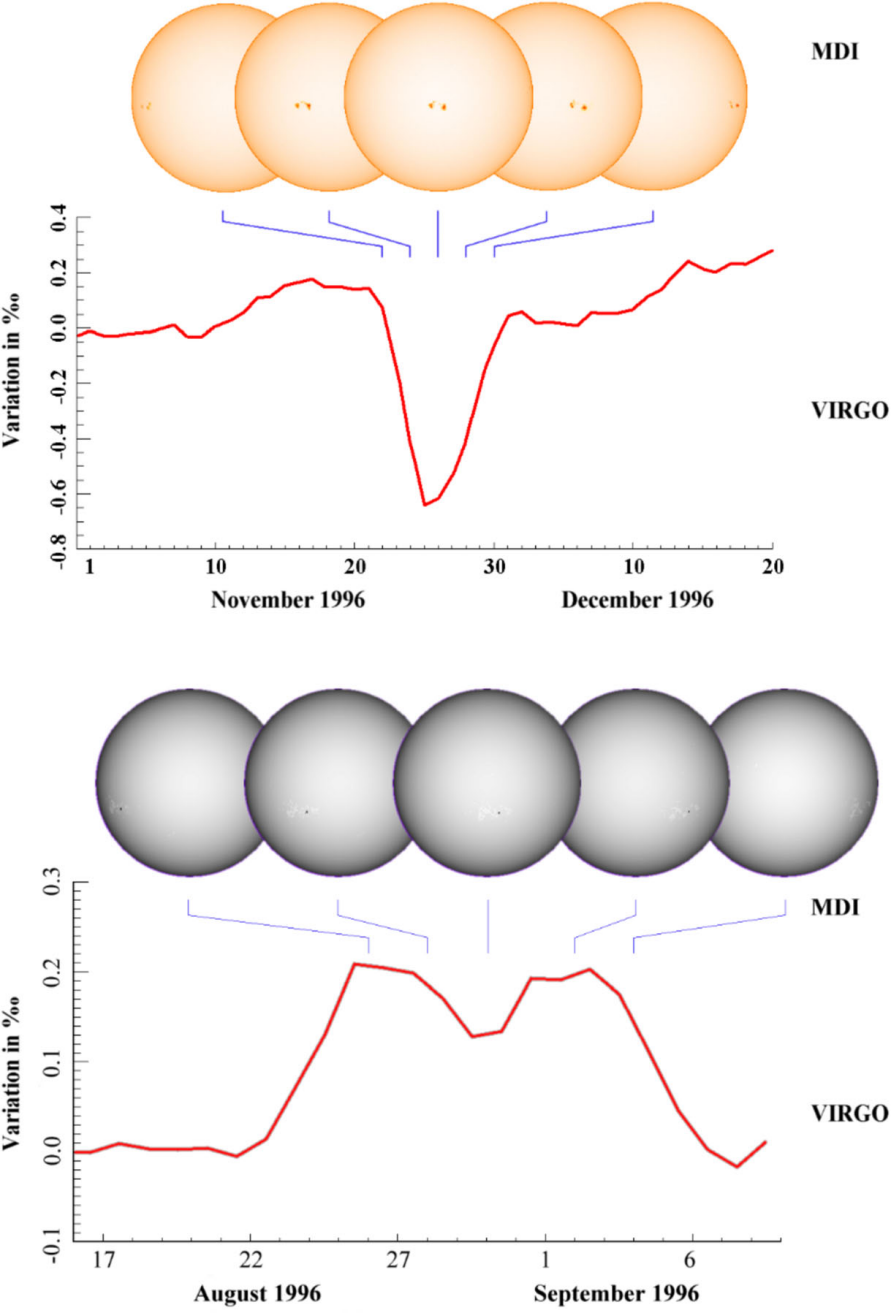
TSI response due to sunspot and faculae center-to-limb variations. This figure shows the TSI variability due to solar-rotation timescale passages of sunspots (top) and faculae (bottom) with their different center-to-limb variations. Image reproduced with permission from Solanki and Fligge (2002), copyright by ESA

Faculae, on the other hand, continue to be bright at UV wavelengths and remain prominent near the limb, having different center-to-limb behavior than sunspots (see Fig. 50). This, combined with their longer lifetimes and greater areal extents, results in facular brightening generally dominating the opposing sunspot-dimming effects in time-averaged solar-irradiance variability on solar-rotational and longer timescales.

Fröhlich and Lean (2004) and Lean (2010) determined that much of the observed short-term variability can be estimated by empirical proxy-models based solely on linear regressions to sunspot and facular components. A more sophisticated semi-empirical model (Solanki et al. 2005; Krivova et al. 2011; Ball et al. 2011; Yeo et al. 2014), which includes additional solar-activity indicators combined with physical models to provide further refinements, obtains a similar fundamental conclusion—the irradiance variability on solar-rotation timescales is largely due to the opposing effects of sunspot darkening and facular brightening. Shapiro et al. (2017) analyze brightness power spectra, pointing out that at certain periods, these effects may balance, even during times of high solar activity, having the unexpected side-effect of making the solar-rotation period difficult to discern in disk-integrated broadband photometric measurements.

On solar-rotation timescales, both grating- and scanning-based spectral instruments acquire useful SSI measurements. These are timescales over which instrument degradation does not vary significantly, so both the Earth-looking and solar-dedicated spectrometers have reasonable stability. One advantage of the grating-based (Earth-looking) instruments is that they acquire the full solar spectrum at a consistent daily time (usually at terminator crossings) rather than measuring different wavelengths at different times throughout each day, as is the case for solar-dedicated scanning spectrometers.

**Fig. 51:**
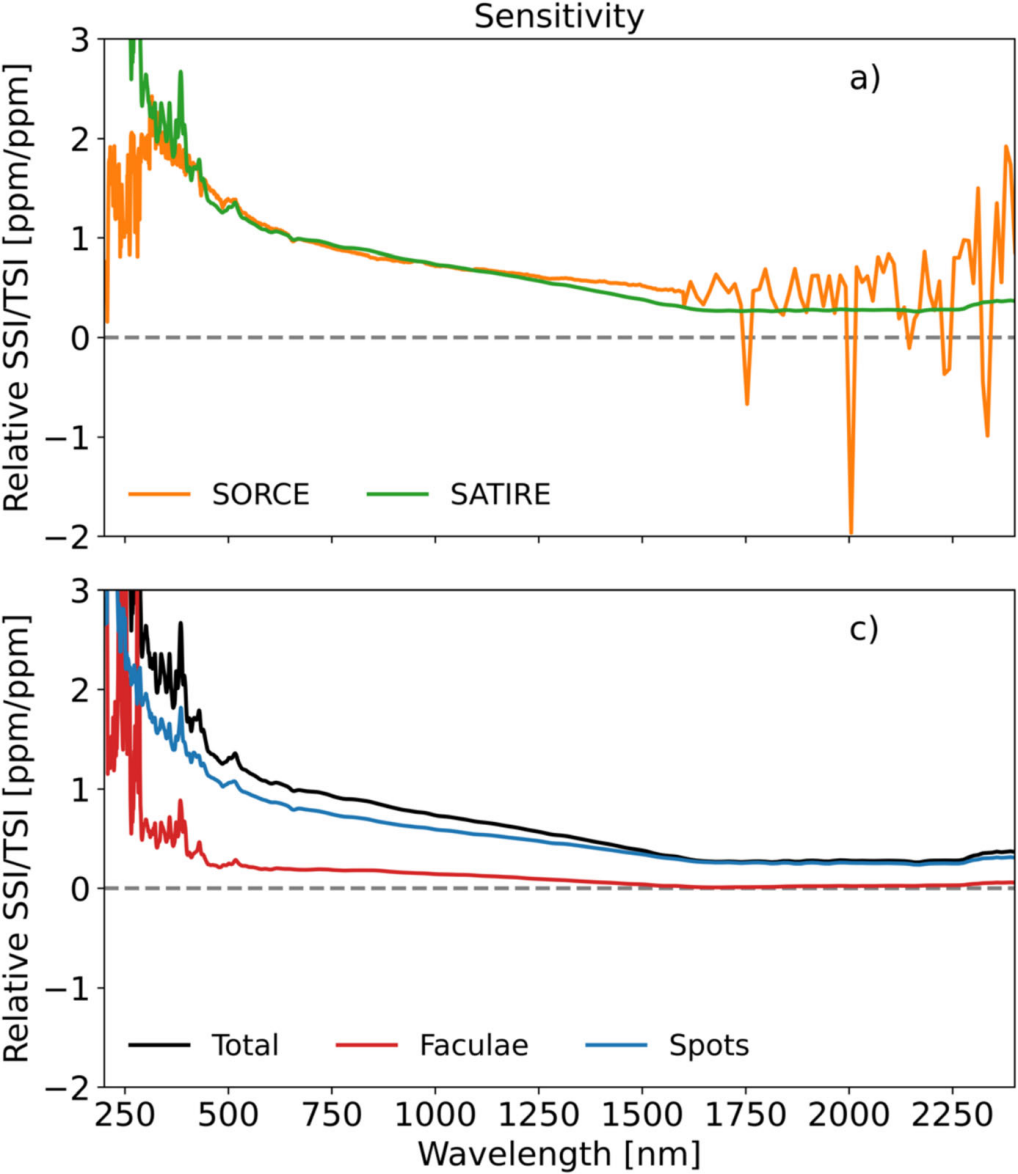
SSI sensitivity to TSI variability. On solar-rotation timescales, the SSI varies by nearly twice the TSI at wavelengths near 400 nm, by the same relative amount at around 650 nm, and by half of the relative TSI variability in the NIR (top plot). This correlation holds for both empirical data (orange; from SORCE/ TIM) and models (green; SATIRE-like model). The individual contributions from sunspots and faculae are shown in the bottom plot. Image reproduced with permission from Kopp et al. (2024), copyright by the author(s)

From daily spectral measurements, Kopp et al. (2024) show that the SSI is highly correlated to the TSI on timescales up to at least seven solar rotations, such that TSI variability provides a good estimate of the SSI variability at wavelengths longer than 400 nm according to the approximation to a small bolometric response given by

$$ \frac{\Delta SSI}{{SSI}} \approx \frac{5}{8\lambda }\, \cdot \,\frac{\Delta TSI}{{TSI}}\quad \left( {{\text{with}}\;{\text{wavelength}}\;\lambda\;{\text{in}}\;{\text{microns}}} \right) $$

(see also Fig. 51). At shorter wavelengths, the correlation breaks down because sunspots do not cause significant irradiance changes in the UV. This relation enables an estimate of the SSI at times when spectral measurements are not available.

### Variability on solar-cycle timescales

**Fig. 52:**
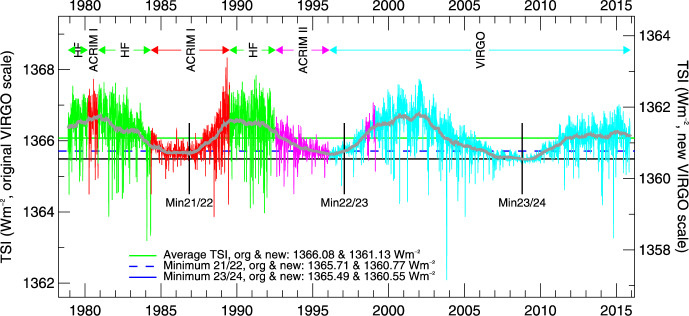
“Original” PMOD TSI composite. The PMOD TSI composite by Fröhlich shows peak-to-peak TSI variability of slightly less than 0.1% in each of the three solar cycles observed during the space-borne measurement record, with that variability being in phase with solar activity. Different colors indicate the binary selections of each instrument used in the creation of the composite. The right-hand vertical scale indicates the more-accurate, currently accepted absolute value. [Courtesy of C. Fröhlich and the VIRGO team via ftp://ftp.pmodwrc.ch/pub/Claus/ISSI_WS2005/ISSI2005a_CF.pdf]

On solar-cycle timescales, the TSI varies by ~ 0.1% in phase with the solar cycle, as reported early in the measurement record by Willson and Hudson (1991) using the ACRIM-1 data (see Sect. 2.4.2). The TSI variability over subsequent solar cycles has shown similar magnitudes. The (original) PMOD composite, shown in Fig. 52, indicates peak-to-peak amplitude variations of 0.082%, 0.077%, 0.096%, and 0.063% respectively for the peaks of each of Solar Cycles 21 (peaking in 1980) through 24 using annual medians of daily measurements.

SSI variability on solar-cycle timescales is less well quantified, as the instruments acquiring these measurements do not currently have the time duration and stability needed to definitively detect variations on multi-year timescales across the spectrum, as discussed in Sect. 2.5.2, although the TSIS-1/SIM shows promise of such detections with extended temporal observations. Kopp et al. (2024) showed that the SSI-to-TSI-variability sensitivities plotted in Fig. 51 applied to each individual year across a solar cycle, so it may be reasonable to extrapolate that relationship, which is based on timescales of seven solar-rotations but consistent annually across a solar cycle, to solar-cycle timescales, providing some estimate of the solar-cycle variability of the SSI at wavelengths longer than 400 nm using the better-known TSI solar-cycle variability. As the relationship between sunspots and faculae is not straightforward, however, improvements in SSI measurements will hopefully soon better determine the solar-cycle variability across the broad spectrum. The UV variability, being larger than that at visible and NIR wavelengths, is better known. The overview given by Rottman (2005) and summarized herein in the introduction to Sect. 2.5 gives these variabilities for different timescales, including those of the solar cycle.

As with solar-rotation timescales, the majority of the TSI fluctuations on solar-cycle timescales are the result of opposing brightenings from faculae and shorter-duration dimmings from sunspots. Lean (2010) finds that over a solar cycle or longer these two manifestations of solar-surface magnetic activity explain 93% of the TSI variability observed by the SORCE/TIM instrument and 83% of that in the PMOD TSI composite. Coddington et al. (2016) explain 92% of the SORCE/TIM TSI variability with these two components. Chapman et al. (2012) use different solar proxies than Lean to represent sunspots and faculae but obtain very similar results on these timescales. They report that two photometric sums obtained from ground-based observations at the San Fernando Observatory (SFO) explain 95% of the SORCE/TIM variability over the first 7 years of its measurement record. Applying their empirical method over the same 7-year time-range to three TSI composites, these authors explain 88.7% of the variability in the ACRIM composite, 92.2% of that in the PMOD composite, and 92.4% of that in the RMIB composite (Chapman et al. 2013).

The variations in irradiance over the 11-year solar-cycle is of sufficient magnitude and duration to influence Earth’s climate on these timescales. The typical ~ 0.08% solar forcings on 11-year timescales can be seen in global- and regional-temperatures, sea-surface levels, ozone abundance, tree rings, and precipitation amounts (Lean and Rind 2008; Lean 2010; Gray et al. 2010; Haigh 2011). Lean (2010) and Matthes et al. (2017) report globally averaged surface-temperature variations of about 0.1 °C in phase with TSI during recent solar cycles. Lean and Rind (2008) and Haigh (2011) show regional surface-temperature changes can be even larger on these solar-cycle timescales. Solomon et al. (2019) and Chiodo et al. (2012) show altitude-dependent atmospheric temperature and composition variations with the solar cycle (with increases reaching ~ 500 K in the thermosphere at solar maximum) that are consistent with empirical evidence. These correlations over solar-cycle timescales provide definitive Sun-climate connections since both the solar variability and many Earth-climate records were directly measured using modern instruments over the solar-irradiance space-borne measurement-era, so there is no reliance on proxies as there is for comparisons relying on historical reconstructions.

### Secular variability over the space-borne measurement era

While climate signatures due to solar variability on solar-cycle timescales are well established from the TSI record, longer-term climate influences from the Sun are less well-quantified by the space-borne measurement record. Potential long-term irradiance changes, however, would be even more influential than solar-cycle changes, as secular variations in the Sun’s energy could give the Earth’s climate system sufficient time to fully respond.

As with the limited ability of the SSI record to discern solar-cycle changes, the space-borne TSI record has marginal ability to detect a potential solar trend over the multi-decadal duration of the current 47-year record. This is partly due to the limited duration of that record and partly due to the limited instrument stabilities over the measurement-record timescale. Any secular trend is expected to be small given the level of stability that the Sun has shown during this time.

There have fortunately been no gaps in the space-borne TSI-measurement record since it began in 1978, but measurement continuity is only beneficial if the measurement stability uncertainties are sufficiently low. The measurement record is now of such length that instrument-stability uncertainties over this duration limit the detection of potential, small long-term solar trends. For TSI, assuming the measurement accuracies and stabilities in Table 2 are achievable, reliance on instrument accuracies instead is currently increasingly becoming the most viable approach for trend detection.

While the newer TSI instruments are approaching these needed levels of accuracy and stability, the earlier ones had much greater uncertainties, as do the current SSI measurements. Solar variability on secular timescales is thus not definitively known directly from the space-borne measurements because this record does not span the desired multi-decadal to -centennial time-range with the needed absolute accuracies. However, establishing a present-day benchmark of the solar irradiances with improved absolute accuracies will be highly beneficial to the future value of the record. This logic applies to the newer SSI record as well; the sooner the absolute accuracies in Table 2 are achieved, the sooner potential secular SSI variability can be detected.

The measurement record’s stability limitations are apparent in the differing individual-instrument-based composites shown in Fig. 44. Fröhlich (2009) reports no significant trend between the 1986 and 1996 solar minima in the PMOD composite and a decrease of 0012 ± 0.0008% year^−1^ between the solar minima occurring in 1996 and 2008. The small decreases to subsequent minima are within uncertainties in the composite, which are (perhaps optimisitically) claimed to be less than 0.0009% year^−1^. From the ACRIM composite, Willson (1997) and Willson and Mordvinov (2003) instead claim a 0.005% year^−1^ increase between the 1986 and 1996 minima with the more recent data in this composite showing an offsetting downward trend to the subsequent minimum. Their reported increase between the earlier two minima led to claims that solar variability was responsible for as much as 69% of the global temperature increase over the last century (Scafetta and West 2008); although that work was quickly discredited by the community (Foukal 2008; Schmidt 2008; Lean 2010). With the RMIB composite, Dewitte et al. (2004) report an increase between the 1986 and 1996 solar minima of 0.15 W m^−2^ ± 0.35 W m^−2^, so cannot conclude a significant trend. The reported composites’ trends between solar minima could be indicative of secular solar variability if correct; however not all composite trends can be correct since they are not consistent between the composites. These trend differences are instead more likely indicative of instrument instabilities and the differing means by which each composite is created rather than of a definitive secular-trend based on the space-borne measurement record.

The focus by these authors on the differences between solar minima is somewhat misleading and not necessarily an appropriate indicator of secular variability in the Sun’s output, however. While the eye is drawn to trends in solar-minima levels—knowing the solar maxima vary greatly with variations in well-quantified sunspot-number counts—there is no fundamental reason to expect identical irradiance values at each solar minimum. Solar cycles are known to overlap, with activity from a newer cycle often beginning prior to all activity from the previous cycle having ceased. Even if the Sun had a stable minimum level, variations in cycle start and end times and the resulting differences in overlap would cause apparent variations in the actual cycle-minimum irradiance reached. Rather than focus on minima (which could differ if the Sun has underlying secular variability or due to overlap times) or maxima (which differ due to solar-cycle activity, as indicated by sunspot number), a several-cycle trend is needed to determine secular variability. If there is a secular trend, it’s small enough that the current record is too short and lacks the long-term stability needed to detect it.

Instrument-to-instrument differences, such as cause the minimum-to-minimum variations between the TSI composites above, represent the collective’s stabilities but not those isolated to an individual instrument. A lower bound to an individual instrument’s stability uncertainty can be indicated by variations between consecutive releases of that instrument’s data. While updated versions of data are intended to include improvements based on new knowledge of the instrument’s status, version-to-version changes indicate that the former data version was inaccurate by (at least) the difference between the two. For prior-version data to be credible, new-to-old version-to-version differences should be contained within the stated uncertainties in the older data version. The August 2015, October 2015, and December 2015, VIRGO-data releases provide an example: Successive pairs of these three data releases show trend differences of − 0.0004% year^−1^ and  + 0.0006% year^−1^, lending doubt that the actual instrument stability is known to better than the differences of these levels. Even larger trend-changes were included between successive versions of ACRIM3 data in 2011, when reported TSI values in earlier data-versions showed an erroneous annual cycle of ~ 0.02% peak-to-peak due to lacking instrument thermal corrections. While such corrections presumably improve the later data versions, they do indicate the levels of errors in previous releases and may call into question whether all needed corrections are truly included in the latest release. (This lower-bound assessment of uncertainties, unfortunately, only applies to older-versioned data.)

Considerations such as these in addition to the disparate composites proposed by different researchers suggest that the current space-borne TSI measurement record has not achieved the needed  < 0.001% year^−1^ stability-uncertainty levels to definitively detect secular trends in solar-irradiance variability over the space-borne era record’s duration. Improvements to instrument stability and/or absolute accuracy are needed to discern such long-term variations at the level required for secular solar-variability detection. Some of the newer TSI instruments mentioned in Sect. 2.4 are reaching these levels of stability, giving hope that the present-day TSI measurements will be able to detect potential secular trends going forward in time.

### Secular variability on climate timescales

**Fig. 53:**
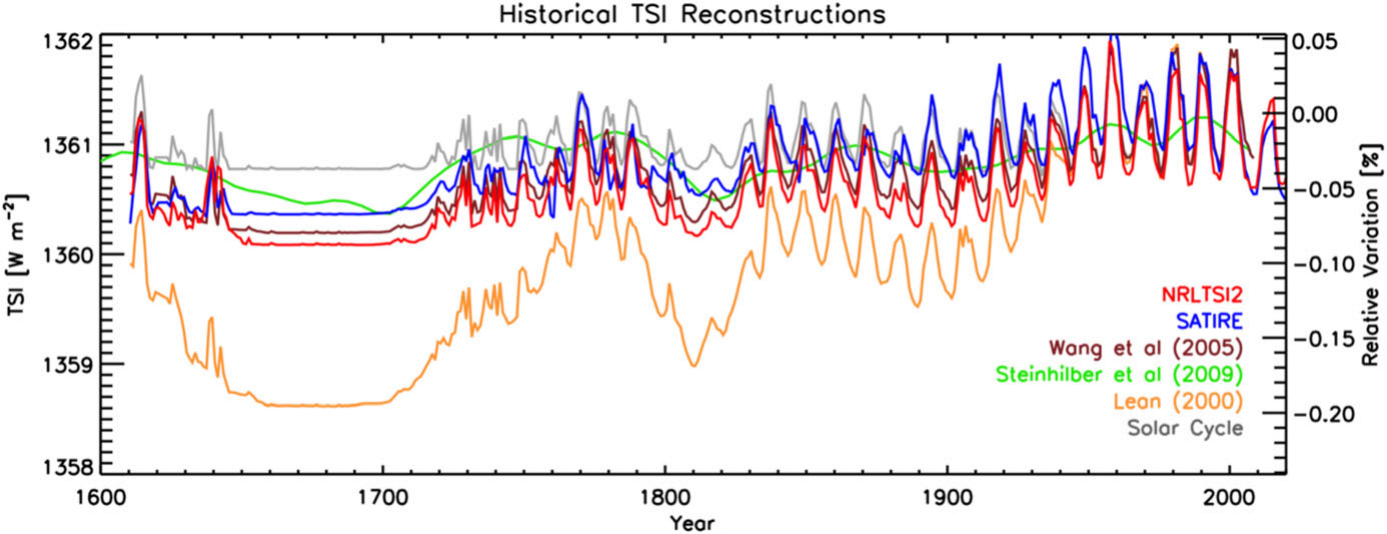
Historical TSI reconstructions. TSI reconstructions provide estimates of solar variability from the Maunder Minimum to the present and are largely based on the sunspot record (adapted from Kopp 2014)

Solar variability on multi-decadal and century timescales is yet more important but less known. No direct irradiance measurements exist over these durations, so solar-irradiance models, several of which are shown in Fig. 53, are relied upon for estimates.

Historical models of solar activity clearly show 11-year solar-cycle variations, and most have lower-frequency variations as well. These long-term fluctuations are not measured by the spacecraft-era irradiance instruments but come from other indicators of solar activity, such as facular proxies, sunspot records, auroral sightings, and cosmogenic isotopes (see Usoskin et al. 2015). Usoskin (2023) gives an excellent overview of the indicators of solar variability on millennial timescales.

One of the most prominent long-term comparisons is to the Maunder Minimum, a time of low sunspot activity from 1645 to 1715 that Eddy (1976) linked to Europe’s Little Ice Age. Knowledge of the solar-irradiance levels during that era is particularly relevant for understanding natural influences on Earth’s climate by correlating historical solar-irradiance variability estimates with observational surface-temperature records, which exist, particularly in northern Europe, over the last several centuries. Solar variations on these timescales are tenuous, however, as can be seen from the diversity of ranges in Fig. 53.

Even if not definitive, historical irradiance-reconstruction models provide the best indications of potential long-term solar variations that would need to be measurable for discerning, via direct observations, changes in the Sun’s radiant output that can influence climate over decadal and longer timeframes; hence such reconstruction models help determine accuracy- and stability-measurement requirements, leading to the results given in Sect. 2.2. The models in Fig. 53 suggest possible long-term solar-variability rates of 0.05% to 0.1% over nearly a century when entering or exiting the Maunder Minimum era. Direct detection of such secular changes thus requires instrument stability uncertainties less than ~ 0.001% year^−1^ and continual measurements. These stabilities are perhaps achieved by the newest on-orbit TSI instruments but are not met by the older TSI instruments or any SSI instruments contributing to the solar-irradiance record. In the absence of the needed stability uncertainties and measurement continuity, secular solar trends can also be detected via separate measurements over a long time-period if sufficient instrument absolute accuracy is achieved, as described in Sect. 2.2. Detecting long-term variations such as entering or exiting the Maunder Minimum would, in this case, require accuracies of ~ 0.01% and observations separated by a multi-decadal timespan to allow sufficient TSI signal-change to be detectable by disparate measurements of comparable accuracy.

### Variability due to Milankovitch orbital cycles

Variations in Earth-orbital parameters studied by Serbian geophysicist and astronomer Milutin Milanković in the 1920’s affect the magnitude and geographic distribution of solar energy reaching the Earth on 19,000- to 420,000-year timescales (Hays et al. 1976, with climate effects considered by Haigh 2011). Milankovitch orbital effects include: variations in the Earth’s obliquity (the tilt of the Earth’s spin-axis relative to the ecliptic, which largely determines seasonal- and regional-climate-variations); precession of the Earth’s spin-axis, which affects the time of year when seasons occur; the eccentricity of the Earth’s elliptical orbit around the Sun, which determines the Sun-Earth distance through the year; and apsidal precession, or the angular precession of the Earth’s elliptical orbit in the plane of the ecliptic. These have periods of ~ 21,000 years for apsidal precession, 26,000 years for axial precession, 41,000 years for obliquity, and 95,000 to 125,000 years for eccentricity-induced changes, which have a fundamental period of 420,000 years.

To distinguish the 1-AU-based reported-solar-irradiance definition from the orbital-induced solar-forcings on Earth-climate, the term “insolation” is used to refer to the Sun’s net radiant energy reaching the Earth regardless of its orbital distance from the Sun. These Milankovitch-cycle variations in the solar insolation are not intrinsic in the Sun’s radiative output itself and are well beyond the temporal scope of the direct solar-irradiance measurement record.

### Variability due to solar evolution

On billion-year timescales, the Sun changes by much larger amounts than at the (relatively) short timescales discussed above. Currently a G2V main-sequence star on the Hertzsprung–Russell diagram, the Sun varies due to stellar evolution as it burns its hydrogen supply along this sequence that is expected to last a total of roughly ten-billion years. The early Sun was approximately 72% as bright as it is at the present when it joined the main sequence about 4.6 billion years ago (Vardavas and Taylor 2007), and it has a current rate of increase in luminosity of 0.009% per million-years (Hecht 1994). At this rate, heating from the Sun will cause Earth's surface conditions to be like those of the present-day Venus in another 3.5 billion-years. Some billion or so years thereafter the Sun will leave the main sequence and transition to a red giant, increasing in radius by about 250 times and in luminosity by roughly 27,000 times but decreasing in surface temperature to 2600 K. Along the main sequence, increases in luminosity and radius over time are associated with increasing Sun-surface temperatures, meaning not only does the solar irradiance increase with time on this sequence, but the spectrum of the radiated light correspondingly shifts toward shorter wavelengths; although as the aging Sun becomes more quiescent, the solar-irradiance variability at those shorter wavelengths, which respond strongly to solar-surface magnetic-activity, is expected to decrease, reducing solar variability on rotational and solar-cycle timescales (for the longer rotational and solar-cycle timescales that the evolved Sun will have). Mentioned here only for completeness, these, again, are timescales well beyond those of the solar-irradiance measurement record.

## Future solar-irradiance record needs

Improvements to understanding the causes and effects of solar-irradiance variations require measurement improvements, better historical reconstructions of both solar-variability and Earth-climate records, coupled high- and low-altitude Earth-climate-model improvements incorporating the newer SSI measurements, and monitoring the SSI at stability levels needed for climate studies.

### Measurement improvements

Since all estimates of solar-irradiance variability—including historical reconstructions—are based on space-borne-era measurements, any improvements to this instrument-measurement record will directly benefit all derived uses of the record. Those improvements directly improve historical estimates of solar variations and benefit the future record via better present-day benchmarking of the solar irradiance. Irradiance-record improvements for climate studies will come from improved measurement stabilities and accuracies across the spectral range, better ground-based calibration-facilities, and lengthened measurement-record durations. Improved instrument sensitivities will help understanding short-term solar variabilities.

#### Stabilities

The greatest imminent improvements are likely from measurement stabilities that meet or exceed the requirements given in Table 2. As discussed in Sect. 4.4, the current space-borne measurement-record lacks the needed stability to discern secular variations in the solar irradiance, and these timescales are the most relevant for climate studies. Definitively measuring potential long-term variations in the Sun’s output via better instrument stabilities would greatly advance present understandings of the possible range of long-term solar variability. Alternatively, showing that the Sun does not change on secular timescales would limit the long-term variations in current reconstructions and change estimated climate sensitivities based on correlations with historical-climate records. Stability improvements are expected from new materials and flight radiometers (see Sect. 5.2).

#### Accuracies

The next-greatest improvement to the measurement record is via improving absolute accuracy. Lower TSI-measurement uncertainties on an absolute scale will establish current TSI values as a benchmark against which distant future measurements can be related to enable long-term trend-detection even in the absence of continual intervening solar measurements. Establishing such a benchmark via improved absolute accuracy also reduces severity of a gap in the currently uninterrupted TSI data-record, as subsequent instruments having similar measurement-accuracy could reduce the uncertainties in solar variability across the duration of that gap. Lower measurement-uncertainties also decrease the timespan needed for trend detection. Future SITSats (see Sect. 5.2) promise improved accuracies.

Similar accuracy improvements would also benefit the SSI record to help it achieve the measurements requirements in Table 2. Those goals are more difficult at the lower-power levels of the SSI measurements and are not imminent.

Improved ground-based reference calibration facilities are helping improve both TSI- and SSI-instrument accuracies. The TRF (Kopp et al. 2007) is currently the international reference-standard for calibrations, validations, and diagnostics of TSI-instrument accuracies under flight-like conditions, whereby both optical power and irradiances of a TSI instrument under test are compared to nearly identical such measurements with a NIST-calibrated radiometer at full solar-power levels while under vacuum. It is from comparisons and diagnostics on this facility that many of the erroneously high readings of older TSI instruments were confirmed and have now been corrected (see Sect. 2.4.12). A similar facility has been built for SSI instruments and was used in the calibrations of the TSIS-1/SIM and the CSIM (Richard et al. 2019, 2020). Improvements in these facilities or the construction of similar new facilities will directly improve spaceflight measurement accuracies, helping benchmark the present-day Sun.

#### Durations

Since long-term solar trends and the frequency of occurrences of grand minima or maxima are largely unknown, simply lengthening the space-borne measurement record will improve knowledge of solar variability (or the lack thereof). Absolute accuracy better detects long-term trends over greater durations (see Sect. 2.2 and Sect. 4.4). A greater duration also increases the likelihood of sampling eras when the Sun behaves abnormally, such as transitioning to a grand minimum or maximum. A lengthened record is most valuable in conjunction with improved measurement accuracies and stabilities.

#### Precision

As solar-irradiance reconstruction models improve in fidelity, correlating the effects of those models with the measurement record will benefit from improved short-term precision (aka “resolution” or “sensitivity”) in the current measurements. Low-noise techniques, such as the phase-sensitive detection methods implemented with the TIM and SIM instruments, improve instrument precision by reducing measurement sensitivity to surrounding thermal-background and electrical influences that can introduce measurement artifacts. Lower-noise photodiode detectors in the TSIS-1/SIM and the CSIM have also improved recent measurement precision and response time, helping provide faster spectral scans.

### Instrument improvements

Instrument-specific improvements that will benefit the solar-irradiance record apply to both TSI and SSI instruments.

*Aperture Layout*: Instrument designs with the light-limiting precision aperture at the front of the instrument (see Sect. 2.4.5) have been adopted by almost all subsequent instruments. The inverted-cone geometry (i.e., cone tip facing toward the entering sunlight) of the ERB, VIRGO, and PREMOS radiometers has been foregone in favor of Sun-facing conical-cavity-mouth designs starting with the ACRIMs.

*Smaller, Cheaper Instruments*: Compact and lower-cost instruments will reduce the risk of data gaps, since these can be fabricated and calibrated on shorter timeframes and with less financial commitment from funding agencies. The CLARA (Sect. 2.4.11.6) is an early attempt at a more compact TSI instrument, although the tradeoffs of larger thermal fluctuations in smaller instruments has hindered that instrument’s data release. The CTIM (Sect. 2.4.10) and CSIM (Sect. 2.5.2.14) have been more successful in producing and releasing useful science results in even smaller sizes.

*Materials*: Material advances are providing improvements. Carbon nanotubes have been used for irradiance measurements on the CTIM and CSIM technology-demonstration missions. These nanotubes have higher absorptivities, better thermal conductivities, and better robustness against degradation from exposure to hard solar radiation than traditional radiometer surfaces (see Tomlin et al. 2015), all of which should improve stabilities compared to the currently used absorptive materials (paints and NiP) in radiometers. Their high absorptivities enable radiometer designs that do not rely on geometry for high absorptivity, such as via traditional conical cavities in TSI instruments, and so enable much smaller and faster-responding designs.

*Cryogenic Radiometry and SITSats*: All space-based solar-irradiance instruments have operated near ambient temperature, although the gold standard in lab-based optical-power measurement has traditionally been the cryogenic radiometer. Future SI-Traceable Satellites (SITSats) such as the UK Space Agency’s Traceable Radiometry Underpinning Terrestrial- and Helio-Studies (TRUTHS; Fox and Green 2020) will include the Cryogenic Solar Absolute Radiometer (CSAR; Martin and Fox 1994) to acquire direct TSI and SSI measurements. This primary reference is intended to achieve 0.01% uncertainties in TSI measurements, meeting the requirements in Table 2.

## Summary

The space-borne measurement-record provides the best current knowledge of the net energy driving the Earth-climate system. Differences between this incident total solar irradiance and the outgoing reflected-shortwave and thermal-emitted radiances give the Earth’s energy imbalance, which determines climate change. Measurement uncertainties of the total solar irradiance are much lower than those of any other on-orbit radiometric measurement, helping reduce uncertainties in direct knowledge of the Sun’s long-term variability and the Earth-system energy-balance. Spectral solar irradiance measurements help understand where solar radiation is absorbed in the Earth’s climate system and how those regions are affected by solar variability on short-term timescales. This article provides an overview of solar-irradiance measurements, focusing on the space-based era, and reviews the designs, advantages, and uncertainties of most total- and spectral-irradiance instruments flown. Measurement requirements are given. Solar variability on multiple timescales is discussed, and reference spectra are reviewed. Improvements in accuracy, stability, and spectral-variability to the ongoing solar-irradiance measurement record will help determine the magnitudes of solar variability on long-term timescales and refine both climate models and knowledge of climate sensitivities to solar forcing.
